# Supplementary material for: DDCM: A Computational Strategy for Drug Repositioning Based on Support-Vector Regression Algorithm
Source: Int J Mol Sci. 2024 May 12;25(10):5267. doi: 10.3390/ijms25105267 (PMC11121335; doi:10.3390/ijms25105267)
Supplement: Supplementary file 1 [file ijms-25-05267-s001.zip › File S1.pdf]

# DDCM：A computational strategy for drug repositioning based on support vector regression algorithm

**Manyi Xu [1,†], Wan Li [1,†], Jiaheng He [1,†], Yahui Wang [1], Junjie Lv [1], Weiming He [2], Lina Chen [1,*], and Hui Zhi [1,*]**

## Supplementary Materials

**Table S1.** Training dataset

| Data type | Source | Number |
|---|---|---|
| disease | DO | 710 |
| drug | DrugBank | 1775 |
| disease-drug | CTD/DrugBank | 19632 |
| drug pathway | KEGG/CTD | 2187 |
| drug-target gene | DrugBank | 50876 |
| disease pathway | KEGG/CTD | 2339 |
| disease pathogenic gene | OMIM/DO/CTD | 5715 |

**Table S2.** Literature Validation of Potential Therapeutic Drugs for NSCLC.

| Potential therapeutic drugs | Generic Name | Literature |
|---|---|---|
| DB02424 | Geldanamycin | [1] |
| DB01394 | Colchicine | |
| DB01177 | Idarubicin | [2] |
| DB13811 | Oblimersen | [3, 4] |
| DB06810 | Plicamycin | |
| DB01254 | Dasatinib | [5-8] |
| DB06176 | Romidepsin | [9, 10] |
| DB00590 | Doxazosin | |
| DB11919 | 6-O-benzylguanine | [11] |
| DB00448 | Lansoprazole | [12] |
| DB01099 | Flucytosine | |
| DB04815 | Clioquinol | |
| DB08901 | Ponatinib | [13-15] |
| DB05220 | Alisertib | [16, 17] |
| DB04944 | Acadesine | |
| DB11890 | Cilengitide | [18] |
| DB06444 | Dexanabinol | |
| DB11648 | Afuresertib | |
| DB01179 | Podofilox | |
| DB05088 | Tetrathiomolybdate | [19] |
| DB01162 | Terazosin | |
| DB00352 | Tioguanine | [20] |
| DB00338 | Omeprazole | [21] |
| DB09350 | Piperonyl butoxide | |
| DB00947 | Fulvestrant | [22, 23] |

**Table S3.** The cerebrovascular disease-associated gene set has literature validating it as a therapeutic target for the disease.

| Gene Family | Literature |
|---|---|
| GRIN | [24] |
| CALM | [25] |
| OPR | [26] |
| MAO | [27-29] |
| ADR | [30] |
| DRD | [31] |
| HTR | [32] |

**Table S4.** Comparison of prediction performance of different methods

| Method | AUC |
|---|---|
| SVM | $9.84e-01$ |
| KNN | $9.50e-01$ |
| RW | $8.91e-01$ |
| DDCM | $9.96e-01$ |

A

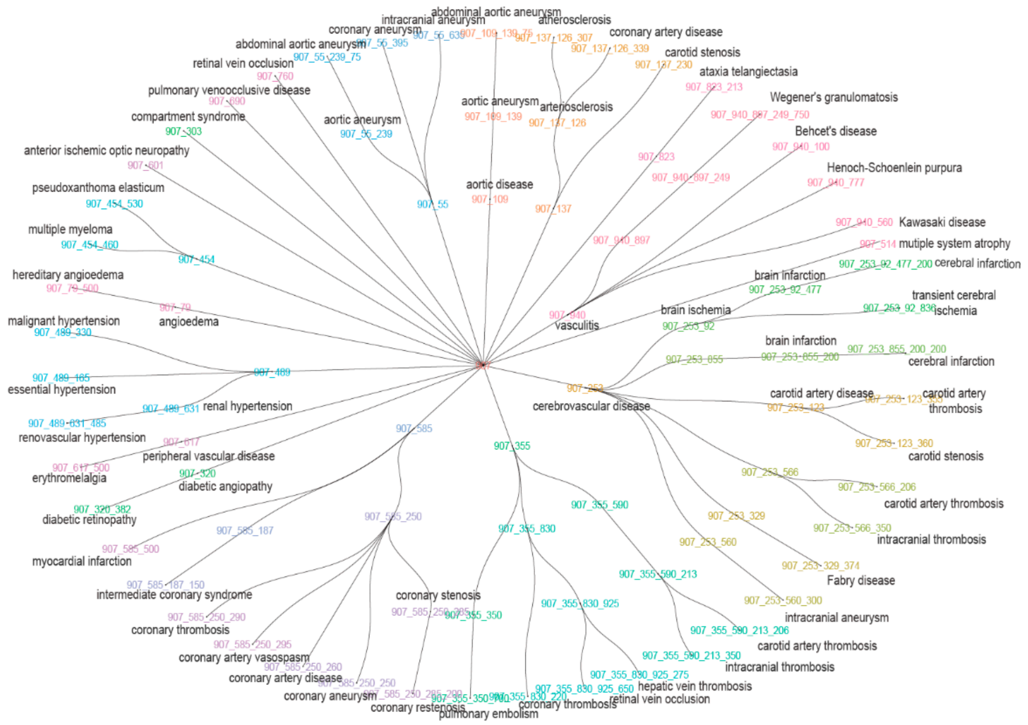


B

cerebrovascular disease

| | DrugBank ID | Score |
|---|---|---|
| 1 | DB00788 | 0.790 |
| 2 | DB01191 | 0.559 |
| 3 | DB01191 | 0.556 |
| 4 | DB01191 | 0.540 |
| 5 | DB01191 | 0.530 |
| 6 | DB00579 | 0.458 |
| 7 | DB00579 | 0.451 |
| 8 | DB00579 | 0.441 |
| 9 | DB00261 | 0.375 |
| 10 | DB00261 | 0.366 |
| ... | ... | ... |

vasculitis

| | DrugBank ID | Score |
|---|---|---|
| 1 | DB00477 | 0.699 |
| 2 | DB00477 | 0.696 |
| 3 | DB00477 | 0.690 |
| 4 | DB01140 | 0.646 |
| 5 | DB00816 | 0.570 |
| 6 | DB00816 | 0.567 |
| 7 | DB00261 | 0.535 |
| 8 | DB00261 | 0.512 |
| 9 | DB05015 | 0.479 |
| 10 | DB00679 | 0.492 |
| ... | ... | ... |

coronary thrombosis

| | DrugBank ID | Score |
|---|---|---|
| 1 | DB01362 | 0.612 |
| 2 | DB01362 | 0.607 |
| 3 | DB00650 | 0.384 |
| 4 | DB00650 | 0.377 |
| 5 | DB09313 | 0.362 |
| 6 | DB00227 | 0.322 |
| 7 | DB00694 | 0.298 |
| 8 | DB00717 | 0.293 |
| 9 | DB00694 | 0.291 |
| 10 | DB00694 | 0.290 |
| ... | ... | ... |

C

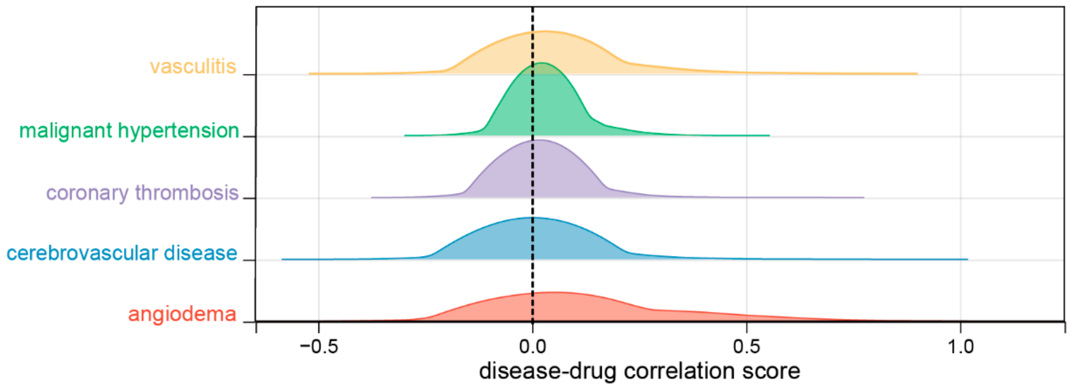


**Figure S1. (A)** The distribution of the disease data adopted in the MeSH branch C14.907 in the branch. **(B)** The disease-drug correlation score for a certain disease calculated using the DDCM method. (The figure shows the partial disease-drug correlation score results for cerebrovascular disease, vasculitis and coronary thrombosis .) **(C)** The distribution of disease-drug correlation scores for different diseases (The figure shows the distribution of disease-drug correlation scores for five

diseases, vasculitis, malignant hypertension, coronary thrombosis, cerebrovascular disease, and angioedema.)

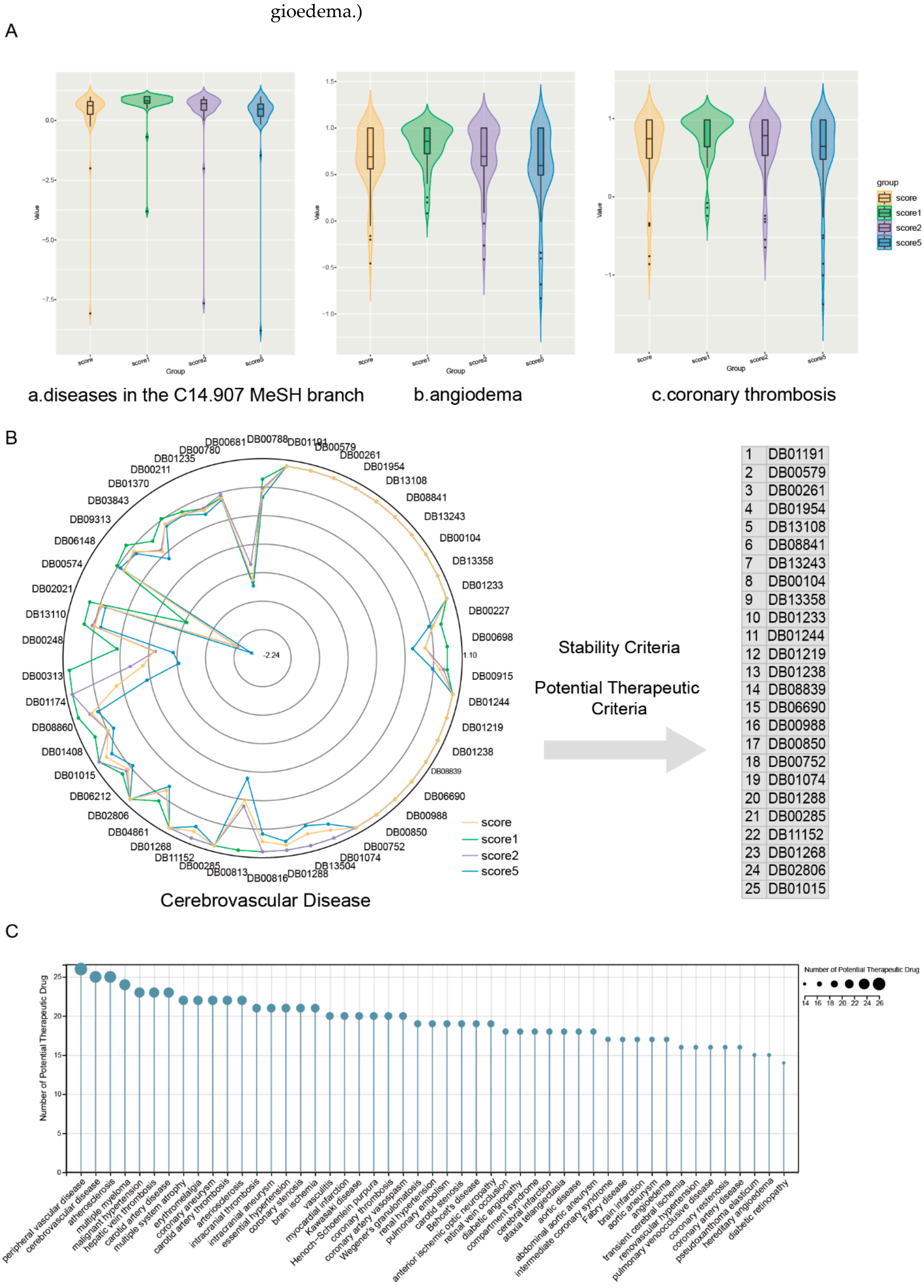


**Figure S2. (A)** a The overall distribution of disease-drug correlation scores for the drugs of interest for the selected diseases in the C14.907 branch of MeSH before and after random perturbation. b and c the distribution of disease-drug correlation scores for the relevant drugs in this branch for angioedema and coronary thrombosis, respectively, before and after receiving random perturbation. **(B)** The radar plot of stable drug candidates for cerebrovascular disease and the potential

therapeutic drug criteria, where drugs with stability scores close to 1 multiple times are considered relatively stable. After the potential therapeutic criteria a total of 25 potential therapeutic drugs for final cerebrovascular disease were identified. **(C)** the number of potential therapeutic drugs for a disease.

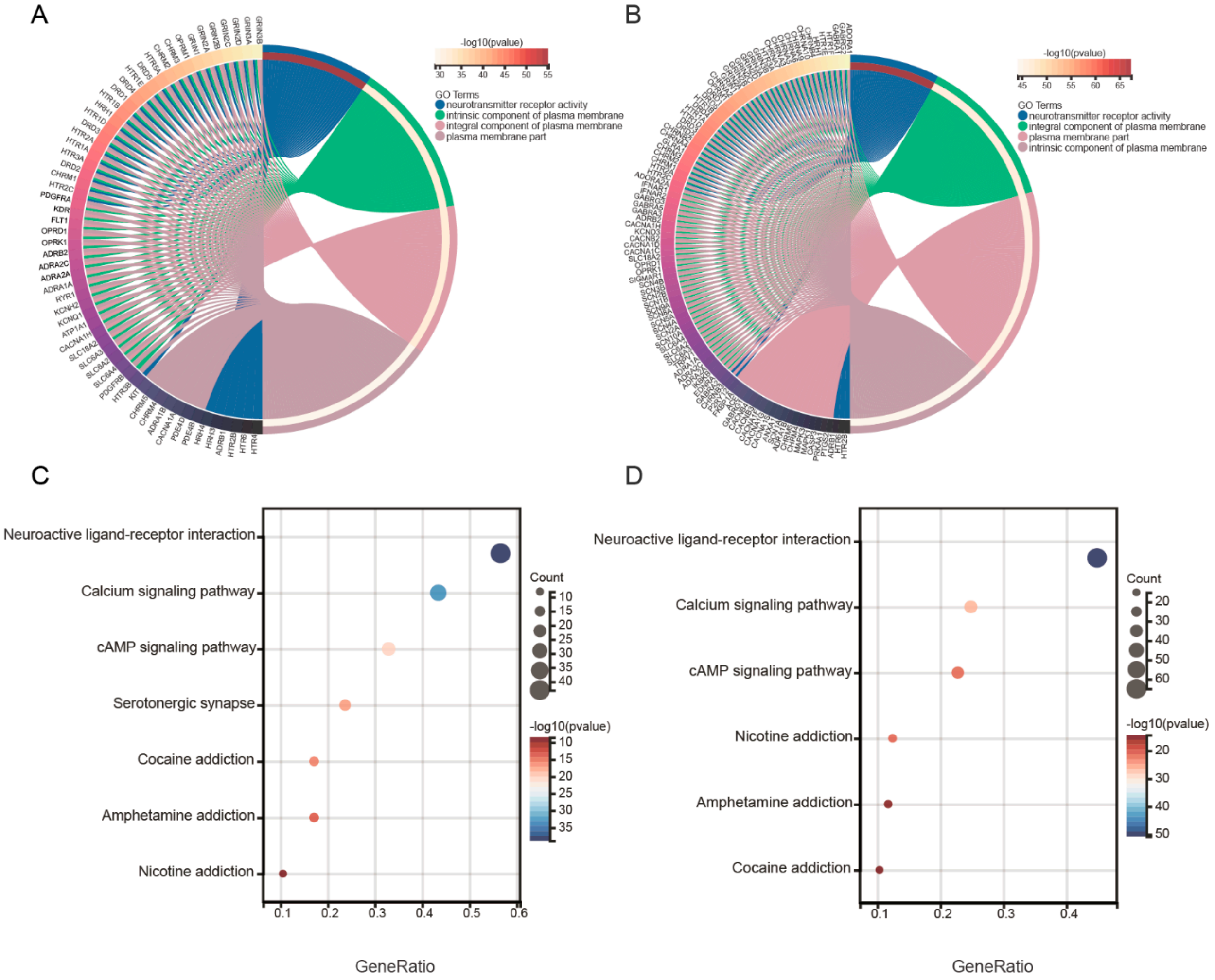


**Figure S3. (A)** and **(B)** The GO enrichment analysis of the drug targets of predicted potential therapeutic drugs and the drug targets of known therapeutic drugs for cerebrovascular disease, respectively. **(C)** and **(D)** The KEGG enrichment analysis of the drug targets of predicted potential therapeutic drugs and the drug targets of known therapeutic drugs for cerebrovascular disease, respectively.

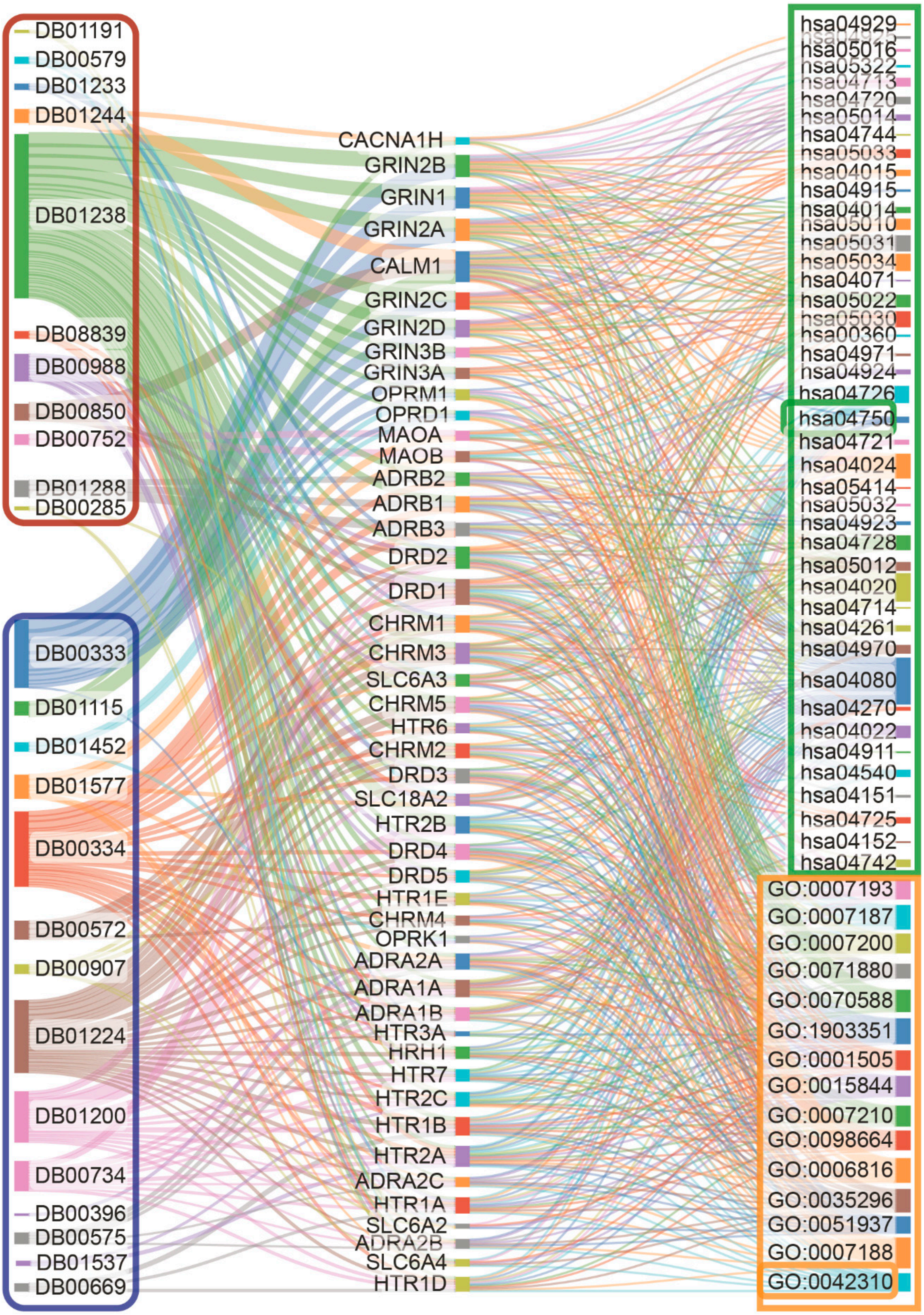


**Figure S4.** The Sankey plot shows the partial results of GO and KEGG enrichment of the set of genes predicted by the DDCM method for cerebrovascular disease after intersecting and then merging the potential therapeutic and known therapeutic drugs with the disease pathogenic genes, respectively. The red and blue rounded rectangles are the predicted potential therapeutic drugs and known therapeutic drugs for cerebrovascular disease, respectively. The green and yellow rectangles are the KEGG pathways and GO terms that are significantly enriched for this gene set, respectively.

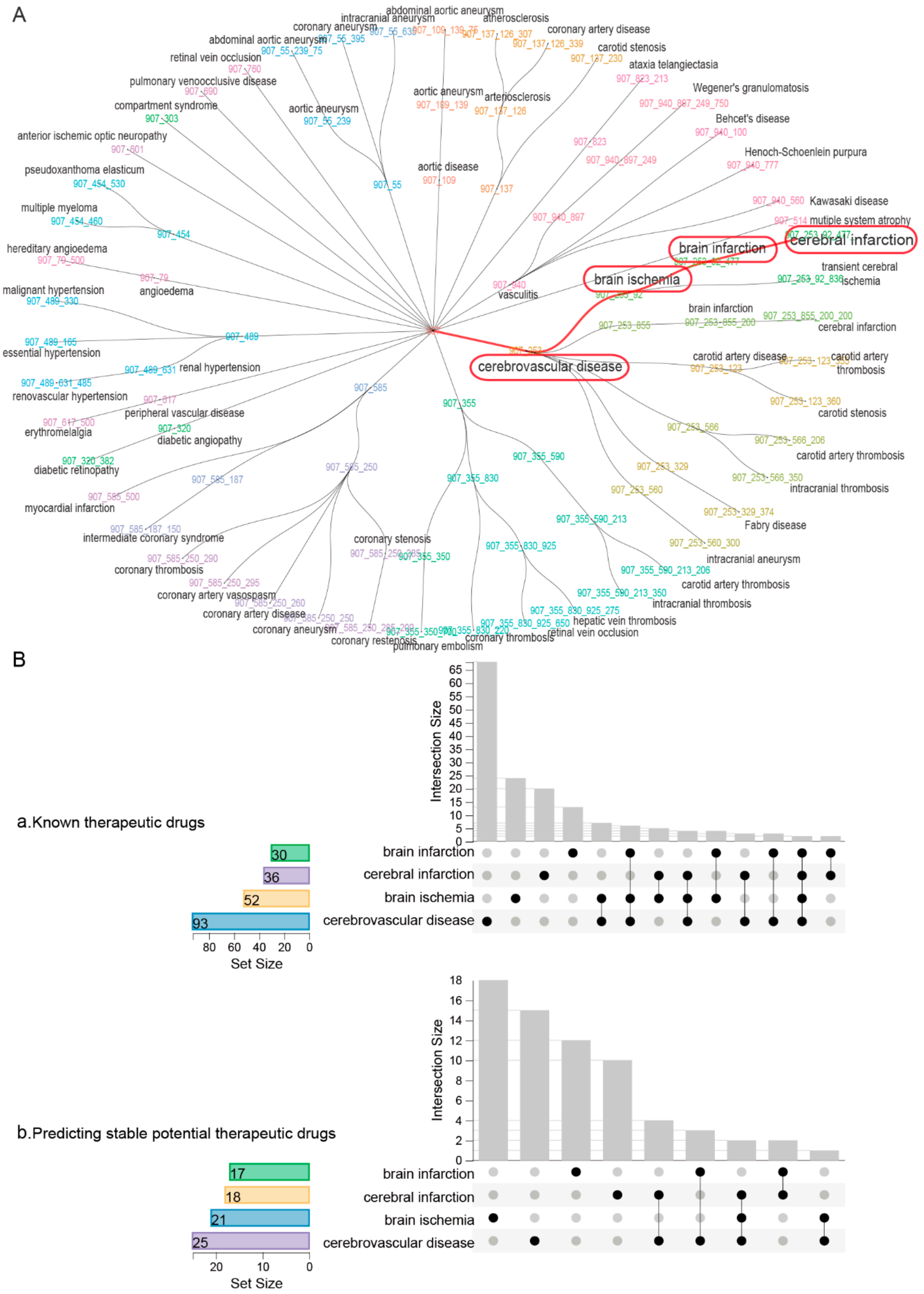


**Figure S5**. **(A)** The relationship between four cardiovascular diseases (cerebrovascular disease, brain ischemia, brain infarction and cerebral infarction) under the C14.907 branch of MeSH. **(B)** a. Overlap of known therapeutic drugs for the four diseases. b. Overlap of the number of potential therapeutic drugs predicted by the DDCM method for the four diseases.

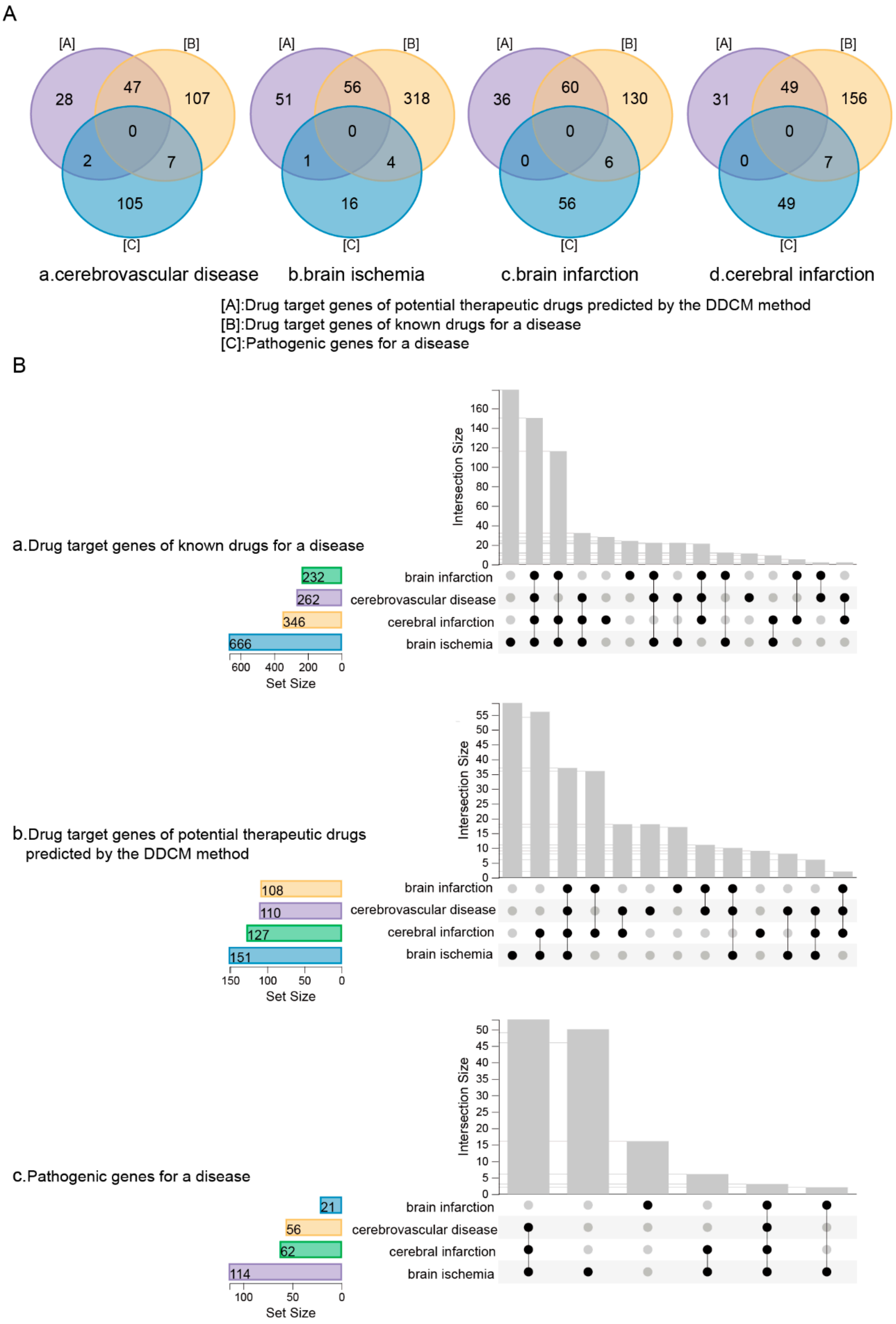


**Figure S6.** (**A**)(a-d) The venn diagrams indicate the degree of overlap of the predicted potential therapeutic drug targets, drug targets of known drugs, and disease pathogenic genes for cerebrovascular disease, brain ischemia, brain infarction and cerebral infarction, respectively. **(B)**(a-c) The diagrams represents the overlap of drug targets of known therapeutic drugs, drug targets of predicted potential therapeutic drugs, and pathogenic genes for each of the four diseases (cerebrovascular disease, brain ischemia, brain infarction and cerebral infarction).

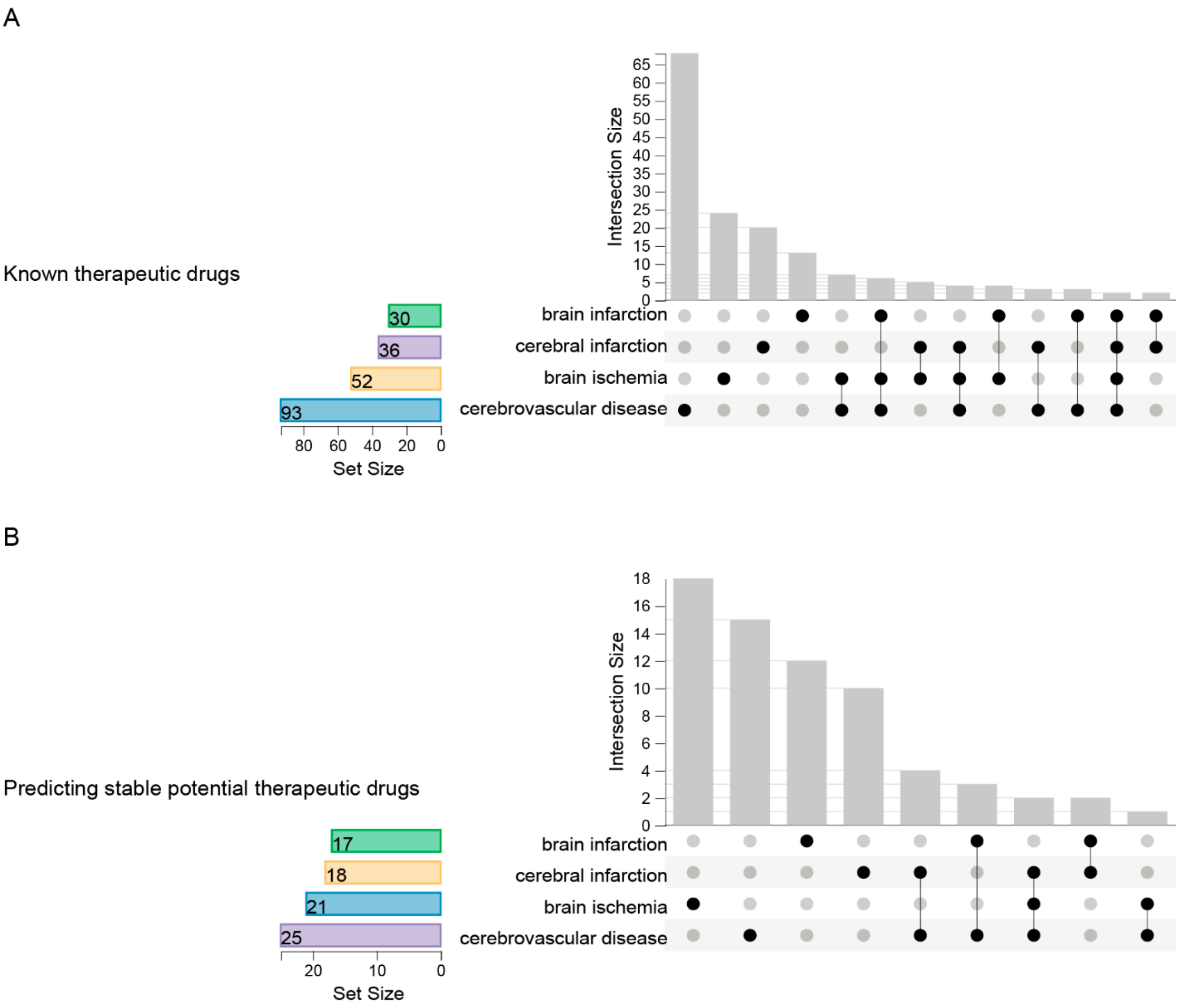


**Figure S7. (A)**Overlap of known therapeutic drugs for the four diseases. **(B)** Overlap of the number of potential therapeutic drugs predicted by the DDCM method for the four diseases.

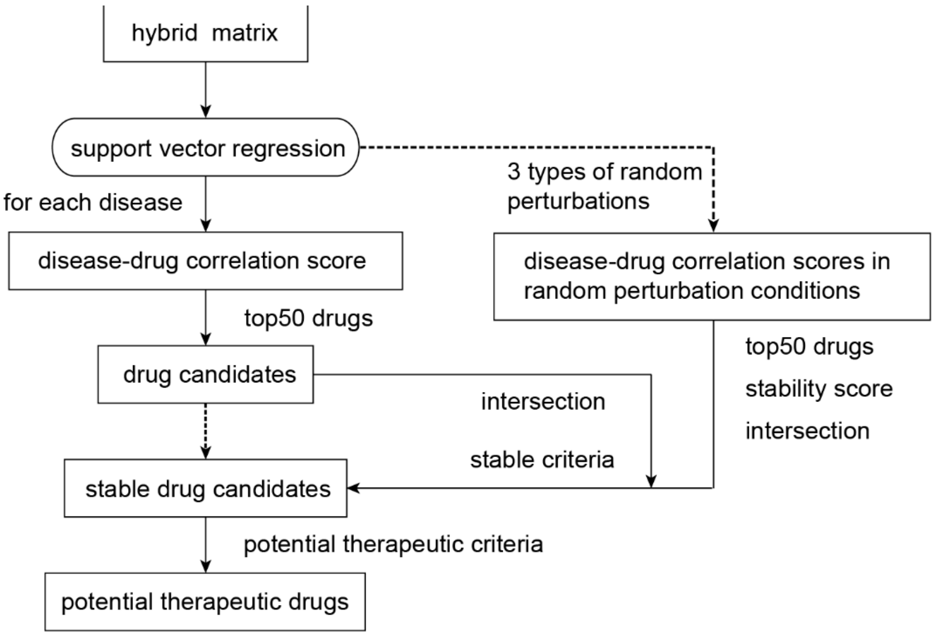


**Figure S8.** Stepwise screening process for potential therapeutic drugs for each disease.

**Document S1**

The whole set of findings on vascular disease.

## 1. Results

### *1.1. Screening for potential therapeutic drugs for vascular disease*

To further explore the generalizability of the drug repositioning strategy, we applied this method to 49 vascular diseases from MeSH branch (C14.907) (Supplementary Figure S1, File S5 1_C14.907_CorScore, File S5 2_stability_scores_boxplot). Around these diseases, we mainly analyzed cerebrovascular disease, 25 potential therapeutic drugs were eventually identified (Supplementary Figure S2; File S2 Table S6). The GO enrichment analysis of these screened potential therapeutic drug targets showed that the drug target genes of the predicted drugs for the cerebrovascular disease were mainly enriched in some GO terms relateared to activity of some ions, proteins, and channels. In addition, information on drug targets of known therapeutic drugs for disease and disease pathogenic genes was noticed. Similarly, it could be seen that analogous enrichment trends in GO enrichment analysis of drug targets of known drugs. This situation was also manifested in the KEGG enrichment analysis. It could also be noticed that the drug targets of screened potential therapeutic drugs and known therapeutic drugs for the disease were mainly enriched in some pathways related to drug addiction and signal transduction (Supplementary Figure S3).

For cerebrovascular disease, the drug targets of its associated drugs with pathogenic gene enrichment to the KEGG pathway with GO terms such as hsa04750: Inflammatory mediator regulation of TRP channels, GO:0042310: vasoconstriction, etc. (Supplementary Figure S4) had corresponding literature support. Numerous studies have shown that certain stimuli contribute to the development of vascular disease. Atherosclerosis is thought to be the main factor responsible for most cardiovascular diseases. The role of infection is thought to provide a key inflammatory stimulus[33]. Nitsa A et al. proved that vitamin D deficiency has been associated with activation of the pro-inflammatory mechanism, promoting atherogenesis[34].Kim JH et al. studied and learned that greater peripheral vasoconstriction with mental stress, denoted by a low sPAT ratio, is associated with a higher risk of adverse cardiovascular outcomes in patients with coronary artery disease[35].

At the genetic level, it was observed that the genomes associated with cerebrovascular disease could be broadly classified into several gene families, almost every gene family had been validated in the literature as causative genes or therapeutic targets associated with vascular disease (Supplementary TableS3).

Esteban G et al. proved that JL72 is a good and selective MAO-A inhibitors, thus possible modulators of the monoaminergic neurotransmission that behaves as a multitarget ligand able to modulate monoaminergic transmission and besides showing an anti-inflammatory profile, both pathways known to be altered in neurological disorders. This suggests that JL72 is a promising lead compound for further development of drugs to be used in the therapy of cerebrovascular and neurological diseases[27]. From our screened potential therapeutic drugs for cerebrovascular disease: Tranylcypromine (DB00752) is a monoamine oxidase inhibitor. Tranylcypromine has two targets: MAO-A and MAO-B. This monoamine oxidase inhibitor is effective in the treatment of major depression, dysthymic disorder, and atypical depression. It also is useful in panic and phobic disorders (From AMA Drug Evaluations Annual, 1994, p311). This suggested that tranylcypromine may hold promise as a therapeutic drug for cerebrovascular diseases from pharmacological aspects such as monoamine oxidase inhibitors.

Combined with the above analysis, this proved the accuracy and stability of the prediction results from a functional perspective. The pharmacotherapeutic mechanisms of some drugs could be explained by the rich functional classes of their drug target genes.

On the other hand, for screened potential therapeutic drugs for a disease, by searching PubMed to find relevant literature, we found that some of the screened potential therapeutic drugs had been shown to have a therapeutic effect on the disease in animal experiments or there were relevant experiments to support the indirect therapeutic promotion of the drug for the treatment of the disease. For instance, in cerebrovascular disease 's prediction of stable drug outcomes by the DDCM method, we found that these drugs were confirmed by the corresponding literature through a search of the literature.Tyramine(DrugBankID:DB08841[36]),Dantrolene(DrugBankID:DB01219[37]),Aripiprazole(DrugBankID:DB01238 [38]).

### *1.2. Drug research of similar vascular disease in MeSH branches*

For these cardiovascular diseases in the MeSH branch, four diseases from one of the sub-branches of this branch were selected for analysis from different perspectives. These four diseases were cerebrovascular disease, brain ischemia, brain infarction and cerebral infarction in the same branch based on the hierarchical relationship of the MeSH branch (Supplementary Figure S5).

The overlap between the drug target genes of the predicted potential therapeutic drugs and the target genes of the known drugs and the genes of the diseases was examined for the four diseases, and it could be found that the degree of overlap between the predicted potential therapeutic drugs and the drug targets of the known drugs for these four similar diseases was relatively large, while the degree of overlap with the pathogenic genes was smaller (Supplementary Figure S6A a-d). This indicated that the therapeutic mechanisms of both predicted potential therapeutic drugs and known therapeutic drugs were similar, while differed from the pathogenic mechanisms of the diseases. In addition, the drug targets of the predicted potential therapeutic drugs, the drug targets of the known therapeutic drugs, and the pathogenic genes of the four diseases overlapped to some extent (Supplementary Figure S6B a-c), indicating that the four diseases were similar in their respective therapeutic and pathogenic mechanisms, which was further illustrated by the similarity of the four diseases since they were taken from the same branch of MeSH.

By predicting potential therapeutic drugs for these diseases, common therapeutic drugs could be found between these four cardiovascular diseases. Also, there was some degree of overlap in the known therapeutic drugs for these diseases (Supplementary Figure S7 A,B).

Among the known drugs for the four diseases, where Aspirin (DB00945) and Nimodipine (DB00393) are the known drug for cerebrovascular disease, brain ischemia, brain infarction, and cerebral infarction. (Aspirin is a salicylate used to treat pain, fever, inflammation, migraines, and reducing the risk of major adverse cardiovascular events. Nimodipine is a calcium channel blocker used to improve neurological outcomes in patients with subarachnoid hemorrhage due to a ruptured intracranial aneurysm.)

Among the predicted potential therapeutic drugs for the four diseases, Dopamine (DB00988) was one of the predicted potential therapeutic drugs common to cerebrovascular disease, brain ischemia, and cerebral infarction. Dopamine is a catecholamine neurotransmitter used to treat hemodynamic imbalances, poor perfusion of vital organs, low cardiac output, and hypotension. Frishman WH et al proved that dopamine is a parenteral agent that selectively activates both DA1 and beta 2 adrenergic receptors and is being evaluated in patients with CHF and in individuals with postoperative left ventricular dysfunction. A group of selective DA2 receptor agonists is being evaluated as long-term treatment for systemic hypertension[39].

We have demonstrated a degree of overlap between the potential therapeutic drugs predicted by the DDCM approach for these four diseases under the same MeSH branch, as well as confirmed by certain literature.

## References

1. Shimamura, T.; Perera, S.A.; Foley, K.P.; Sang, J.; Rodig, S.J.; Inoue, T.; Chen, L.; Li, D.; Carretero, J.; Li, Y.C.; et al. Ganetespib (STA-9090), a nongeldanamycin HSP90 inhibitor, has potent antitumor activity in in vitro and in vivo models of non-small cell lung cancer. *Clin. Cancer Res.* **2012**, *18*, 4973-4985.
2. Ardizzoni, A.; Pronzato, P.; Repetto, L.; De Palma, M.; Canobbio, L.; Gulisano, M.; Fusco, V.; Rosso, R.; Ganzina, F. Phase II trial of oral idarubicin in advanced non-small cell lung cancer (NSCLC). *Cancer Investig.* **1988**, *6*, 409–411. https://doi.org/10.3109/07357908809080069.
3. Klasa, R.J.; Gillum, A.M.; Klem, R.E.; Frankel, S.R. Oblimersen Bcl-2 antisense: Facilitating apoptosis in anticancer treatment. *Antisense Nucleic Acid Drug Dev.* **2002**, *12*, 193–213. https://doi.org/10.1089/108729002760220798.
4. Frankel, S.R. Oblimersen sodium (G3139 Bcl-2 antisense oligonucleotide) therapy in Waldenstrom's macroglobulinemia: A targeted approach to enhance apoptosis. *Semin. Oncol.* **2003**, *30*, 300–304. https://doi.org/10.1053/sonc.2003.50041.
5. Kruser, T.J.; Traynor, A.M.; Wheeler, D.L. The use of single-agent dasatinib in molecularly unselected non-small-cell lung cancer patients. *Expert Opin. Investig. Drugs* **2011**, *20*, 305–307. https://doi.org/10.1517/13543784.2011.550873.
6. Johnson, F.M.; Saigal, B.; Talpaz, M.; Donato, N.J. Dasatinib (BMS-354825) tyrosine kinase inhibitor suppresses invasion and induces cell cycle arrest and apoptosis of head and neck squamous cell carcinoma and non-small cell lung cancer cells. *Clin. Cancer Res.* **2005**, *11*, 6924–6932. https://doi.org/10.1158/1078-0432.Ccr-05-0757.
7. Gold, K.A.; Lee, J.J.; Harun, N.; Tang, X.; Price, J.; Kawedia, J.D.; Tran, H.T.; Erasmus, J.J.; Blumenschein, G.R.; William, W.N.; et al. A phase I/II study combining erlotinib and dasatinib for non-small cell lung cancer. *Oncologist* **2014**, *19*, 1040–1041. https://doi.org/10.1634/theoncologist.2014-0228.
8. *Haura, E.B.; Tanvetyanon, T.; Chiappori, A.; Williams, C.; Simon, G.; Antonia, S.; Gray, J.; Litschauer, S.; Tetteh, L.; Neuger, A.; et al.* Phase I/II study of the Src inhibitor dasatinib in combination with erlotinib in advanced non-small-cell lung cancer. *J. Clin. Oncol.* **2010**, *28*, 1387–1394. https://doi.org/10.1200/jco.2009.25.4029.

9. Gerber, D.E.; Boothman, D.A.; Fattah, F.J.; Dong, Y.; Zhu, H.; Skelton, R.A.; Priddy, L.L.; Vo, P.; Dowell, J.E.; Sarode, V.; et al. Phase 1 study of romidepsin plus erlotinib in advanced non-small cell lung cancer. *Lung Cancer* **2015**, *90*, 534–541. https://doi.org/10.1016/j.lungcan.2015.10.008.
10. Schrump, D.S.; Fischette, M.R.; Nguyen, D.M.; Zhao, M.; Li, X.; Kunst, T.F.; Hancox, A.; Hong, J.A.; Chen, G.A.; Kruchin, E.; et al. Clinical and molecular responses in lung cancer patients receiving Romidepsin. *Clin. Cancer Res.* **2008**, *14*, 188–198. https://doi.org/10.1158/1078-0432.Ccr-07-0135.
11. Egyházi, S.; Edgren, M.R.; Hansson, J.; Kröckel, D.; Mannervik, B.; Ringborg, U. Role of O6-methylguanine-DNA methyltransferase, glutathione transferase M3-3 and glutathione in resistance to carmustine in a human non-small cell lung cancer cell line. *Eur. J. Cancer* **1997**, *33*, 447–452. https://doi.org/10.1016/s0959-8049(97)89021-1.
12. Zhao, X.; Zhang, N.; Huang, Y.; Dou, X.; Peng, X.; Wang, W.; Zhang, Z.; Wang, R.; Qiu, Y.; Jin, M.; et al. Lansoprazole Alone or in Combination With Gefitinib Shows Antitumor Activity Against Non-small Cell Lung Cancer A549 Cells in vitro and in vivo. *Front. Cell Dev. Biol.* **2021**, *9*, 655559. https://doi.org/10.3389/fcell.2021.655559.
13. Ren, M.; Hong, M.; Liu, G.; Wang, H.; Patel, V.; Biddinger, P.; Silva, J.; Cowell, J.; Hao, Z. Novel FGFR inhibitor ponatinib suppresses the growth of non-small cell lung cancer cells overexpressing FGFR1. *Oncol. Rep.* **2013**, *29*, 2181–2190. https://doi.org/10.3892/or.2013.2386.
14. Arbour, K.C.; Manchado, E.; Bott, M.J.; Ahn, L.; Tobi, Y.; Ni, A.A.; Yu, H.A.; Shannon, A.; Ladanyi, M.; Perron, V.; et al. Phase 1 Clinical Trial of Trametinib and Ponatinib in Patients With NSCLC Harboring KRAS Mutations. *JTO Clin. Res. Rep.* **2022**, *3*, 100256. https://doi.org/10.1016/j.jtocrr.2021.100256.
15. Ng, T.L.; Yu, H.; Smith, D.E.; Boyle, T.A.; York, E.R.; Leedy, S.; Gao, D.; Aisner, D.L.; Van Bokhoven, A.; Heasley, L.E.; et al. Preselection of Lung Cancer Cases Using FGFR1 mRNA and Gene Copy Number for Treatment With Ponatinib. *Clin. Lung Cancer* **2019**, *20*, e39–e51. https://doi.org/10.1016/j.cllc.2018.09.001.
16. Melichar, B.; Adenis, A.; Lockhart, A.C.; Bennouna, J.; Dees, E.C.; Kayaleh, O.; Obermannova, R.; DeMichele, A.; Zatloukal, P.; Zhang, B.; et al. Safety and activity of alisertib, an investigational aurora kinase A inhibitor, in patients with breast cancer, small-cell lung cancer, non-small-cell lung cancer, head and neck squamous-cell carcinoma, and gastro-oesophageal adenocarcinoma: A five-arm phase 2 study. *Lancet Oncol.* **2015**, *16*, 395–405. https://doi.org/10.1016/s1470-2045(15)70051-3.
17. Wang, C.Y.; Lee, M.H.; Kao, Y.R.; Hsiao, S.H.; Hong, S.Y.; Wu, C.W. Alisertib inhibits migration and invasion of EGFR-TKI resistant cells by partially reversing the epithelial-mesenchymal transition. *Biochim. Biophys. Acta Mol. Cell Res.* **2021**, *1868*, 119016. https://doi.org/10.1016/j.bbamcr.2021.119016.
18. Massabeau, C.; Khalifa, J.; Filleron, T.; Modesto, A.; Bigay-Gamé, L.; Plat, G.; Dierickx, L.; Aziza, R.; Rouquette, I.; Gomez-Roca, C.; et al. Continuous Infusion of Cilengitide Plus Chemoradiotherapy for Patients With Stage III Non-Small-cell Lung Cancer: A Phase I Study. *Clin. Lung Cancer* **2018**, *19*, e277–e285. https://doi.org/10.1016/j.cllc.2017.11.002.
19. Li, Y.; Fang, M.; Xu, Z.; Li, X. Tetrathiomolybdate as an old drug in a new use: As a chemotherapeutic sensitizer for non-small cell lung cancer. *J. Inorg. Biochem.* **2022**, *233*, 111865. https://doi.org/10.1016/j.jinorgbio.2022.111865.
20. Vokes, E.E.; Lyss, A.P.; Herndon, J.E., 2nd; Cooper, B.; Perry, M.C.; Vinciguerra, V.; Mason-Coughlin, K.; Green, M.R. Intravenous 6-thioguanine or cisplatin, fluorouracil and leucovorin for advanced non-small cell lung cancer: A randomized phase II study of the cancer and leukemia group B. *Ann. Oncol.* **1992**, *3*, 727–732. https://doi.org/10.1093/oxfordjournals.annonc.a058328.
21. Gao, H.; Song, Y.; Ma, J.; Zhai, J.; Zhang, Y.; Qu, X. Untargeted metabolomics analysis of omeprazole-enhanced chemosensitivity to cisplatin in mice with non-small cell lung cancer. *Chem. Biol. Interact.* **2022**, *360*, 109933. https://doi.org/10.1016/j.cbi.2022.109933.
22. Klinge, C.M. Inhibition of non-small-cell lung cancer growth by combined fulvestrant and vandetanib. *Future Oncol.* **2012**, *8*, 529–533. https://doi.org/10.2217/fon.12.42.
23. Cheng, Y.; Li, N.; Eapen, A.; Parajuli, R.; Mehta, R. Somatic BRCA2 Mutation-Positive Concurrent Accessory Male Breast Cancer (BC) and Non-Small Cell Lung Cancer (NSCLC): Excellent Efficacy of Palbociclib, Fulvestrant and Leuprolide in Platinum-Exposed and Endocrine-Refractory BC Associated with Cyclin D1 and FGFR1 Amplification and of Carboplatin, Paclitaxel and Radiation in NSCLC. *Case Rep. Oncol.* **2019**, *12*, 494–499. https://doi.org/10.1159/000501304.
24. Kochetova, O.V.; Avzaletdinova, D.S.; Korytina, G.F.; Morugova, T.V.; Mustafina, O.E. The association between eating behavior and polymorphisms in GRIN2B, GRIK3, GRIA1 and GRIN1 genes in people with type 2 diabetes mellitus. *Mol. Biol. Rep.* **2020**, *47*, 2035–2046. https://doi.org/10.1007/s11033-020-05304-x.
25. Gu, L.; Huang, J.; Li, J.; Huang, S.; Li, M.; Gong, L.; Li, T.; Su, L. Association of CALM1 rs3179089 Polymorphism with Ischemic Stroke in Chinese Han Population. *Neuromol. Med.* **2018**, *20*, 271–279. https://doi.org/10.1007/s12017-018-8492-z.
26. Jing, H.; Liu, L.; Jia, Y.; Yao, H.; Ma, F. Overexpression of the long non-coding RNA Oprm1 alleviates apoptosis from cerebral ischemia-reperfusion injury through the Oprm1/miR-155/GATA3 axis. *Artif. Cells Nanomed. Biotechnol.* **2019**, *47*, 2431–2439. https://doi.org/10.1080/21691401.2019.1626408.
27. Esteban, G.; Bolea, I.; Sun, P.; Solé, M.; Samadi, A.; Marco-Contelles, J.; Unzeta, M. A therapeutic approach to cerebrovascular diseases based on indole substituted hydrazides and hydrazines able to interact with human vascular adhesion protein-1, monoamine oxidases (A and B), AChE and BuChE. *J. Neural Transm. (Vienna)* **2013**, *120*, 911–918. https://doi.org/10.1007/s00702-012-0949-x.

28. Zhao, J.; Forsberg, C.W.; Goldberg, J.; Smith, N.L.; Vaccarino, V. MAOA promoter methylation and susceptibility to carotid atherosclerosis: Role of familial factors in a monozygotic twin sample. *BMC Med. Genet.* **2012**, *13*, 100. https://doi.org/10.1186/1471-2350-13-100.
29. Choi-Kwon, S.; Han, K.; Cho, K.H.; Choi, S.; Suh, M.; Nah, H.W.; Kim, J.S. Factors associated with post-stroke anger proneness in ischaemic stroke patients. *Eur. J. Neurol.* **2013**, *20*, 1305–1310. https://doi.org/10.1111/ene.12199.
30. Dai, S.; Ma, W.; Qin, G.; Wang, L.; Wang, E. Influence of ADRB1, ADRB2, and COMT Genetic Polymorphisms on Postoperative Outcomes of Patients Undergoing Cardiac Valve Surgery. *Clin. Ther.* **2021**, *43*, 349–359.e342. https://doi.org/10.1016/j.clinthera.2020.12.010.
31. Kim, B.R.; Kim, H.Y.; Chun, Y.I.; Yun, Y.M.; Kim, H.; Choi, D.H.; Lee, J. Association between genetic variation in the dopamine system and motor recovery after stroke. *Restor. Neurol. Neurosci.* **2016**, *34*, 925–934. https://doi.org/10.3233/rnn-160667.
32. Buga, A.M.; Ciobanu, O.; Bădescu, G.M.; Bogdan, C.; Weston, R.; Slevin, M.; Di Napoli, M.; Popa-Wagner, A. Up-regulation of serotonin receptor 2B mRNA and protein in the peri-infarcted area of aged rats and stroke patients. *Oncotarget* **2016**, *7*, 17415–17430. https://doi.org/10.18632/oncotarget.8277.
33. Martos, R.; Márton, I. Correlations between dental-oral infections and cardiovascular disease. *Fogorv. Sz.* **2008**, *101*, 101–105.
34. Nitsa, A.; Toutouza, M.; Machairas, N.; Mariolis, A.; Philippou, A.; Koutsilieris, M. Vitamin D in Cardiovascular Disease. *In Vivo* **2018**, *32*, 977–981. https://doi.org/10.21873/invivo.11338.
35. Kim, J.H.; Almuwaqqat, Z.; Hammadah, M.; Liu, C.; Ko, Y.A.; Lima, B.; Sullivan, S.; Alkhoder, A.; Abdulbaki, R.; Ward, L.; et al. Peripheral Vasoconstriction During Mental Stress and Adverse Cardiovascular Outcomes in Patients With Coronary Artery Disease. *Circ. Res.* **2019**, *125*, 874–883. https://doi.org/10.1161/circresaha.119.315005.
36. Amaro, C.A.; González-Cortazar, M.; Herrera-Ruiz, M.; Román-Ramos, R.; Aguilar-Santamaría, L.; Tortoriello, J.; Jiménez-Ferrer, E. Hypoglycemic and hypotensive activity of a root extract of Smilax aristolochiifolia, standardized on N-trans-feruloyl-tyramine. *Molecules* **2014**, *19*, 11366–11384. https://doi.org/10.3390/molecules190811366.
37. Katrak, P.H.; Cole, A.M.; Poulos, C.J.; McCauley, J.C. Objective assessment of spasticity, strength, and function with early exhibition of dantrolene sodium after cerebrovascular accident: A randomized double-blind study. *Arch. Phys. Med. Rehabil.* **1992**, *73*, 4–9.
38. Carpiniello, B.; Pinna, F.; Tuveri, R. Delusional infestation in a patient with renal failure, metabolic syndrome, and chronic cerebrovascular disease treated with aripiprazole: A case report. *Case Rep. Med.* **2011**, *2011*, 103652. https://doi.org/10.1155/2011/103652.
39. Frishman, W.H.; Hotchkiss, H. Selective and nonselective dopamine receptor agonists: An innovative approach to cardiovascular disease treatment. *Am. Heart J.* **1996**, *132*, 861–870. https://doi.org/10.1016/s0002-8703(96)90323-3.
