# Supplementary material for: DDCM: A Computational Strategy for Drug Repositioning Based on Support-Vector Regression Algorithm
Source: Int J Mol Sci. 2024 May 12;25(10):5267. doi: 10.3390/ijms25105267 (PMC11121335; doi:10.3390/ijms25105267)
Supplement: Supplementary file 1 [file ijms-25-05267-s001.zip › File S2.pdf]

Table S5. potential therapeutic drugs for neoplasms, Table S6. potential therapeutic drugs for vascular diseases, and Table S7. major enrichment situations of genetic subgroups for potential therapeutic drug targets in NSCLC.

Table S5. Potential therapeutic drugs for neoplasms

**Disease**	**Potential therapeutic drug (DrugBank ID)**
adrenal gland cancer	DB04655/DB09053/DB11617/DB04944/DB00421/DB06769/DB00446/DB05260/DB00753/DB11752/DB11467/DB13811/DB08804/DB04571/DB00480/DB09199/DB09054/DB01073/DB08882/DB00925/DB00277/DB01701/DB06810
adrenocortical carcinoma	DB01394/DB01179/DB00196/DB01217/DB00753/DB00213/DB09053/DB06448/DB01073/DB01138/DB01373/DB11890/DB11617/DB00448/DB09350/DB00446/DB05220/DB06769/DB04942/DB00727/DB11919/DB06641
bile duct cancer	DB01033/DB01254/DB11648/DB13014/DB01138/DB12802/DB00681/DB00477/DB06641/DB00254/DB01211/DB01700/DB05088/DB00338/DB06825/DB00302
biliary tract cancer	DB06809/DB05220/DB08889/DB02424/DB06810/DB00304/DB00443/DB09053/DB00590/DB02901/DB04348/DB15304/DB00753/DB06444/DB01357/DB01162/DB04655/DB00137/DB01177/DB11919/DB06448/DB01095/DB11251/DB00435/DB00477
bone cancer	DB09350/DB06810/DB11890/DB00815/DB00443/DB08804/DB00213/DB14545/DB12802/DB00884/DB15334/DB04865/DB00352/DB00137/DB09053/DB09199/DB00304/DB00254/DB01138/DB13911/DB00477/DB00480/DB00338
brain cancer	DB11919/DB01394/DB06810/DB13014/DB01138/DB00352/DB09350/DB01033/DB04106/DB01162/DB00137/DB00254/DB13811/DB01177/DB00302/DB04944/DB01179/DB00448/DB00446/DB12874/DB11890/DB11752/DB00338/DB05220/DB09054
bronchiolo-alveolar adenocarcinoma	DB00171/DB04348/DB06810/DB01179/DB02901/DB13911/DB00443/DB00134/DB00815/DB04942/DB01394/DB11919/DB11251/DB00571/DB01095
central nervous system cancer	DB00352/DB04942/DB01179/DB01394/DB04106/DB11890/DB13014/DB06810/DB04655/DB11752/DB00254/DB09053/DB09054/DB04944/DB06176/DB00281/DB15334/DB04868/DB00446/DB01017/DB01211/DB06732/DB11919/DB01138
cervical cancer	DB01357/DB01138/DB01162/DB12257/DB04942/DB06810/DB13014/DB00947/DB01394/DB05260/DB11919/DB05220/DB00352/DB15304/DB00448/DB00137/DB09350/DB13811/DB02424
colon cancer	DB00681/DB14322/DB01033/DB00352/DB11648/DB01073/DB00281/DB01254/DB06728/DB00470/DB11752/DB00724/DB11251/DB01698/DB01394/DB00583/DB11919/DB14545
connective tissue benign neoplasm	DB13014/DB05220/DB08804/DB14545/DB00213/DB01211/DB01138/DB02375/DB00196/DB00446/DB00448/DB01073/DB007

Disease	Potential therapeutic drug (DrugBank ID)
	53/DB09199/DB11251/DB00137/DB00742/DB09350/DB00947/DB01177/DB01179/DB00571/DB00470
endometrial cancer	DB00134/DB00947/DB01217/DB01033/DB08804/DB04571/DB04942/DB00724/DB11919/DB00421/DB11890/DB00717/DB01138/DB06412/DB01254/DB05260/DB00594/DB06732/DB00470/DB00254/DB04258/DB00352
endometrial carcinoma	DB00717/DB00304/DB01217/DB00213/DB01073/DB00014/DB13014/DB00137/DB00815/DB04106/DB05220/DB11890/DB05088/DB01138/DB12948/DB00947/DB00571/DB00421/DB01708/DB06641/DB06176/DB02901/DB01420/DB09350/DB01698/DB00254/DB00338
esophageal cancer	DB01162/DB05220/DB11919/DB00421/DB01177/DB00590/DB01394/DB06174/DB09054/DB00213/DB13811/DB01099/DB09350/DB00443/DB01217/DB00681/DB06176/DB04571/DB11648/DB01073/DB06641/DB01033/DB00448
familial retinoblastoma	DB01179/DB01394/DB00681/DB11919/DB01254/DB04942/DB04258/DB02901/DB13014/DB01073/DB01759/DB05220/DB05260/DB00815/DB00352/DB01136/DB00477/DB06179/DB09053/DB04944/DB00014/DB01708/DB06732/DB06757
female reproductive organ cancer	DB09350/DB00137/DB05220/DB00213/DB06810/DB00448/DB14545/DB00446/DB00338/DB12948/DB04865/DB00435/DB00753/DB02375/DB00947/DB13014/DB08804/DB01136/DB00727/DB13911/DB00443/DB01357/DB05088/DB01138/DB11890
gallbladder cancer	
growth hormone secreting pituitary adenoma	DB00050/DB01217/DB00421/DB01700/DB00480/DB05088/DB01420/DB06641/DB00446/DB06810/DB09054/DB00422/DB11175/DB04106/DB01357/DB06536/DB00947/DB08868/DB04868/DB08889/DB00742/DB00571
head and neck cancer	DB01162/DB04258/DB01177/DB09053/DB01394/DB04942/DB11890/DB13811/DB01099/DB00352/DB11919/DB09350/DB00724/DB00213/DB11752/DB01254/DB12948/DB04815/DB13911/DB11648/DB11175/DB00681/DB01033/DB00947
inflammatory breast carcinoma	
intestinal cancer	DB00681/DB01080/DB01033/DB04258/DB11467/DB04942/DB00470/DB01254/DB00815/DB04944/DB11648/DB00443/DB01100/DB00947/DB00446/DB02901/DB05260/DB01099/DB01759/DB09350/DB01211/DB06732
invasive lobular carcinoma	DB00694/DB01033/DB14066/DB13911/DB06174/DB02901/DB08882/DB00938/DB01006/DB12881/DB01217/DB00909/DB00863
kidney cancer	DB01357/DB01162/DB08901/DB01033/DB11919/DB00213/DB01394/DB00448/DB01177/DB01138/DB05258/DB09350/DB138

Disease	Potential therapeutic drug (DrugBank ID)
	11/DB00435/DB00863/DB06732/DB00947/DB05260/DB01100/DB06810/DB00338/DB00137
large intestine cancer	DB00134/DB00352/DB01254/DB01394/DB06810/DB00724/DB00681/DB11251/DB01033/DB11919/DB01177/DB01100/DB01162/DB13811/DB04258/DB02424/DB00470/DB00137/DB04944/DB11648/DB15304/DB00863
larynx cancer	DB01033/DB00947/DB00681/DB00909/DB03057/DB01136/DB01073/DB06728/DB01162/DB02901/DB04815/DB00213/DB00884/DB09053/DB00446/DB00421/DB06176/DB13911/DB11890/DB00717/DB00753/DB01211/DB00904/DB04348/DB09350/DB00448/DB00594/DB06448/DB01017/DB00742
lung benign neoplasm	DB00470/DB01033/DB00352/DB00137/DB01708/DB12874/DB00947/DB01162/DB01254/DB11648/DB13014/DB13964/DB00863/DB06757/DB00815/DB09350
lung small cell carcinoma	DB01394/DB05220/DB13014/DB01179/DB13811/DB06810/DB01162/DB09054/DB00448/DB11919/DB04865/DB00352/DB00137/DB12257/DB01138/DB04571/DB01373/DB02424/DB00863/DB00014
male breast cancer	DB00947/DB00254/DB00443/DB00717/DB05258/DB06732/DB01217/DB00134/DB01138/DB00470/DB02901/DB00014/DB08804/DB11890/DB09350/DB08868/DB05220/DB01698/DB01373/DB04942/DB00304/DB00196
malignant mesothelioma	DB00884/DB01073/DB08901/DB04942/DB00909/DB14322/DB00352/DB01100/DB11135/DB11616/DB00877/DB06824
myasthenia gravis	DB01033/DB06412/DB11648/DB11752/DB04571/DB00281/DB00681/DB05260/DB00480/DB00352/DB05258/DB00421/DB01211/DB01254/DB00590/DB01080/DB01177/DB01373/DB06179/DB00772/DB00431/DB13424
nasopharynx carcinoma	DB00134/DB01073/DB06179/DB04571/DB06176/DB00815/DB13014/DB11467/DB04106/DB01708/DB05088/DB02901/DB00884/DB01177/DB06732/DB05260/DB04868/DB00727/DB12874/DB06444/DB00742/DB00947/DB01254/DB01759
nephroblastoma	DB01394/DB06810/DB01162/DB00137/DB04942/DB05220/DB01138/DB01179/DB00863/DB11919/DB00571/DB13811/DB13014/DB00014/DB09350/DB14545/DB05260/DB01073/DB11251/DB15304/DB00815
non-small cell lung carcinoma	DB02424/DB01394/DB01177/DB13811/DB06810/DB01254/DB06176/DB00590/DB11919/DB00448/DB01099/DB04815/DB08901/DB05220/DB04944/DB11890/DB06444/DB11648/DB01179/DB05088/DB01162/DB00352/DB00338/DB09350/DB00947
ovarian cancer	DB01394/DB13014/DB01177/DB06176/DB09054/DB01138/DB08901/DB04942/DB01254/DB02424/DB01162/DB06810/DB13811/DB01357/DB00352/DB06174/DB00717/DB00421/DB11890/

Disease	Potential therapeutic drug (DrugBank ID)
	DB01017/DB05220/DB00947/DB06641
pancreatic cancer	DB00470/DB04942/DB04106/DB01177/DB01073/DB02424/DB01179/DB11919/DB06810/DB04944/DB00196/DB00681/DB06757/DB02375/DB00724/DB06176/DB01254/DB00435/DB04258/DB08804/DB09350
pancreatic carcinoma	DB00884/DB00571/DB04258/DB00855/DB00553/DB01073/DB06536/DB11919/DB04655/DB06176/DB09054/DB08804/DB13911/DB01356/DB00780/DB00724/DB01095/DB14322
pancreatic ductal carcinoma	DB06444/DB00304/DB00717/DB01254/DB02424/DB05088/DB06448/DB00134/DB01177/DB00724/DB01357/DB06810/DB00863/DB06412/DB01211/DB01373/DB05260/DB08901/DB00947/DB00448/DB01708/DB15304/DB12948/DB01073/DB09054
parathyroid carcinoma	DB00904/DB09054/DB01073/DB06769/DB00277/DB00437/DB00242/DB01033/DB00352/DB09053/DB00938
penile cancer	DB13014/DB06412/DB01138/DB00435/DB09054/DB01179/DB06174/DB08868/DB00014/DB03068/DB15334/DB05220/DB13911/DB06176/DB03057/DB00304/DB08889/DB04571/DB12802/DB04655/DB13964/DB14322/DB01017
pituitary cancer	DB01357/DB00947/DB09350/DB00422/DB01100/DB00050/DB00909/DB08804/DB00443/DB08889/DB01698/DB00338/DB00014/DB00421/DB08882/DB06810/DB01708/DB01217/DB06732/DB00571/DB01420/DB15334/DB08868/DB00863/DB00171/DB06809/DB00717
pleural cancer	DB00717/DB00254/DB01373/DB05258/DB00304/DB00137/DB08868/DB11890/DB08804/DB11752/DB06728/DB01138/DB00470/DB00480/DB01217/DB01698/DB00571/DB06809/DB11467/DB13014/DB00884/DB08901/DB01177/DB00422/DB11617
prolactinoma	DB00422/DB00947/DB09350/DB01217/DB06767/DB00050/DB01100/DB01420/DB01357/DB01700/DB06810/DB00338/DB11175/DB01708/DB00480/DB06732/DB00477/DB00909/DB01080/DB01698/DB04868/DB08901
rectal neoplasm	DB01033/DB01254/DB00681/DB11648/DB04571/DB00421/DB06412/DB01394/DB01211/DB00422/DB00717/DB00909/DB01099/DB08889/DB09054/DB06174/DB14322/DB04868/DB05258/DB00815/DB01138/DB06809/DB11251
renal cell carcinoma	DB00134/DB01394/DB06810/DB11919/DB05220/DB06809/DB04944/DB01162/DB01177/DB01254/DB04655/DB01138/DB01100/DB04942/DB02424/DB11890/DB06412/DB00137/DB05260
skin benign neoplasm	DB04942/DB14545/DB00470/DB00281/DB02375/DB09350/DB11251/DB00815/DB06810/DB01073/DB02901/DB00213/DB04106/DB08868/DB00352/DB13014/DB01708/DB00904/DB00681/DB01179/DB00742/DB03068/DB06757
skin melanoma	DB00553/DB00855/DB04655/DB04571/DB00806/DB00242/DB

Disease	Potential therapeutic drug (DrugBank ID)
	00307/DB02546/DB00188/DB06176/DB08868/DB01268/DB01590/DB05258/DB08901/DB03057/DB00744/DB04868/DB01373/DB01254/DB00877/DB01080
stomach cancer	DB00134/DB01162/DB09054/DB01254/DB06448/DB00863/DB09053/DB00753/DB11648/DB01017/DB11251/DB00281/DB08804/DB08901/DB00448/DB00470/DB13964/DB01099/DB04258/DB14322/DB01073
supratentorial cancer	DB01394/DB13014/DB01179/DB00681/DB04942/DB09054/DB06728/DB09053/DB00254/DB01177/DB11467/DB06810/DB01708/DB03057/DB00421/DB01073/DB06769/DB00742/DB12948/DB11919/DB09350/DB00724/DB00338/DB01138/DB06179/DB00014/DB01759
testicular cancer	DB01394/DB06810/DB05220/DB01179/DB01162/DB13811/DB09350/DB01177/DB04942/DB11919/DB00863/DB01136/DB00947/DB01138/DB00571/DB05260/DB00137/DB01100/DB15304/DB00448/DB01373/DB04865/DB02424/DB11251
thymoma	DB00050/DB04106/DB11467/DB05088/DB09054/DB01217/DB00446/DB06641/DB00590/DB08804/DB06810/DB00480/DB01420/DB01136/DB04868/DB01700/DB00196/DB00947/DB06536/DB01162/DB00352/DB15304/DB12802/DB01698
thymus cancer	DB00531/DB00290/DB00570/DB00755/DB00541/DB00724/DB12948/DB14545/DB06810/DB06536/DB11648/DB14066/DB00681/DB08868/DB04865/DB11617/DB06174/DB12802/DB04690/DB03057/DB01033/DB01254/DB01211/DB04655/DB13323/DB04571/DB13014/DB08901/DB01420
thyroid cancer	DB00594/DB04942/DB06174/DB01394/DB00352/DB00681/DB11617/DB00724/DB01179/DB04944/DB04865/DB11890/DB06176/DB08901/DB06448/DB00213/DB12802/DB00470/DB06728/DB04258/DB00590/DB04868
thyroid medullary carcinoma	DB01254/DB00909/DB02424/DB03068/DB08804/DB00724/DB11648/DB00947/DB00352/DB12948/DB08901/DB01700/DB01073/DB00884/DB01373/DB09053/DB04942/DB00196/DB06176/DB11752/DB01017/DB12874/DB00134/DB01217/DB00681/DB00727/DB11919
tongue cancer	DB04942/DB00338/DB00352/DB01254/DB09350/DB11467/DB00947/DB00815/DB01073/DB06176/DB01759/DB02901/DB08868/DB00909/DB01708/DB05260/DB04258/DB00443/DB06179/DB06732/DB04868/DB06769
urinary bladder cancer	DB05220/DB09350/DB13014/DB01394/DB01177/DB11919/DB01179/DB01138/DB01033/DB00681/DB08804/DB06810/DB08882/DB11890/DB00947/DB01698/DB01254/DB00435/DB05260/DB00338/DB11251
urinary system benign	DB06728/DB00681/DB00213/DB00137/DB09053/DB05260/DB

Disease	Potential therapeutic drug (DrugBank ID)
neoplasm	00724/DB12948/DB09199/DB11467/DB04258/DB11919/DB01759/DB00884/DB00254/DB01073/DB13014/DB01179/DB01136/DB05220/DB03057
uterine cance	DB00947/DB01100/DB01073/DB11890/DB01217/DB00717/DB09350/DB11251/DB00904/DB01254/DB00050/DB09199/DB04815/DB13014/DB00884/DB01138/DB00470/DB04571/DB02424
uveal melanoma	DB04868/DB08882/DB09350/DB08901/DB00753/DB05260/DB04942/DB00134/DB00724/DB00435/DB01254/DB11617/DB01080/DB13964/DB06179/DB01017/DB01100/DB06174/DB00338/DB00050/DB00422/DB00470/DB11752/DB12802/DB06448

Table S6. Potential therapeutic drugs for vascular diseases

Disease	Potential therapeutic drug (DrugBank ID)
abdominal aortic aneurysm	DB01219/DB00332/DB00523/DB14574/DB00104/DB11673/DB06212/DB00796/DB13243/DB14015/DB00897/DB00761/DB04557/DB09153/DB01174/DB00143/DB07352/DB06155
angioedema	DB09477/DB00989/DB00214/DB11742/DB04953/DB00567/DB00243/DB00927/DB00415/DB01611/DB05549/DB00261/DB00694/DB00557/DB00983/DB12693/DB00313
anterior ischemic optic neuropathy	DB00477/DB00332/DB00261/DB14015/DB00796/DB00934/DB00845/DB13504/DB13243/DB11326/DB00431/DB00545/DB02315/DB01149/DB13323/DB00653/DB00284/DB13723/DB11152
aortic aneurysm	DB00204/DB00143/DB00557/DB00254/DB01074/DB13323/DB01288/DB00796/DB05549/DB04557/DB01015/DB00934/DB00285/DB01413/DB12870/DB02806/DB09086
aortic disease	DB09313/DB01268/DB05088/DB01219/DB06155/DB00254/DB01171/DB13863/DB09153/DB11829/DB00313/DB13108/DB00927/DB11326/DB01174/DB13781/DB03843/DB02315
arteriosclerosis	DB00380/DB00261/DB04861/DB00543/DB13781/DB01053/DB04104/DB00687/DB01235/DB01184/DB00910/DB04953/DB00332/DB12422/DB01233/DB00431/DB01288/DB09086/DB00746/DB01159/DB06155/DB13723
ataxia telangiectasia	DB01191/DB01429/DB01611/DB00746/DB00313/DB05382/DB00248/DB01174/DB00579/DB14574/DB01195/DB01356/DB00908/DB01064/DB06810/DB00325/DB00247/DB09152
atherosclerosis	DB08860/DB00502/DB04557/DB00910/DB00284/DB13323/DB08839/DB00694/DB12870/DB00988/DB06155/DB00431/DB00523/DB09153/DB09477/DB00761/DB00143/DB00796/DB00439/DB01954/DB00171/DB00752/DB11588/DB00845/DB08841
Behcet's disease	DB00681/DB01611/DB08809/DB12422/DB13110/DB11387/DB00313/DB01189/DB01233/DB05015/DB00431/DB09086/DB11673/DB00780/DB15584/DB01400/DB01015/DB01171/DB13682
brain infarction	DB00502/DB00574/DB01100/DB00243/DB00204/DB00910/DB

Disease	Potential therapeutic drug (DrugBank ID)
	00813/DB00794/DB01233/DB00279/DB12693/DB00780/DB00934/DB08839/DB00681/DB00527/DB13108
brain ischemia	DB02315/DB01028/DB00312/DB00214/DB05549/DB03054/DB06155/DB00988/DB09153/DB01149/DB00186/DB12422/DB00761/DB00425/DB00752/DB00143/DB01954/DB13863/DB00271/DB05057/DB11829
carotid artery disease	DB14015/DB00380/DB00574/DB01100/DB00746/DB05549/DB01233/DB13243/DB12662/DB00279/DB00934/DB13723/DB00261/DB13504/DB00845/DB00332/DB09072/DB00614/DB13863/DB05063/DB05088/DB08842/DB00207
carotid artery thrombosis	DB00694/DB05549/DB01233/DB09069/DB00248/DB00261/DB00313/DB00104/DB01100/DB00434/DB14015/DB00574/DB00752/DB11387/DB01268/DB00850/DB13723/DB12693/DB00934/DB00816/DB00594/DB00279
carotid stenosis	DB12870/DB13110/DB01233/DB00261/DB00694/DB11673/DB11829/DB01204/DB06176/DB11588/DB01113/DB00871/DB00171/DB00988/DB00780/DB02021/DB06810/DB05382/DB00176
cerebral infarction	DB00313/DB00816/DB01268/DB00752/DB00988/DB06212/DB00261/DB09069/DB00243/DB01171/DB00780/DB00694/DB06589/DB14015/DB00176/DB00104/DB03843/DB00285
cerebrovascular disease	DB01191/DB00579/DB00261/DB01954/DB13108/DB08841/DB13243/DB00104/DB13358/DB01233/DB01244/DB01219/DB01238/DB08839/DB06690/DB00988/DB00850/DB00752/DB01074/DB01288/DB00285/DB11152/DB01268/DB02806/DB01015
compartment syndrome	DB00502/DB08839/DB12881/DB00439/DB00752/DB00574/DB01140/DB09152/DB00285/DB00312/DB12693/DB00527/DB01149/DB00780/DB00143/DB00271/DB00927/DB00358
coronary aneurysm	DB05223/DB00285/DB09313/DB12422/DB00988/DB00365/DB00502/DB01288/DB11152/DB00271/DB05063/DB01297/DB00816/DB01611/DB00207/DB00527/DB00358/DB00417/DB12622/DB02806/DB04557/DB01238
coronary artery disease	DB02315/DB01288/DB00614/DB11588/DB04855/DB04557/DB00796/DB01113/DB08860/DB00468/DB00761/DB00871/DB00523/DB00332/DB00243/DB00380
coronary artery vasospasm	DB00813/DB01429/DB02315/DB00761/DB01015/DB00927/DB00104/DB00816/DB09153/DB00988/DB13108/DB00796/DB00653/DB13323/DB01233/DB00171/DB12693/DB08839/DB08842/DB09069
coronary restenosis	DB04855/DB00752/DB00312/DB00176/DB06212/DB00910/DB00261/DB08841/DB00680/DB09313/DB00780/DB00988/DB00431/DB12422/DB00802/DB00415
coronary stenosis	DB00332/DB00794/DB05549/DB01182/DB09072/DB02315/DB11326/DB00176/DB01113/DB14015/DB08842/DB00813/DB012

Disease	Potential therapeutic drug (DrugBank ID)
	88/DB01142/DB00934/DB00346/DB00574/DB01954/DB00285/DB11387/DB01143
coronary thrombosis	DB09313/DB00694/DB00261/DB02806/DB14015/DB01143/DB01954/DB00285/DB00574/DB08839/DB00152/DB00104/DB03843/DB00284/DB00365/DB00752/DB00434/DB01171/DB09069/DB04557
diabetic angiopathy	DB12881/DB00910/DB11829/DB11588/DB12422/DB00243/DB04557/DB00358/DB01288/DB00529/DB00521/DB00261/DB01182/DB09212/DB00813/DB06176/DB01174/DB01189
diabetic retinopathy	DB01191/DB02021/DB00988/DB00574/DB00143/DB08839/DB09212/DB00248/DB00687/DB01235/DB00204/DB13323/DB01140/DB00434
erythromelalgia	DB01219/DB00248/DB01182/DB04953/DB00434/DB01426/DB00332/DB00104/DB00796/DB00261/DB12693/DB00816/DB11742/DB00850/DB00897/DB01244/DB00285/DB09086/DB01429/DB01233/DB01100/DB11673
essential hypertension	DB00312/DB00104/DB00813/DB04855/DB00521/DB09152/DB00417/DB00171/DB01113/DB00242/DB00674/DB00271/DB09153/DB03843/DB00679/DB00468/DB00598/DB00829/DB00883/DB00572/DB00501
Fabry disease	DB08839/DB00943/DB01429/DB00500/DB01195/DB13110/DB01035/DB11136/DB11152/DB00276/DB00802/DB08809/DB00284/DB00332/DB05382/DB00297/DB00244
Henoch-Schoenlein purpura	DB00833/DB01219/DB00313/DB00567/DB01140/DB00910/DB00738/DB00243/DB11387/DB00574/DB09477/DB00261/DB00285/DB00543/DB00988/DB00529/DB00796/DB01015/DB01611/DB01238
hepatic vein thrombosis	DB00694/DB00313/DB00380/DB11447/DB01268/DB12422/DB01233/DB00527/DB00485/DB12881/DB06176/DB13243/DB00794/DB09313/DB14574/DB06155/DB06810/DB00614/DB00943/DB11387/DB14015/DB01182/DB05063
hereditary angioedema	DB05549/DB00527/DB00752/DB00439/DB09069/DB00594/DB05223/DB00521/DB13682/DB05015/DB00176/DB01400/DB00243/DB13723/DB01100
intermediate coronary syndrome	DB12881/DB00332/DB01244/DB01219/DB00285/DB13358/DB00358/DB00681/DB00313/DB00271/DB01370/DB00523/DB00152/DB01268/DB01140/DB04557/DB08842/
intracranial aneurysm	DB00816/DB00988/DB01219/DB05549/DB05383/DB00527/DB00752/DB04953/DB00261/DB06155/DB00796/DB00285/DB11387/DB01413/DB03843/DB13732/DB00439/DB00543/DB12693/DB06176/DB04557
intracranial thrombosis	DB09313/DB00502/DB00261/DB00271/DB00527/DB05549/DB13110/DB00988/DB00816/DB03843/DB00752/DB05223/DB118

Disease	Potential therapeutic drug (DrugBank ID)
	29/DB00802/DB01611/DB09072/DB08809/DB00279/DB00738/DB06176/DB09069
Kawasaki disease	DB01238/DB09313/DB01184/DB00485/DB00910/DB00358/DB00261/DB05015/DB00816/DB01233/DB00271/DB00365/DB00285/DB00523/DB01143/DB12622/DB05382/DB01288/DB06460/DB06558
malignant hypertension	DB09477/DB00214/DB01015/DB06589/DB09153/DB00653/DB00761/DB01189/DB01408/DB00313/DB00411/DB01297/DB08839/DB00813/DB00656/DB00431/DB13781/DB00107/DB13732/DB00694/DB01204/DB02315/DB01219
multiple myeloma	DB00694/DB11447/DB03843/DB09371/DB11616/DB00819/DB15584/DB00523/DB00276/DB00261/DB01268/DB00313/DB05015/DB06176/DB06810/DB05223/DB00614/DB04855/DB00738/DB13323/DB09313/DB00681/DB12422/DB09086
multiple system atrophy	DB00313/DB01357/DB09371/DB13919/DB00717/DB01050/DB11846/DB00695/DB00390/DB00328/DB03054/DB01240/DB09153/DB00802/DB01429/DB00276/DB01164/DB00393/DB00297/DB11387/DB06690/DB11447
myocardial infarction	DB01182/DB09477/DB00521/DB00694/DB00796/DB01288/DB12752/DB09153/DB00332/DB09069/DB02315/DB05057/DB12422/DB00313/DB01113/DB08841/DB00431/DB05549/DB01149/DB01233
peripheral vascular disease	DB00574/DB00694/DB00656/DB12693/DB00243/DB05549/DB01426/DB00434/DB09069/DB12752/DB00521/DB00557/DB00380/DB00897/DB00313/DB08839/DB00910/DB01233/DB01288/DB01171/DB01268/DB08826/DB00279/DB00207/DB14015/DB02315
pseudoxanthoma elasticum	DB09086/DB09371/DB00143/DB00284/DB04557/DB00988/DB01400/DB00313/DB00431/DB11152/DB05549/DB00279/DB03054/DB04861/DB05382
pulmonary embolism	DB00502/DB00694/DB00313/DB05549/DB12693/DB00543/DB00527/DB13323/DB00176/DB02806/DB06148/DB00988/DB06176/DB00780/DB14574/DB08839/DB00285/DB09313/DB00518
pulmonary venoocclusive disease	DB11387/DB01174/DB00313/DB00500/DB00698/DB03054/DB01182/DB00794/DB11588/DB00279/DB13323/DB00523/DB04557/DB01370/DB00776/DB00795
renal hypertension	DB00214/DB00796/DB01297/DB01189/DB09477/DB01219/DB00833/DB09153/DB00813/DB00104/DB04335/DB01204/DB01268/DB11616/DB00313/DB08826/DB00529/DB01159/DB00910
renovascular hypertension	DB00313/DB09477/DB00796/DB00910/DB00485/DB01189/DB12870/DB00653/DB01268/DB04855/DB00574/DB00254/DB01233/DB00567/DB08826/DB09153
retinal vein occlusion	DB01357/DB00261/DB00816/DB00285/DB13504/DB01288/DB

Disease	Potential therapeutic drug (DrugBank ID)
	00614/DB00313/DB00850/DB01233/DB12881/DB00104/DB09313/DB00279/DB05549/DB01954/DB00284/DB00434/
transient cerebral ischemia	DB00176/DB01182/DB05549/DB02315/DB01297/DB00934/DB13863/DB08839/DB01288/DB01189/DB05382/DB09153/DB00816/DB13243/DB00285/DB12422/
vasculitis	DB01140/DB00816/DB00261/DB05015/DB00681/DB01015/DB01288/DB01053/DB01268/DB13323/DB03843/DB05223/DB01370/DB06810/DB00279/DB13732/DB01219/DB00752/DB02806/DB00567
Wegener's granulomatosis	DB01204/DB00313/DB13323/DB01268/DB01174/DB01238/DB00681/DB00276/DB05015/DB00261/DB06690/DB09153/DB00431/DB09371/DB00780/DB01611/DB01140/DB05382/DB00890

Table S7. Major enrichment situations of genetic subgroups for potential therapeutic drug targets in NSCLC

Group	Information	Information content
I	Target	ESR1/TNF/TOP2A/DNMT1/AHR
The same pathogenic and therapeutic mechanisms.	Drug	DB00947/DB06444/DB01177/DB01179/DB01099/DB00338
	GO	cAMP-mediated signaling/immune response
	KEGG	Chemical carcinogenesis - reactive oxygen species/Chemical carcinogenesis - receptor activation/Th17 cell differentiation
II	Target	TUBB/FGFR2/HDAC1/HDAC2/HDAC6/APP/MAPT
Same therapeutic function as known drugs.	Drug	DB01179/DB01394/DB08901/DB06176/DB05088/DB00448
	GO	epidermis development/epithelial cell apoptotic process/epithelial cell migration/epithelial cell proliferation/epithelial to mesenchymal transition/chromatin remodeling/Cellular response to internal and external stimuli/regulation of cell cycle
	KEGG	Cell cycle/MAPK signaling pathway/MicroRNAs in cancer/Viral carcinogenesis/Ras signaling pathway/Rap1 signaling pathway
III	Target	KDR/KIT/SRC/EPHA2/PDGFRA/TGFB1/HSP90AA1/FGFR4/CSK/FRK
Potential to the function of drugs that act on the causative mechanism of the disease.	Drug	DB01254/DB08901/DB02424/DB01162
	GO	cellular response to oxidative stress/cellular response to vascular endothelial growth factor stimulus/cellular response to vascular endothelial growth factor stimulus/epithelial cell proliferation/epithelium migration/ERK1 and ERK2 cascade
	KEGG	EGFR tyrosine kinase inhibitor resistance/MAPK signaling pathway/PI3K-Akt signaling pathway/Ras signaling pathway/Th17 cell differentiation

IV Unique functions of potential therapeutic drugs	Target	ABL1/KCNH2/FLT3/PDGFRB/ATP4A/PPAT/ABL2/KCNH6/ADRA1A/ADRA1B/KCNH7/ADRA1D/ABCC1/FYN/YES1/LCK/STAT5B/AURKA/MAPK14/HSP90B1/BTK/TUBA4A/HSP90AB1/FGFR1/FGFR3/RET/EPHB4/TEK/HDAC4/HSPA8/LYN/BCR/FGR/EPHA5/ZAK/NR4A3
	Drug	DB01254/DB08901/DB01162/DB00338/DB00448/DB00590/DB06176/DB05220/DB02424/DB01179
	GO	branching morphogenesis of an epithelial tube/cell-substrate adhesion/immune response-activating cell surface receptor signaling pathway/immune response-activating signal transduction/immune response-regulating cell surface receptor signaling pathway/negative regulation of MAPK cascade
	KEGG	Th17 cell differentiation/Ras signaling pathway/PI3K-Akt signaling pathway/NF-kappa B signaling pathway/MAPK signaling pathway/EGFR tyrosine kinase inhibitor resistance/Chemical carcinogenesis - receptor activation/Cell cycle
