# Supplementary material for: DDCM: A Computational Strategy for Drug Repositioning Based on Support-Vector Regression Algorithm
Source: Int J Mol Sci. 2024 May 12;25(10):5267. doi: 10.3390/ijms25105267 (PMC11121335; doi:10.3390/ijms25105267)
Supplement: Supplementary file 1 [file ijms-25-05267-s001.zip › File S4/File S4 caption.pdf]

1. Correlation score between drug–disease (C04.588). 2. Stability scores of drugs and their distribution box plots when predicting diseases of C04.588. 3. Adjusted cosine similarity of pathogenic genes for diseases of C04.588. 4. Adjusted cosine similarity of pathways for diseases of C04.588. 5. Adjusted cosine similarity of target genes of drugs screened when predicting potential therapeutic drugs for the disease of C04.588. 6. Adjusted cosine similarity of pathways of drugs screened when predicting potential therapeutic drugs for the disease of C04.588.
