# Supplementary figures and images for: DDCM: A Computational Strategy for Drug Repositioning Based on Support-Vector Regression Algorithm
Source: Int J Mol Sci. 2024 May 12;25(10):5267. doi: 10.3390/ijms25105267 (PMC11121335; doi:10.3390/ijms25105267)

# adrenal gland cancer\_stability analysis

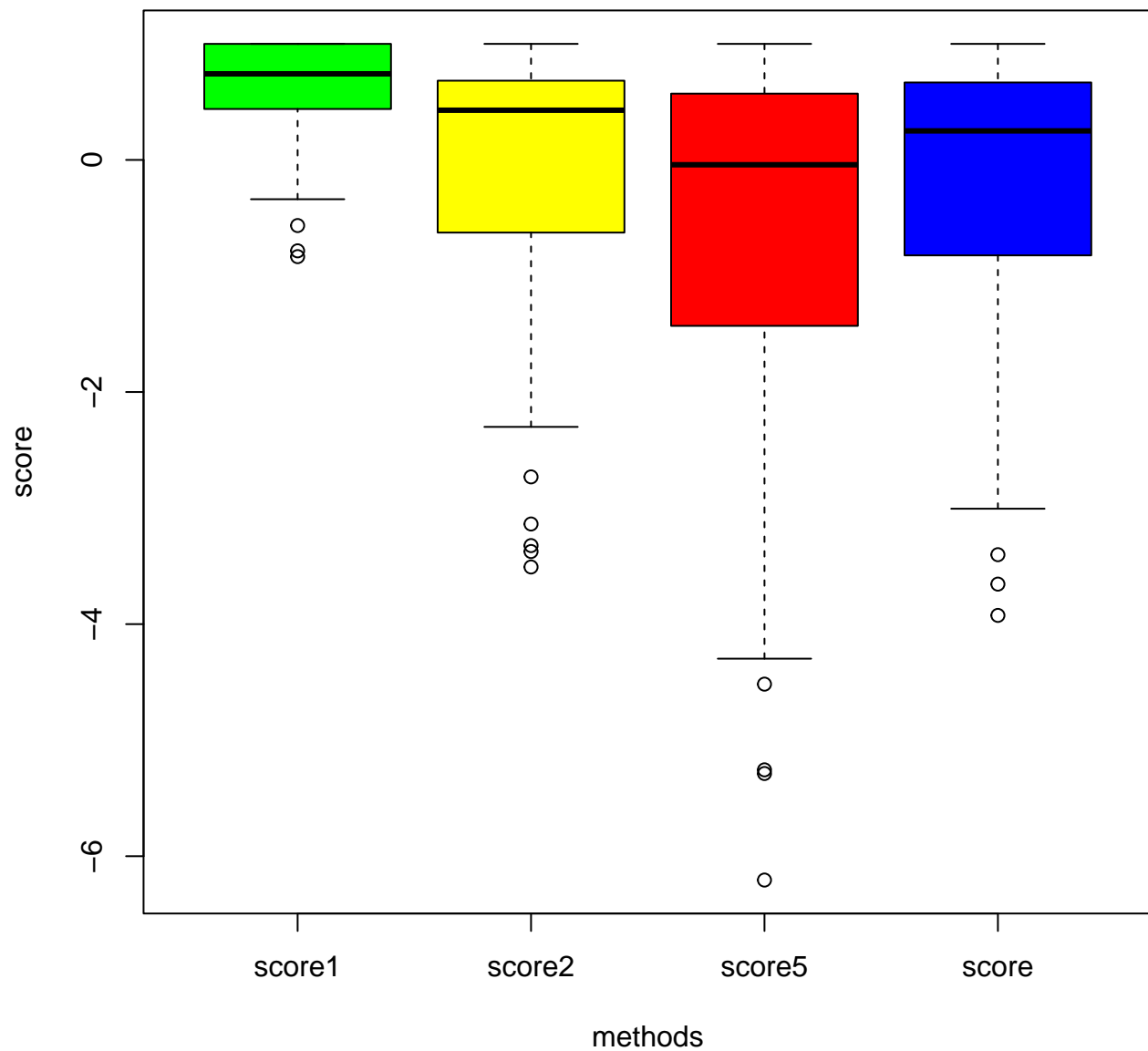

Supplement: Supplementary file 1 [file ijms-25-05267-s001.zip › File S4/2_stability_scores_boxplot/adrenal gland cancer_StabBoxplot.pdf]

# adrenocortical carcinoma\_stability analysis

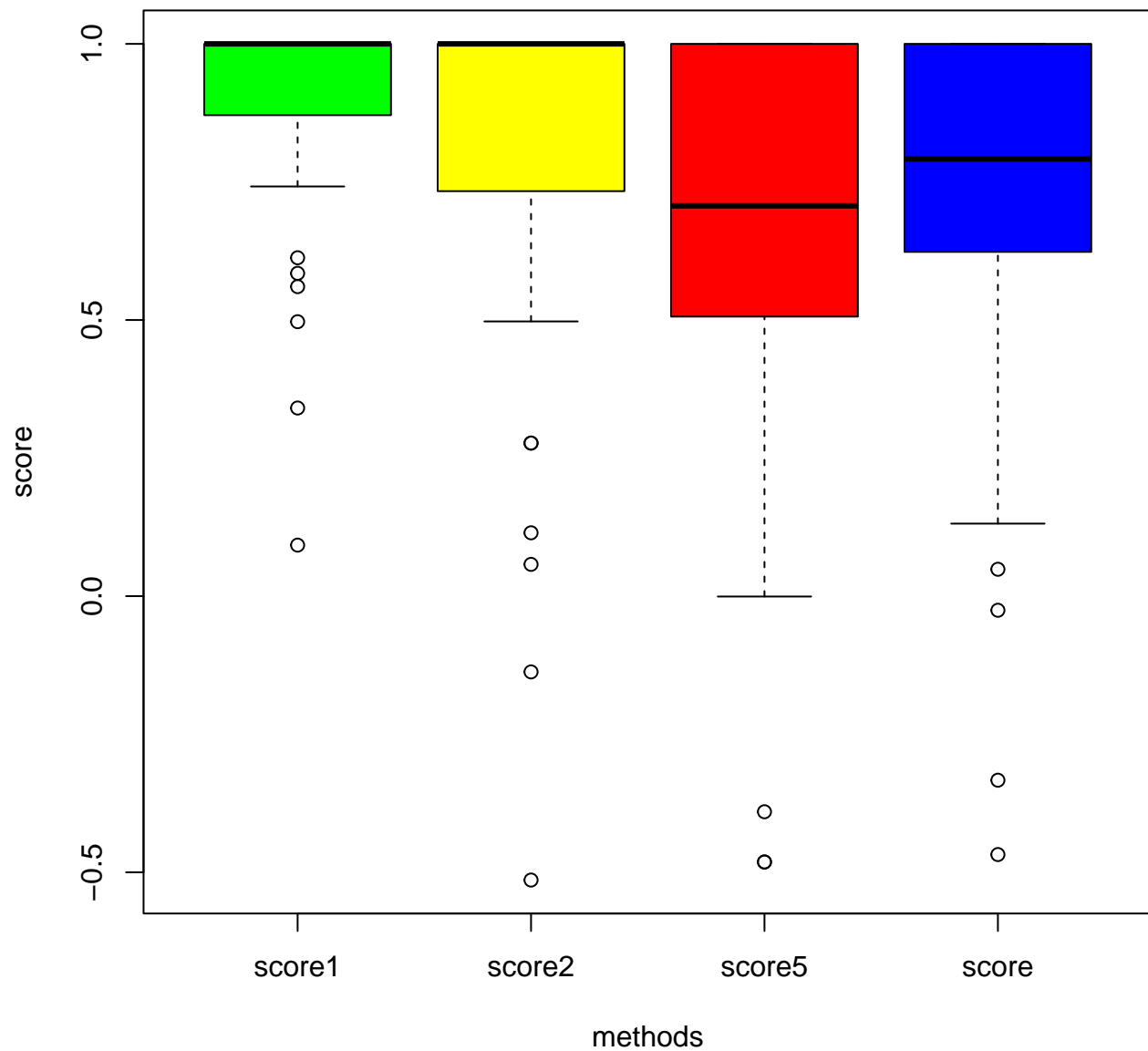

Supplement: Supplementary file 1 [file ijms-25-05267-s001.zip › File S4/2_stability_scores_boxplot/adrenocortical carcinoma_StabBoxplot.pdf]

# bile duct cancer\_stability analysis

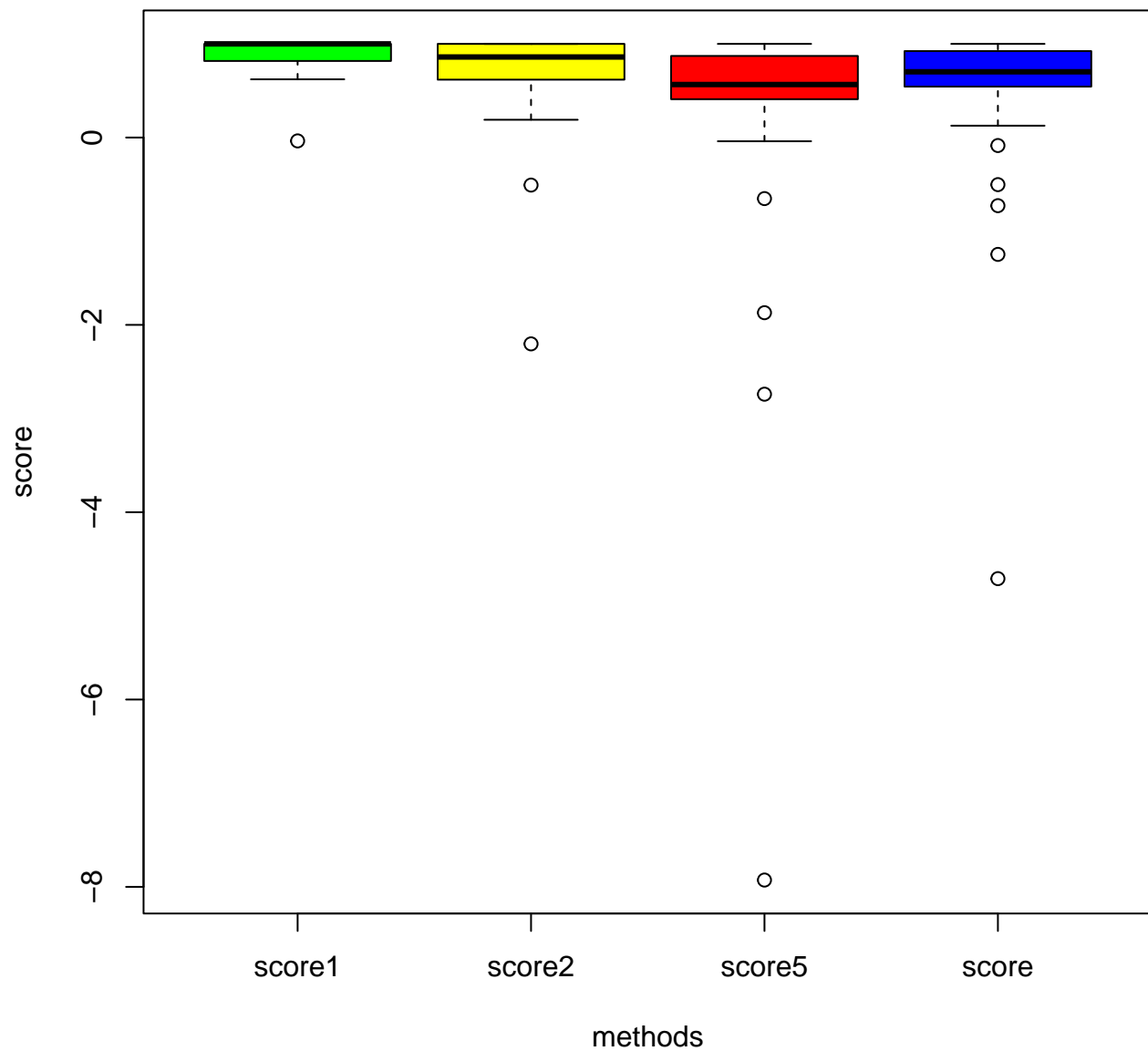

Supplement: Supplementary file 1 [file ijms-25-05267-s001.zip › File S4/2_stability_scores_boxplot/bile duct cancer_StabBoxplot.pdf]

# biliary tract cancer\_stability analysis

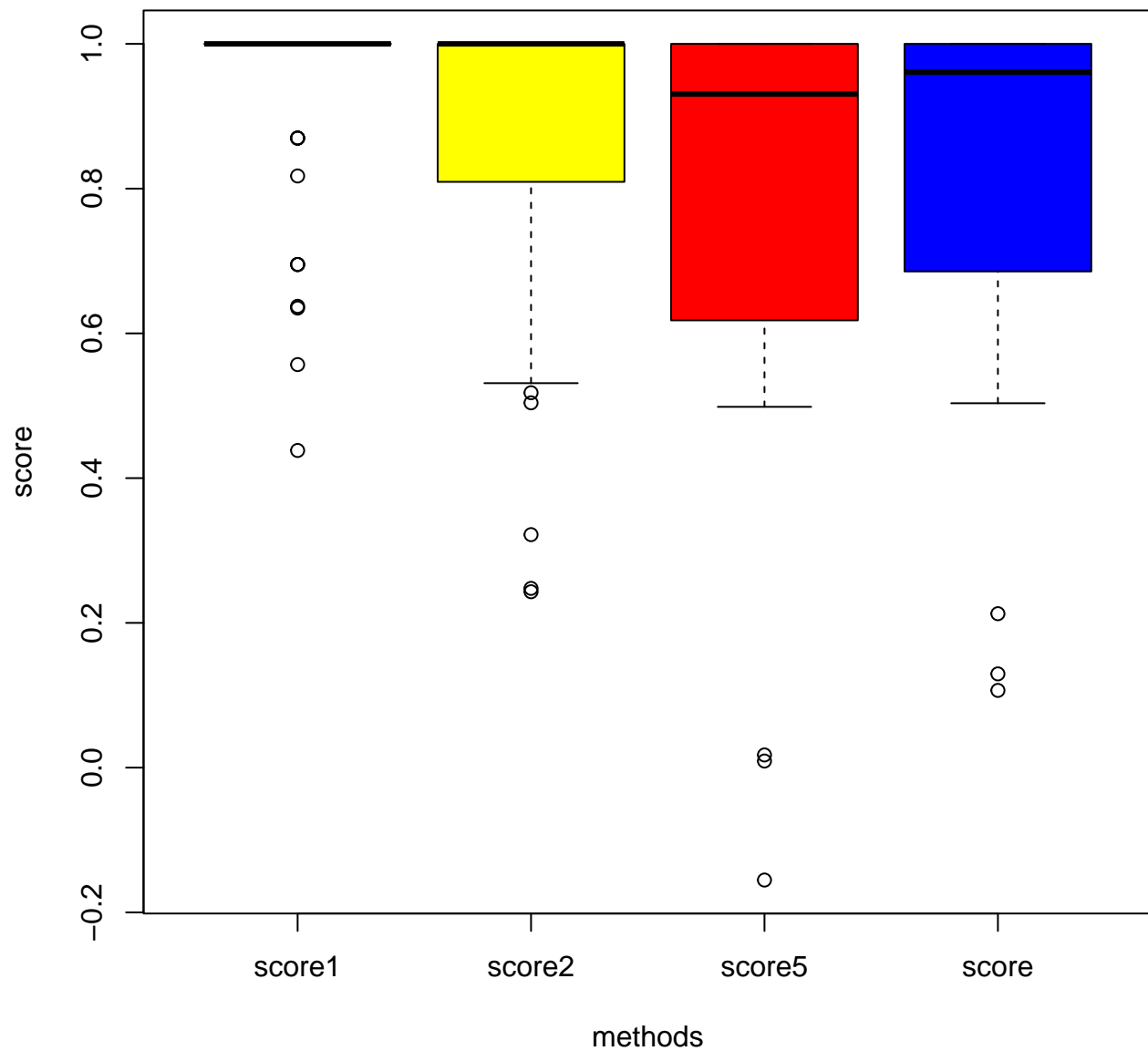

Supplement: Supplementary file 1 [file ijms-25-05267-s001.zip › File S4/2_stability_scores_boxplot/biliary tract cancer_StabBoxplot.pdf]

# bone cancer\_stability analysis

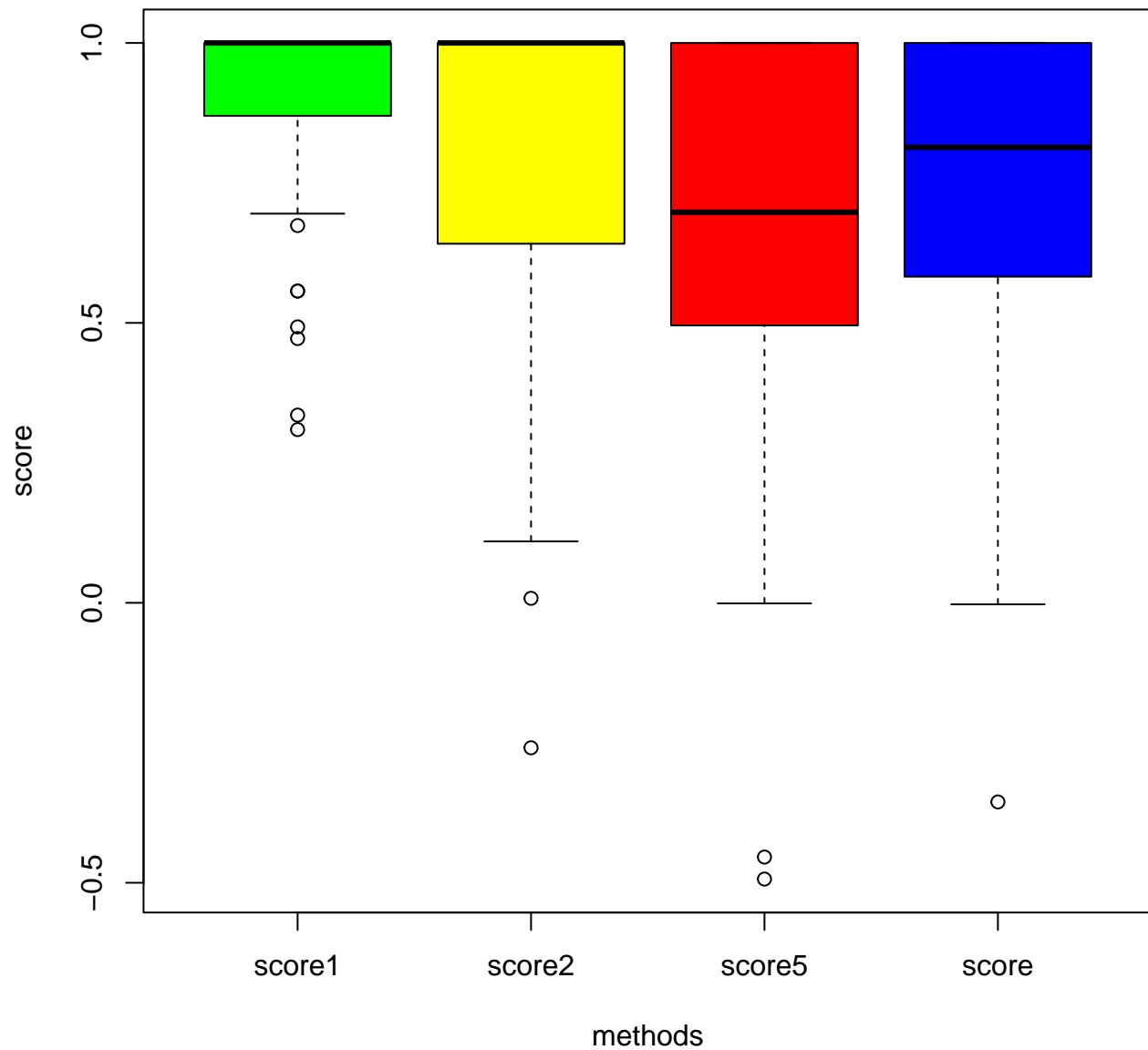

Supplement: Supplementary file 1 [file ijms-25-05267-s001.zip › File S4/2_stability_scores_boxplot/bone cancer_StabBoxplot.pdf]

# brain cancer\_stability analysis

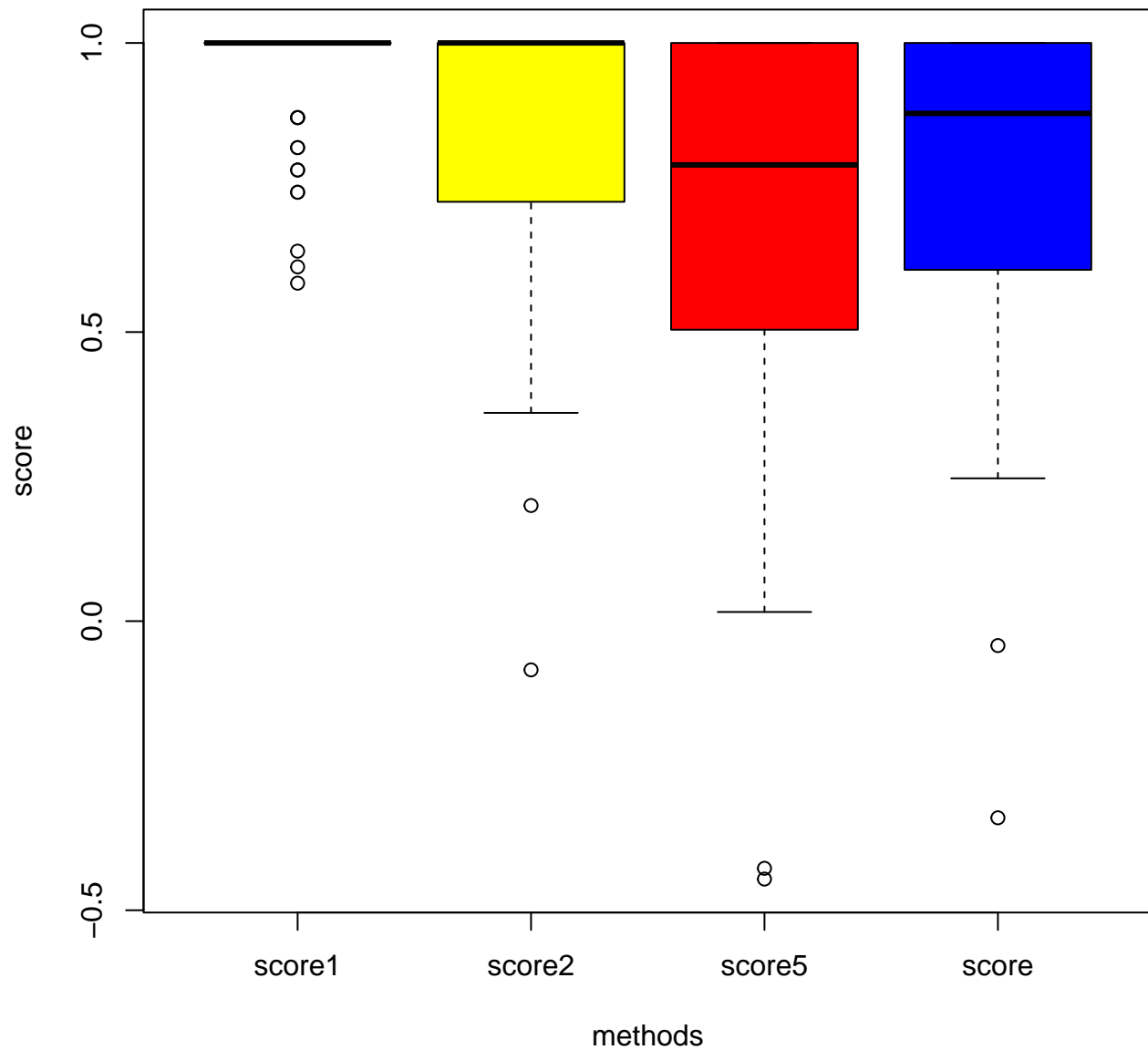

Supplement: Supplementary file 1 [file ijms-25-05267-s001.zip › File S4/2_stability_scores_boxplot/brain cancer_StabBoxplot.pdf]

bronchiolo-alveolar adenocarcinoma\_stability analysis

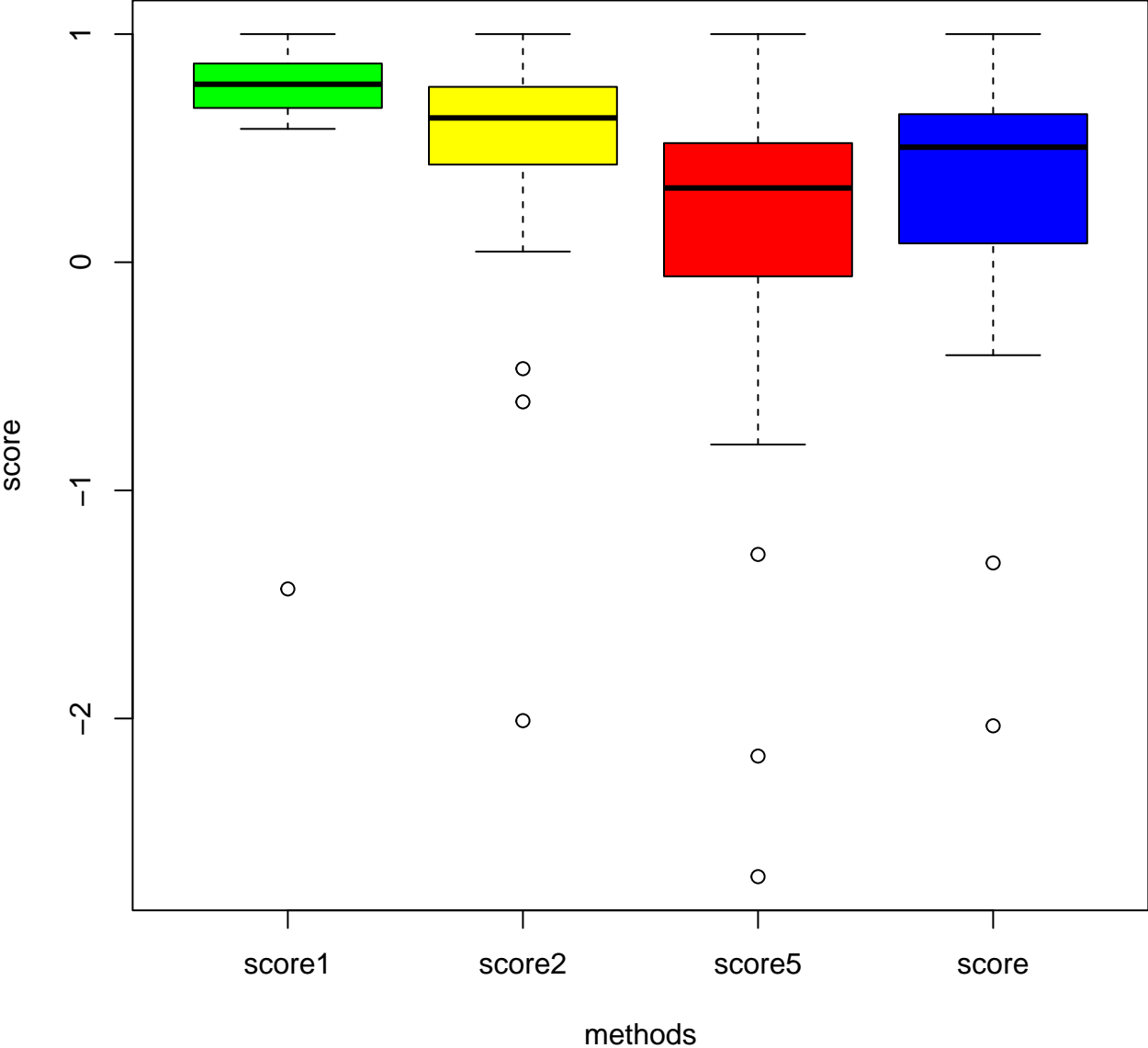

Supplement: Supplementary file 1 [file ijms-25-05267-s001.zip › File S4/2_stability_scores_boxplot/bronchiolo-alveolar adenocarcinoma_StabBoxplot.pdf]

# central nervous system cancer\_stability analysis

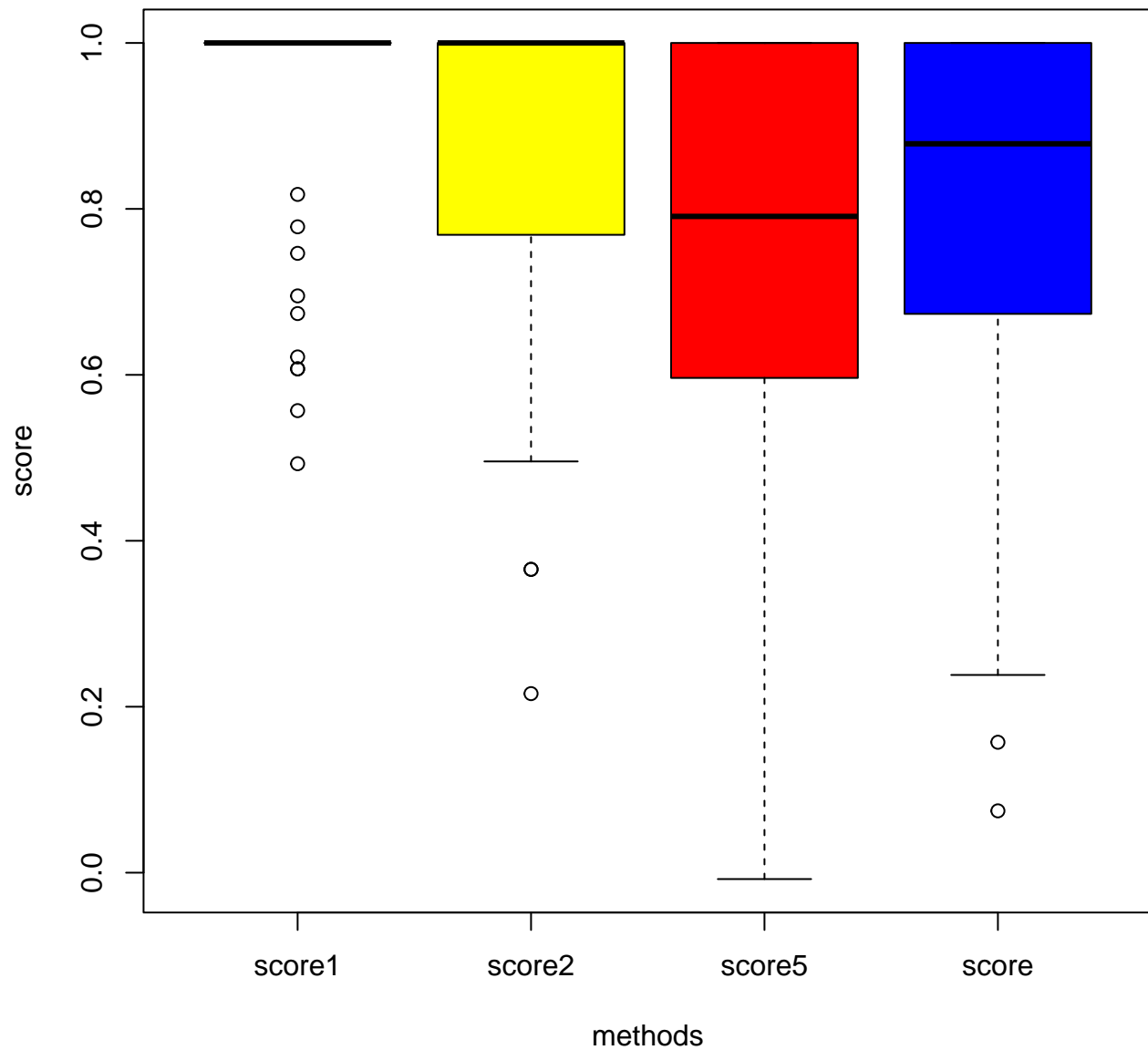

Supplement: Supplementary file 1 [file ijms-25-05267-s001.zip › File S4/2_stability_scores_boxplot/central nervous system cancer_StabBoxplot.pdf]

# cervical cancer\_stability analysis

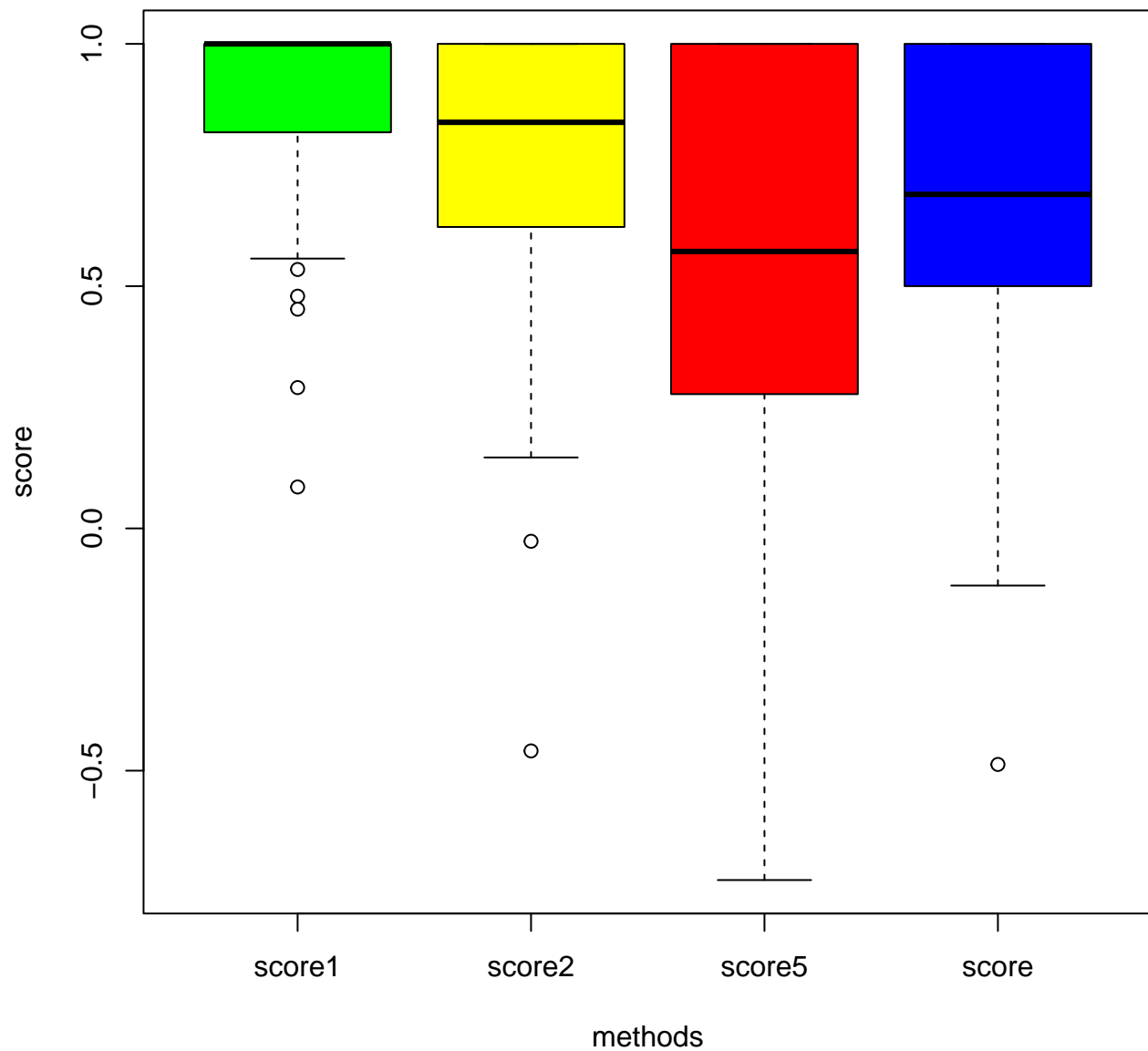

Supplement: Supplementary file 1 [file ijms-25-05267-s001.zip › File S4/2_stability_scores_boxplot/cervical cancer_StabBoxplot.pdf]

# colon cancer\_stability analysis

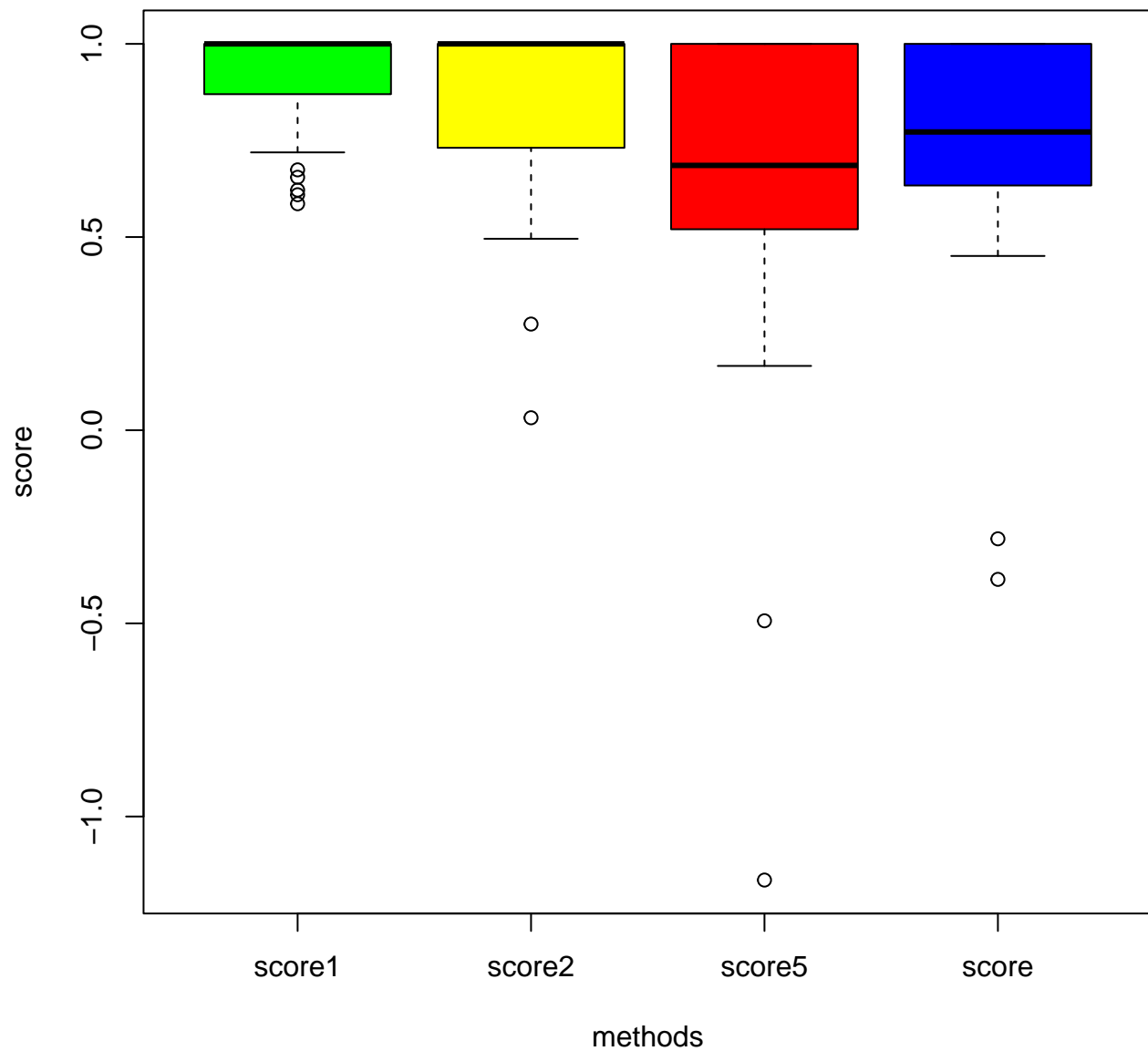

Supplement: Supplementary file 1 [file ijms-25-05267-s001.zip › File S4/2_stability_scores_boxplot/colon cancer_StabBoxplot.pdf]

# connective tissue benign neoplasm\_stability analysis

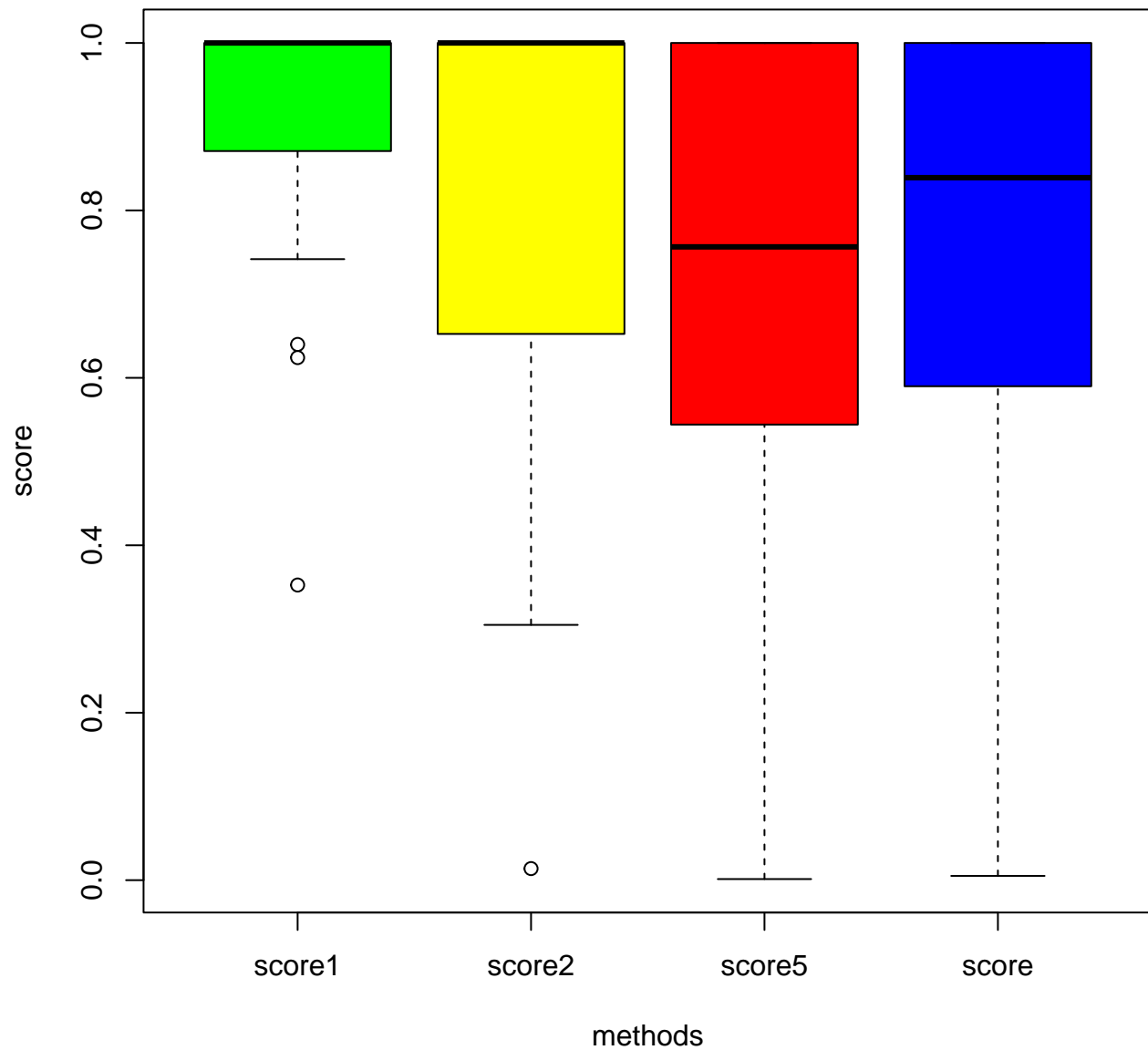

Supplement: Supplementary file 1 [file ijms-25-05267-s001.zip › File S4/2_stability_scores_boxplot/connective tissue benign neoplasm_StabBoxplot.pdf]

# endometrial cancer\_stability analysis

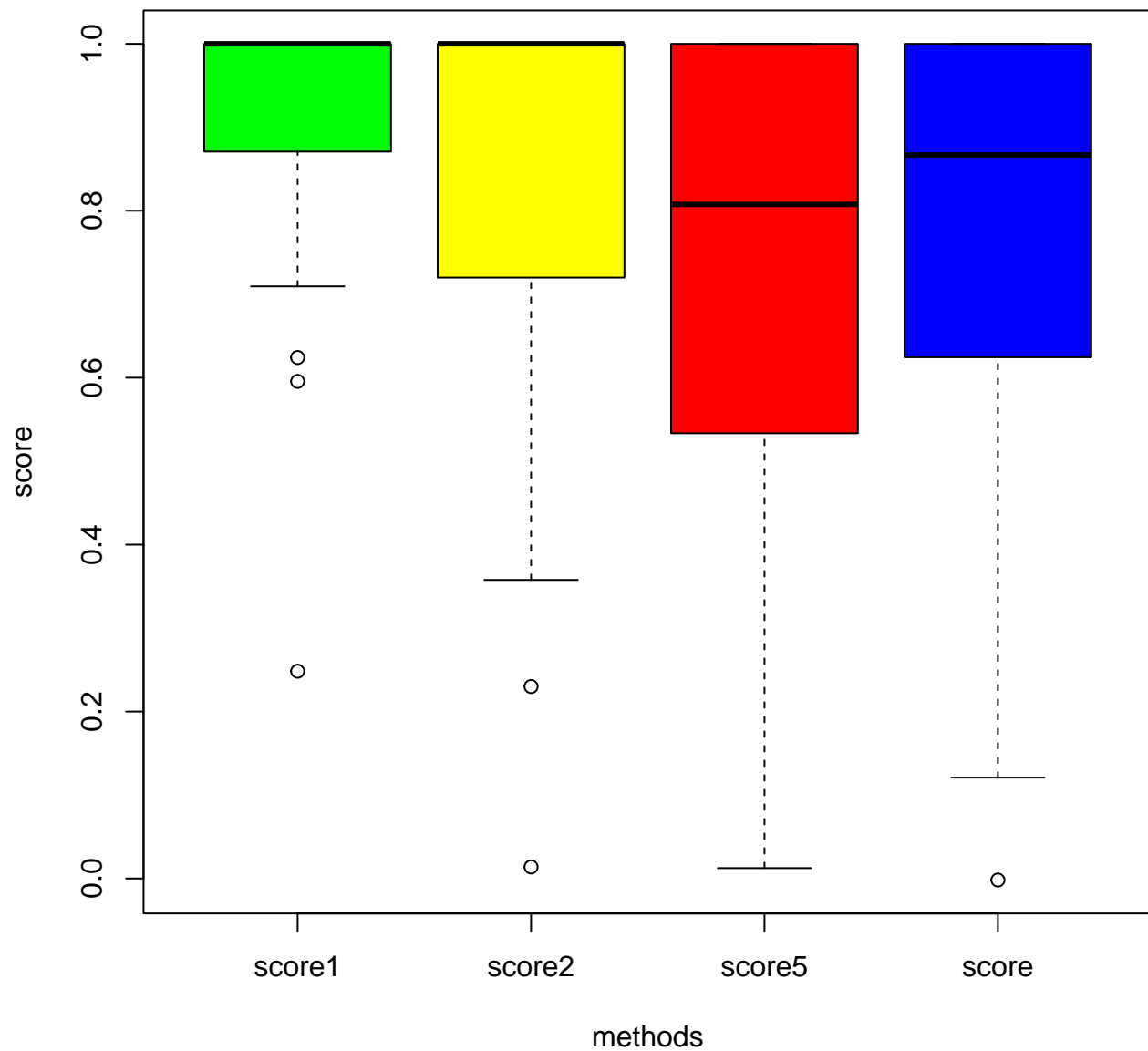

Supplement: Supplementary file 1 [file ijms-25-05267-s001.zip › File S4/2_stability_scores_boxplot/endometrial cancer_StabBoxplot.pdf]

# endometrial carcinoma\_stability analysis

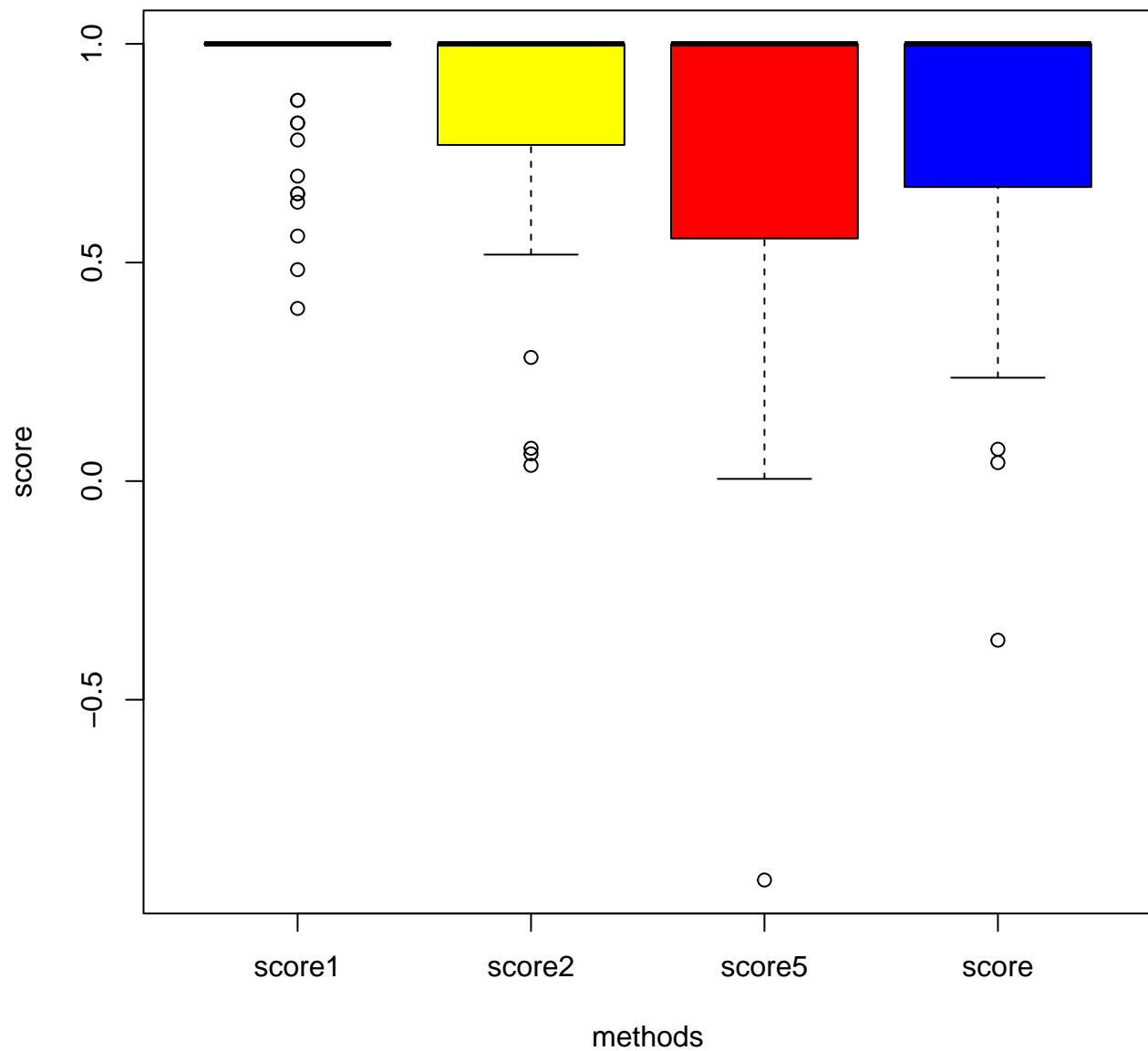

Supplement: Supplementary file 1 [file ijms-25-05267-s001.zip › File S4/2_stability_scores_boxplot/endometrial carcinoma_StabBoxplot.pdf]

# esophageal cancer\_stability analysis

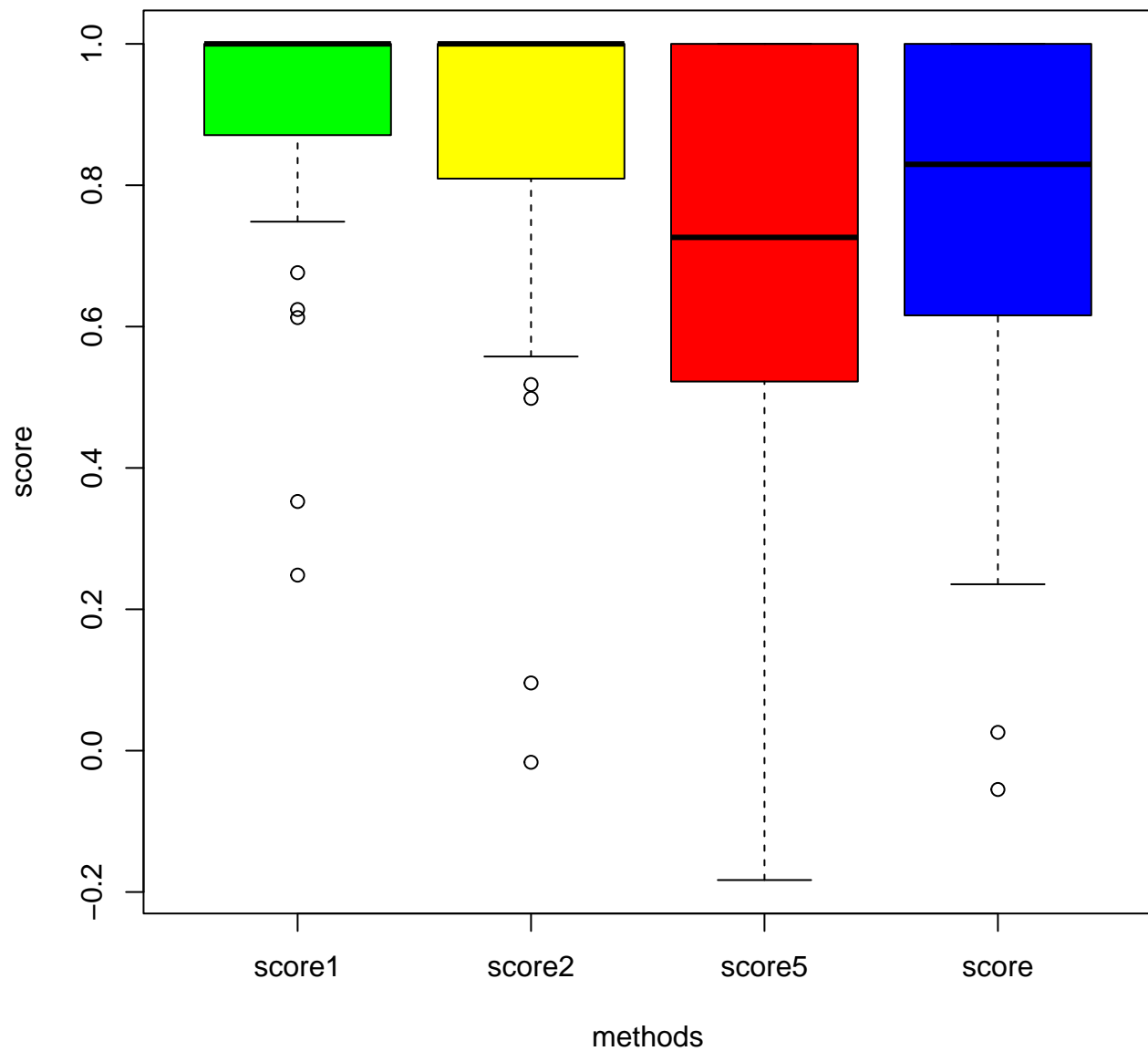

Supplement: Supplementary file 1 [file ijms-25-05267-s001.zip › File S4/2_stability_scores_boxplot/esophageal cancer_StabBoxplot.pdf]

# familial retinoblastoma\_stability analysis

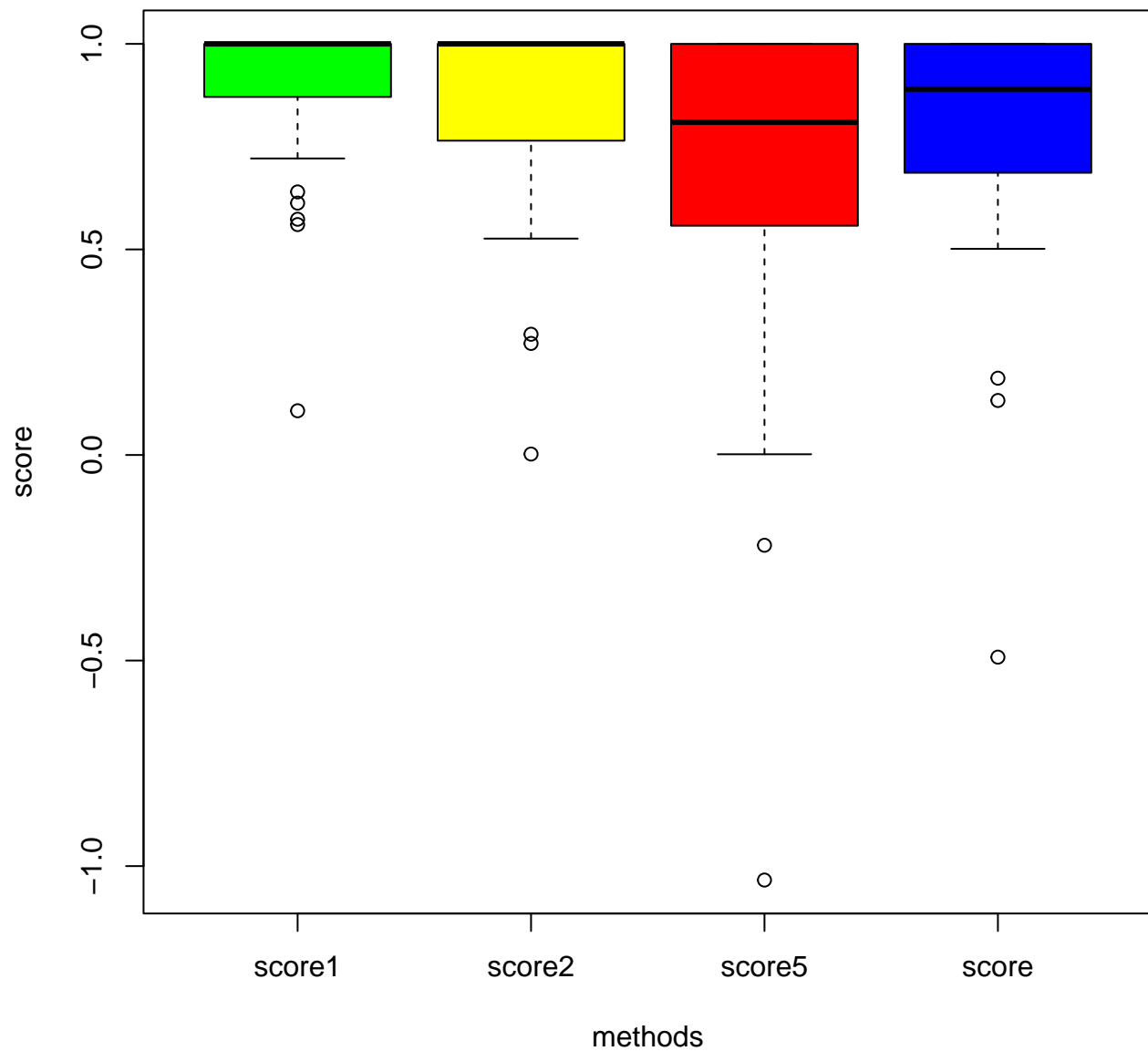

Supplement: Supplementary file 1 [file ijms-25-05267-s001.zip › File S4/2_stability_scores_boxplot/familial retinoblastoma_StabBoxplot.pdf]

# female reproductive organ cancer\_stability analysis

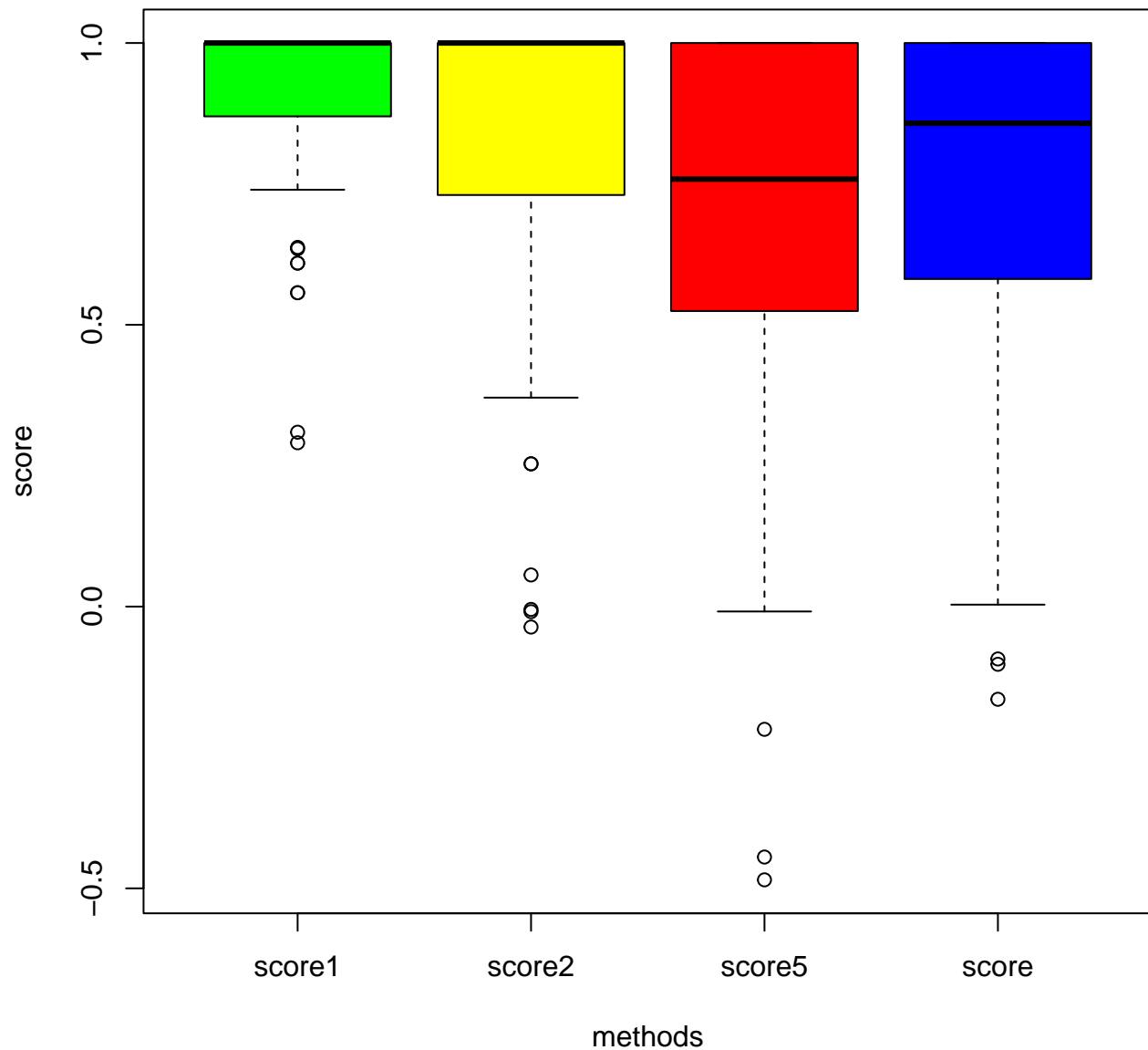

Supplement: Supplementary file 1 [file ijms-25-05267-s001.zip › File S4/2_stability_scores_boxplot/female reproductive organ cancer_StabBoxplot.pdf]

# gallbladder cancer\_stability analysis

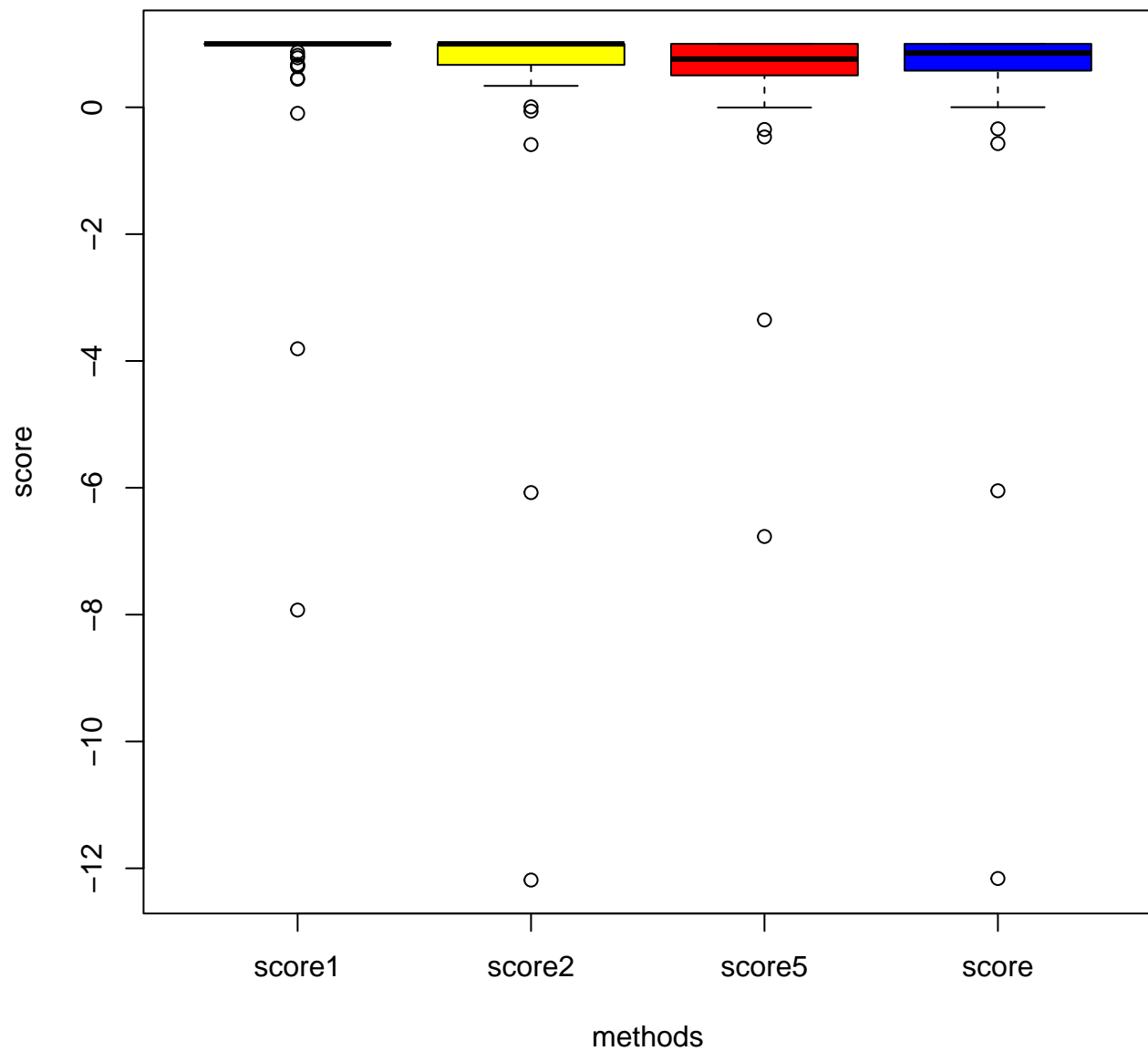

Supplement: Supplementary file 1 [file ijms-25-05267-s001.zip › File S4/2_stability_scores_boxplot/gallbladder cancer_StabBoxplot.pdf]

# growth hormone secreting pituitary adenoma\_stability analysis

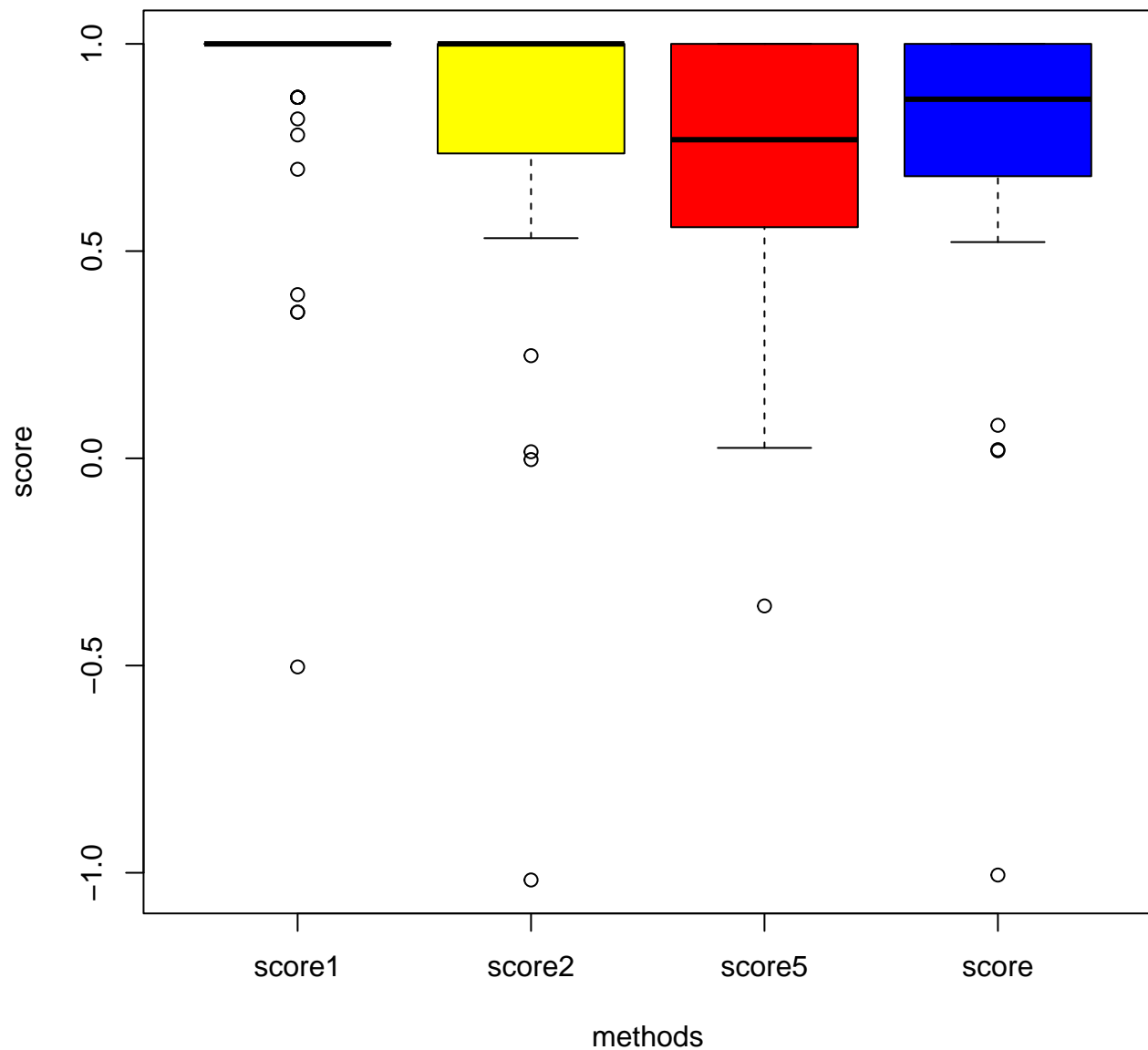

Supplement: Supplementary file 1 [file ijms-25-05267-s001.zip › File S4/2_stability_scores_boxplot/growth hormone secreting pituitary adenoma_StabBoxplot.pdf]

# head and neck cancer\_stability analysis

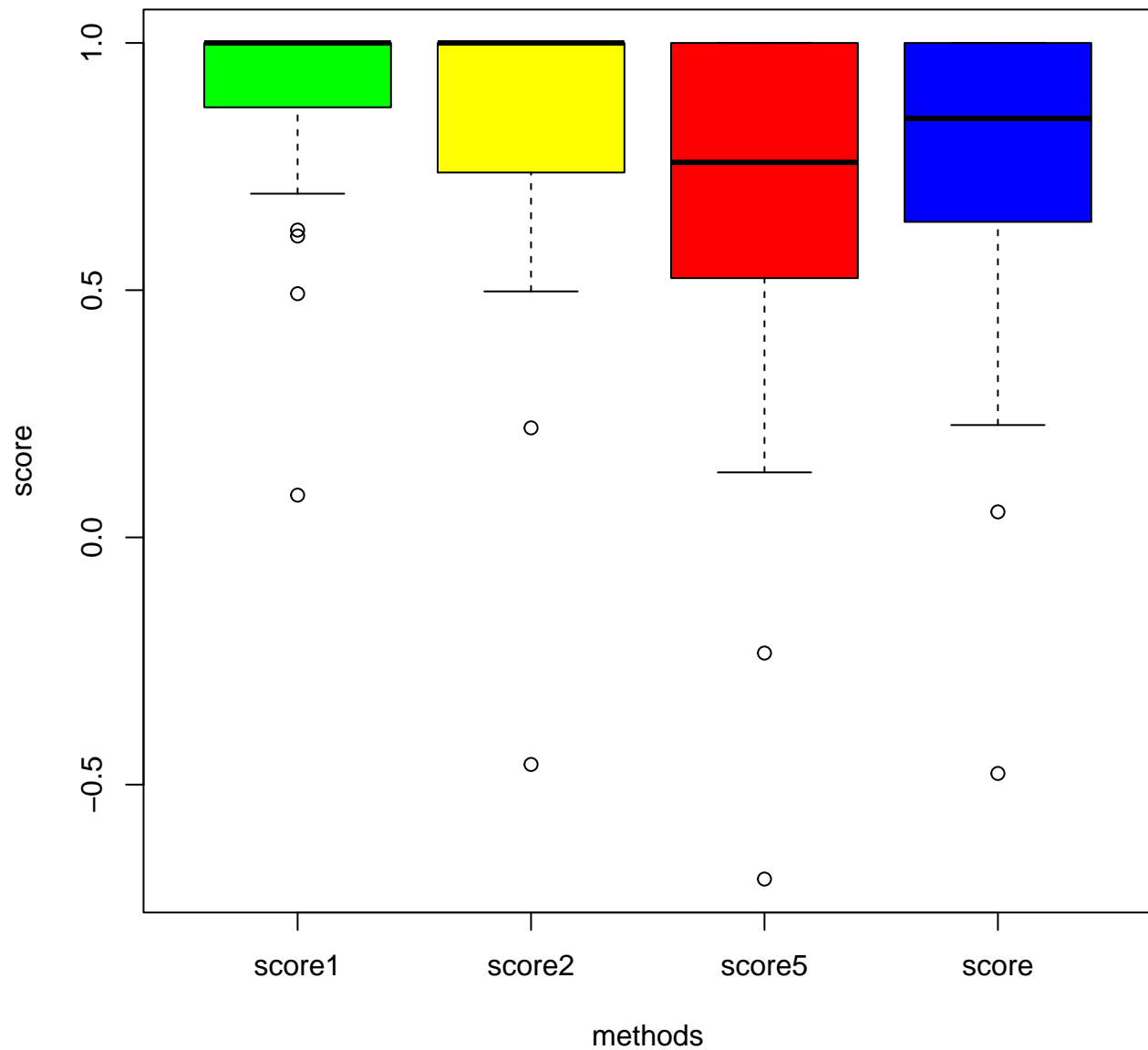

Supplement: Supplementary file 1 [file ijms-25-05267-s001.zip › File S4/2_stability_scores_boxplot/head and neck cancer_StabBoxplot.pdf]

# inflammatory breast carcinoma\_stability analysis

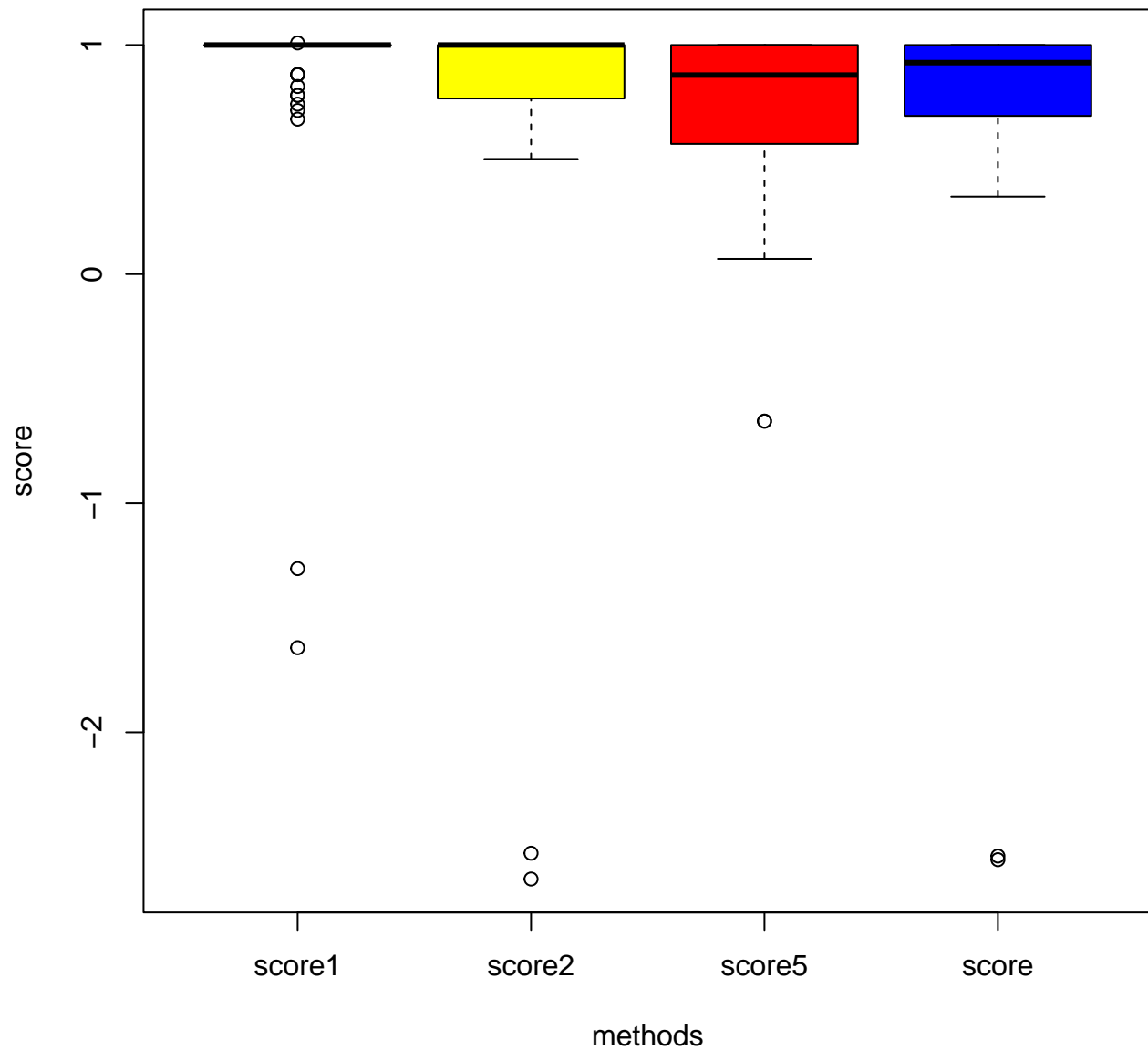

Supplement: Supplementary file 1 [file ijms-25-05267-s001.zip › File S4/2_stability_scores_boxplot/inflammatory breast carcinoma_StabBoxplot.pdf]

# intestinal cancer\_stability analysis

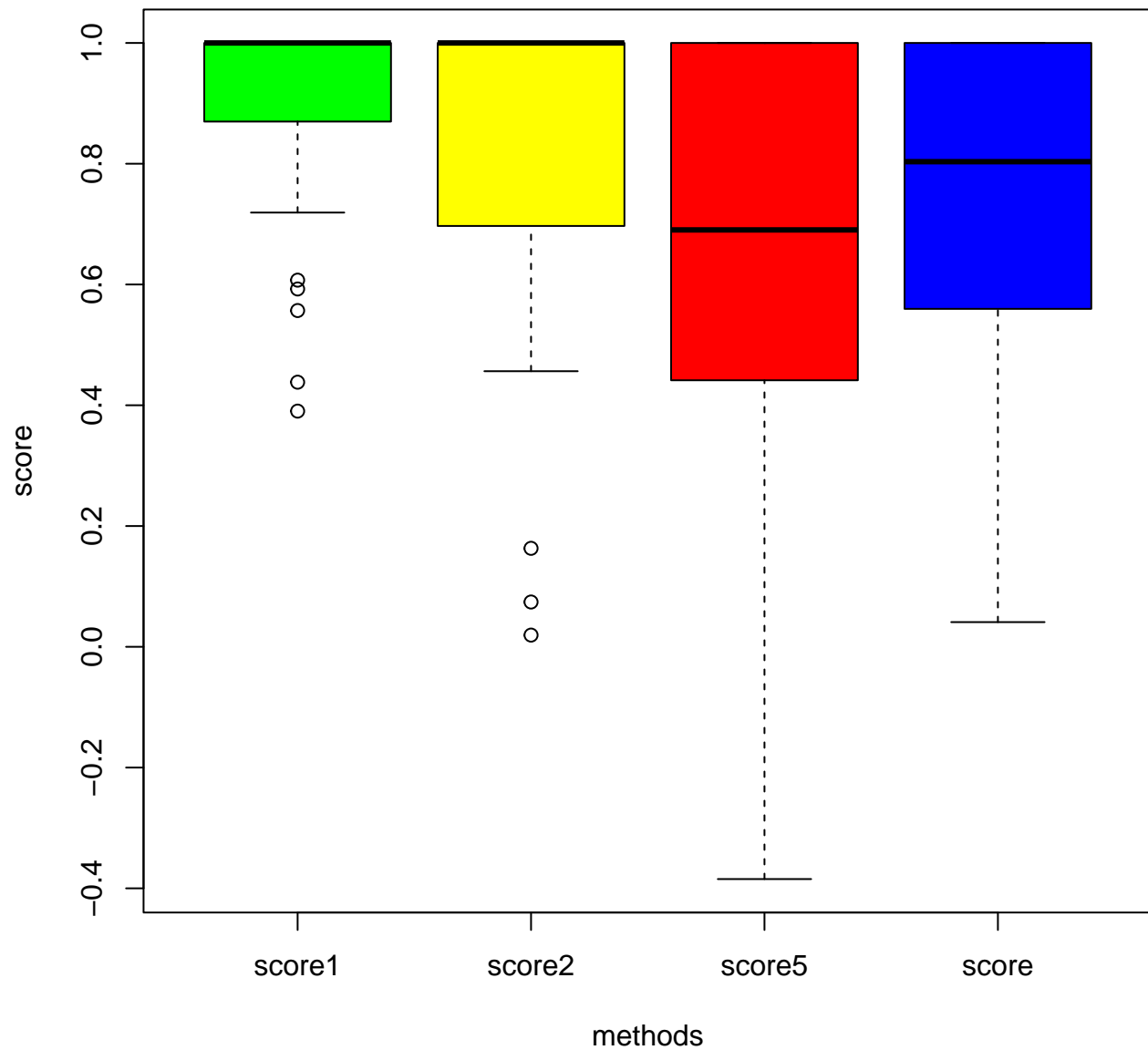

Supplement: Supplementary file 1 [file ijms-25-05267-s001.zip › File S4/2_stability_scores_boxplot/intestinal cancer_StabBoxplot.pdf]

# invasive lobular carcinoma\_stability analysis

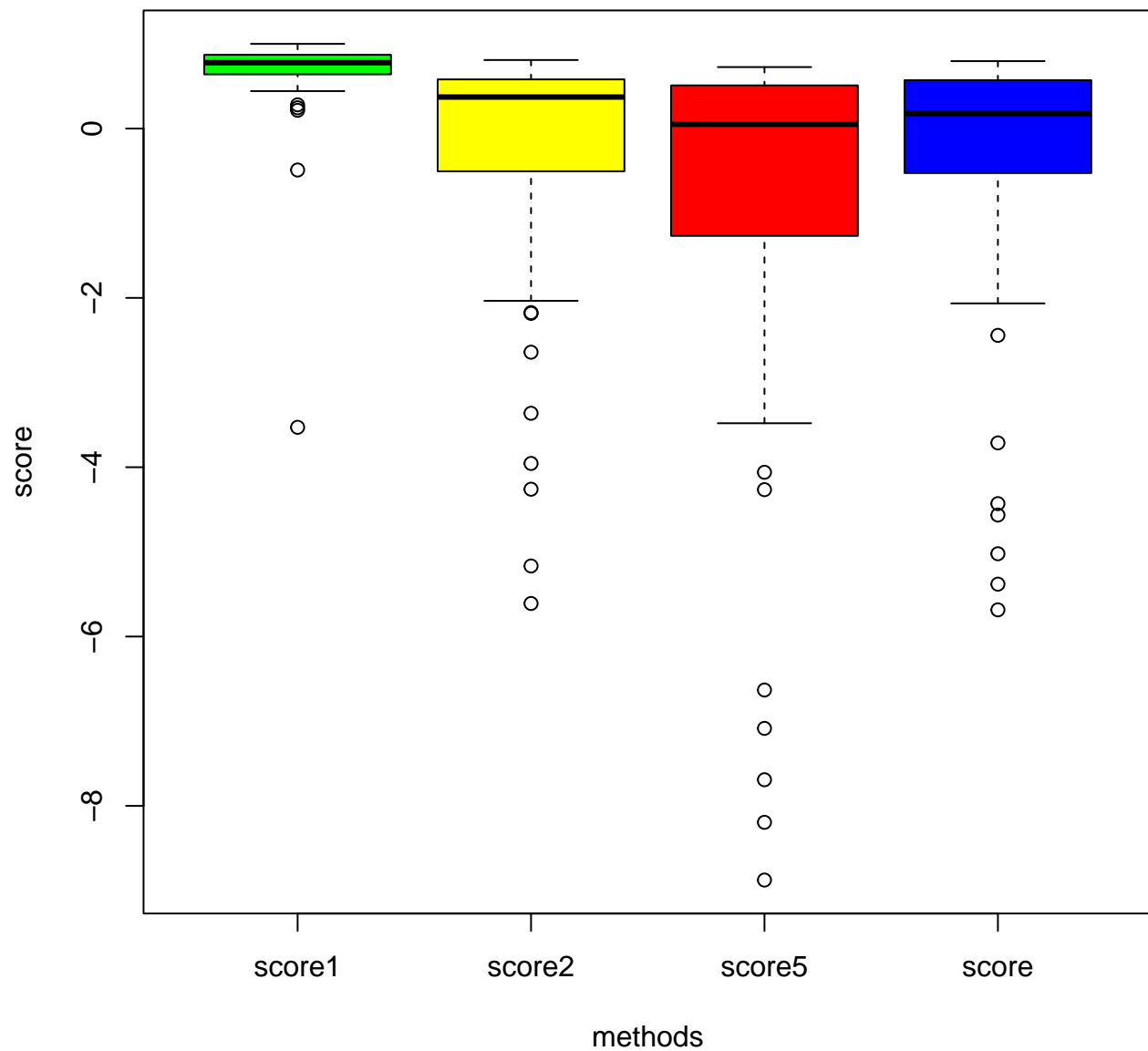

Supplement: Supplementary file 1 [file ijms-25-05267-s001.zip › File S4/2_stability_scores_boxplot/invasive lobular carcinoma_StabBoxplot.pdf]

# kidney cancer\_stability analysis

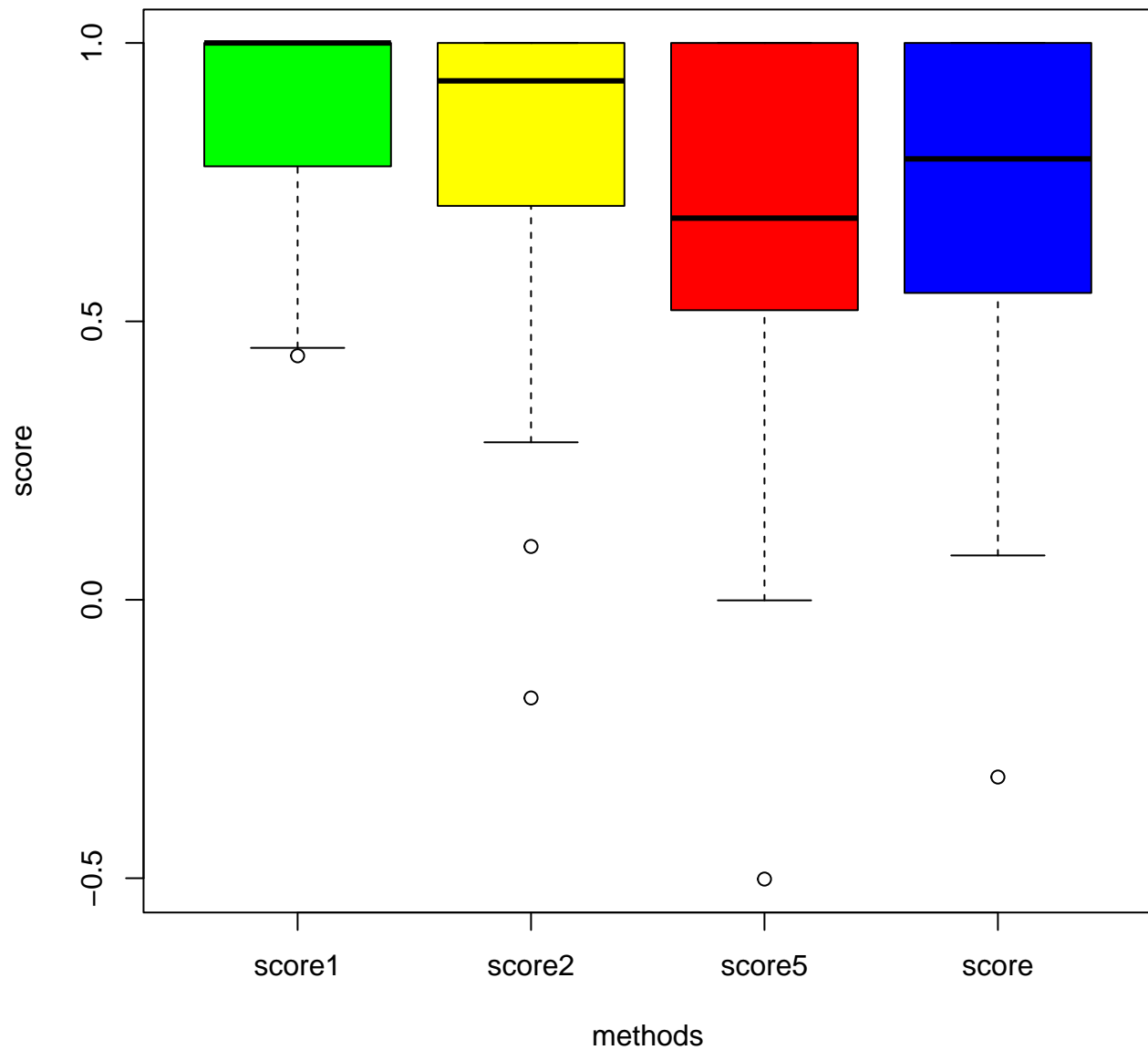

Supplement: Supplementary file 1 [file ijms-25-05267-s001.zip › File S4/2_stability_scores_boxplot/kidney cancer_StabBoxplot.pdf]

# large intestine cancer\_stability analysis

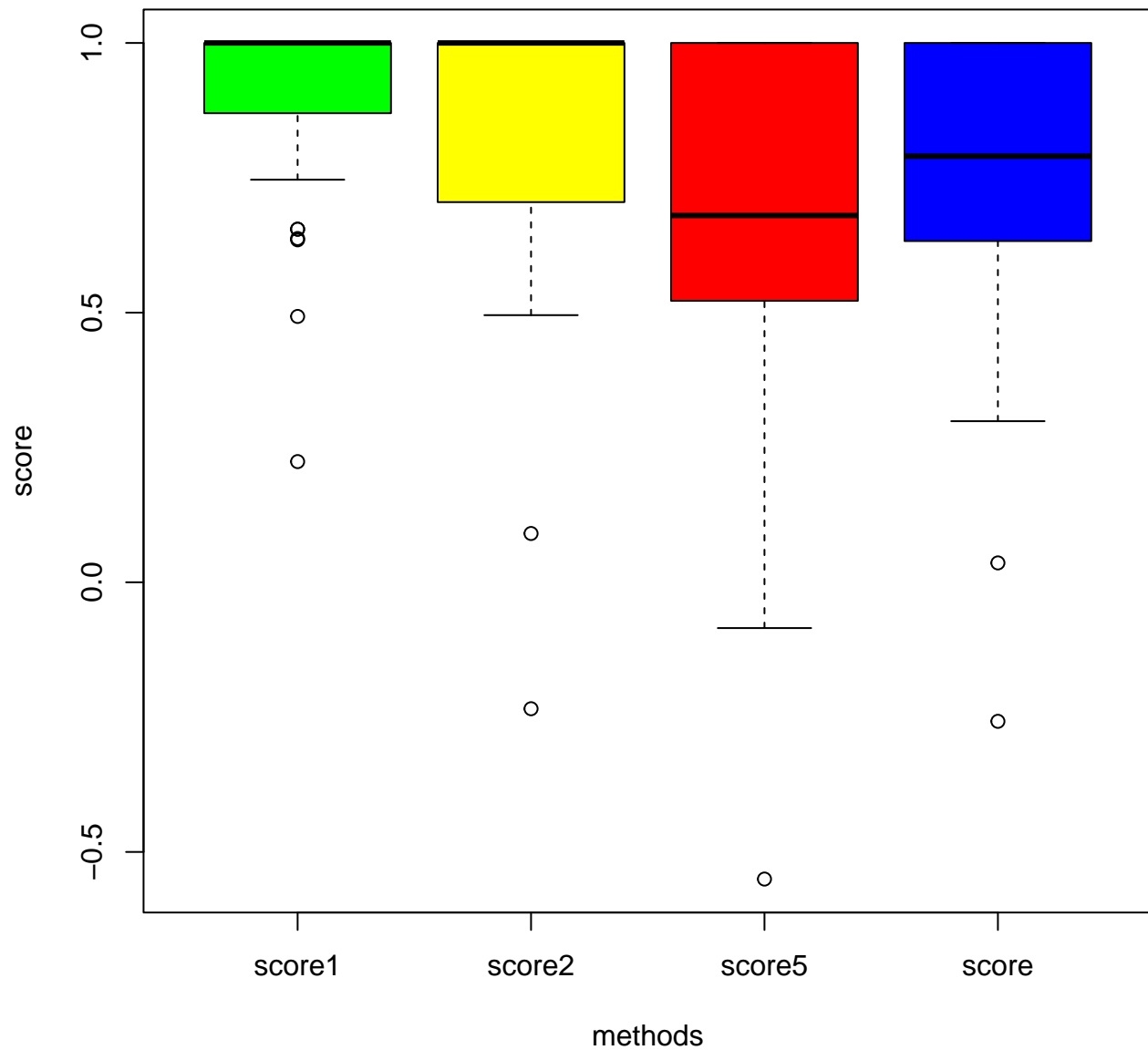

Supplement: Supplementary file 1 [file ijms-25-05267-s001.zip › File S4/2_stability_scores_boxplot/large intestine cancer_StabBoxplot.pdf]

# larynx cancer\_stability analysis

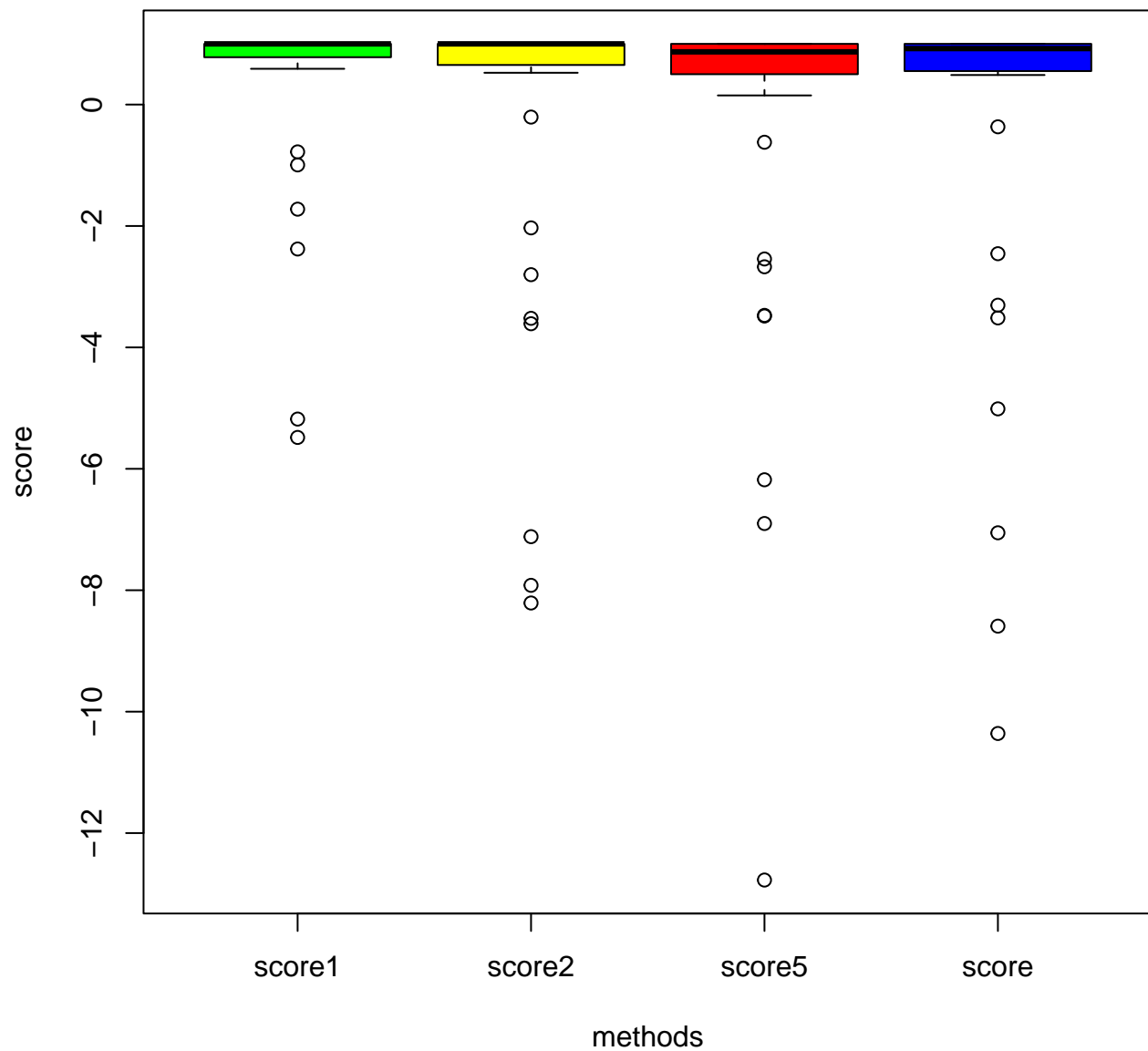

Supplement: Supplementary file 1 [file ijms-25-05267-s001.zip › File S4/2_stability_scores_boxplot/larynx cancer_StabBoxplot.pdf]

## lung benign neoplasm\_stability analysis

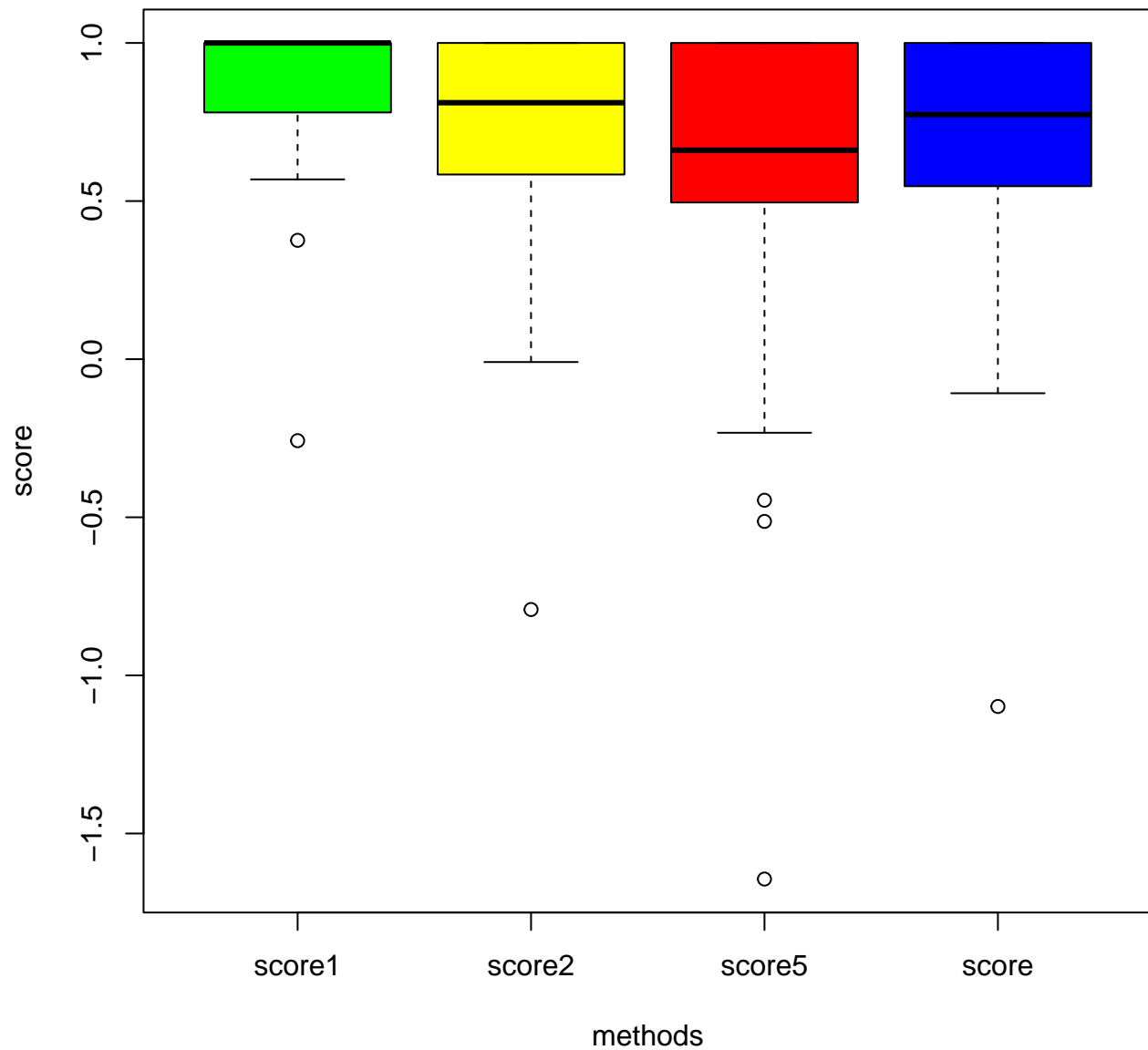

Supplement: Supplementary file 1 [file ijms-25-05267-s001.zip › File S4/2_stability_scores_boxplot/lung benign neoplasm_StabBoxplot.pdf]

# lung small cell carcinoma\_stability analysis

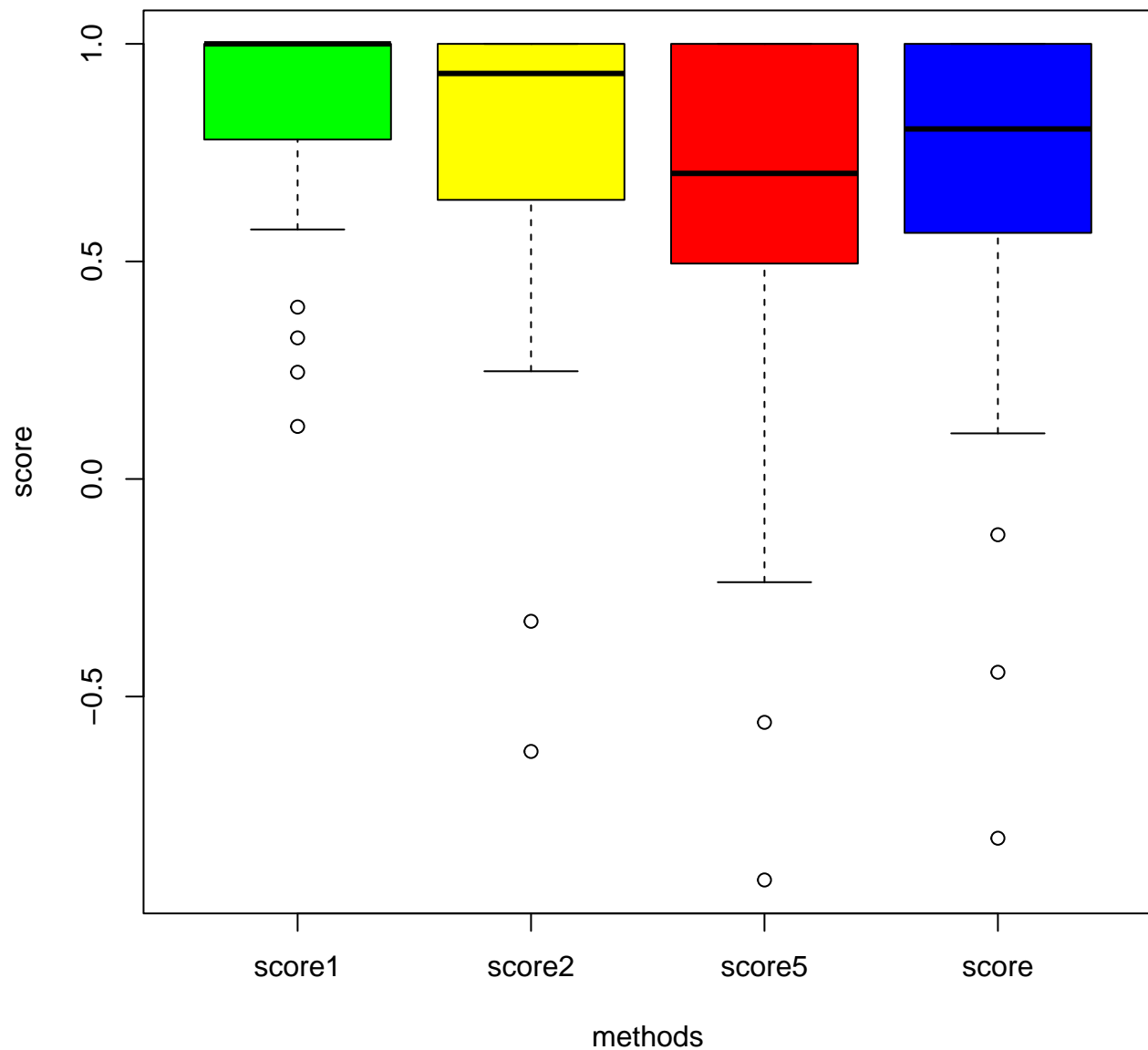

Supplement: Supplementary file 1 [file ijms-25-05267-s001.zip › File S4/2_stability_scores_boxplot/lung small cell carcinoma_StabBoxplot.pdf]

# male breast cancer\_stability analysis

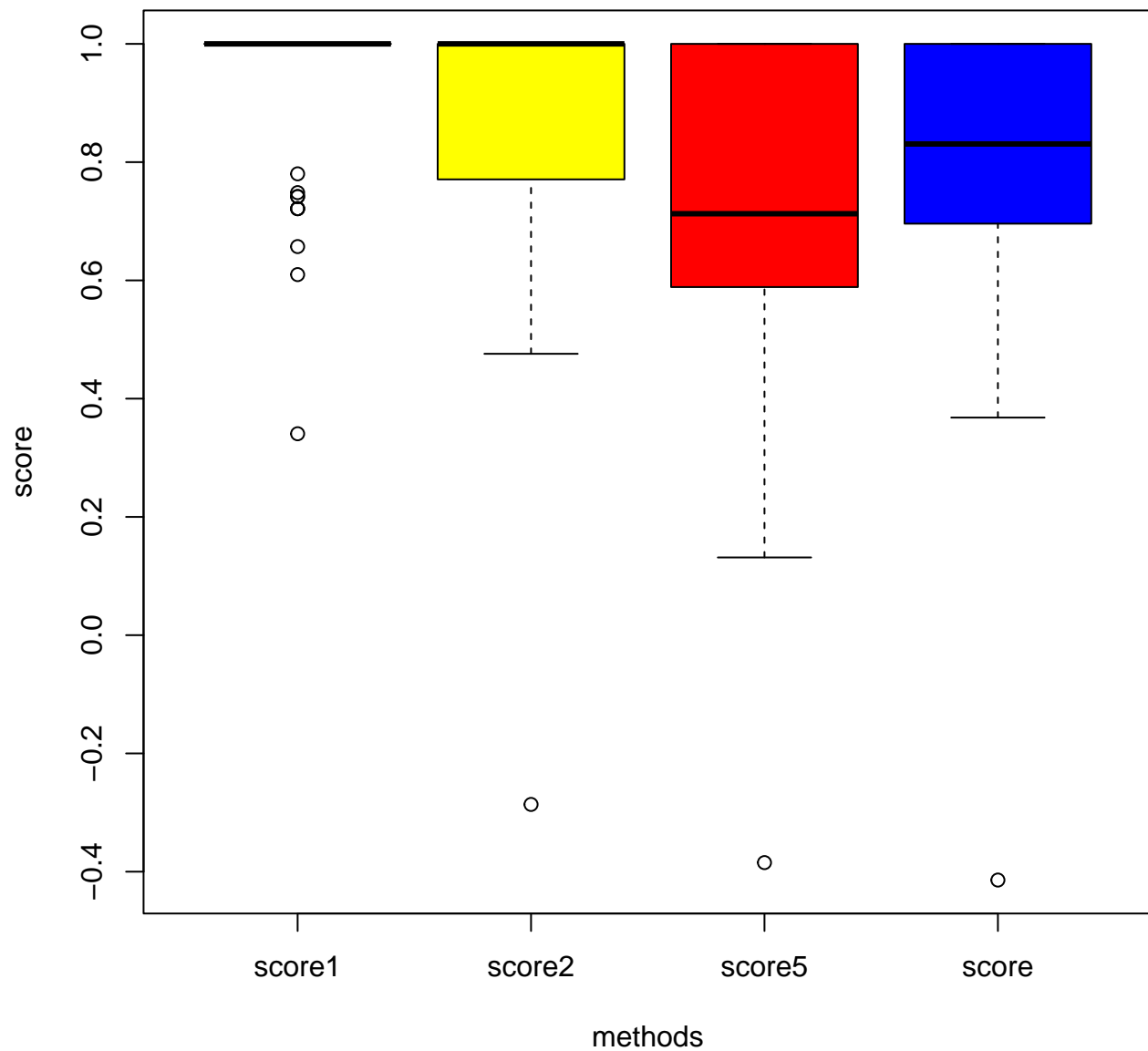

Supplement: Supplementary file 1 [file ijms-25-05267-s001.zip › File S4/2_stability_scores_boxplot/male breast cancer_StabBoxplot.pdf]

# malignant mesothelioma\_stability analysis

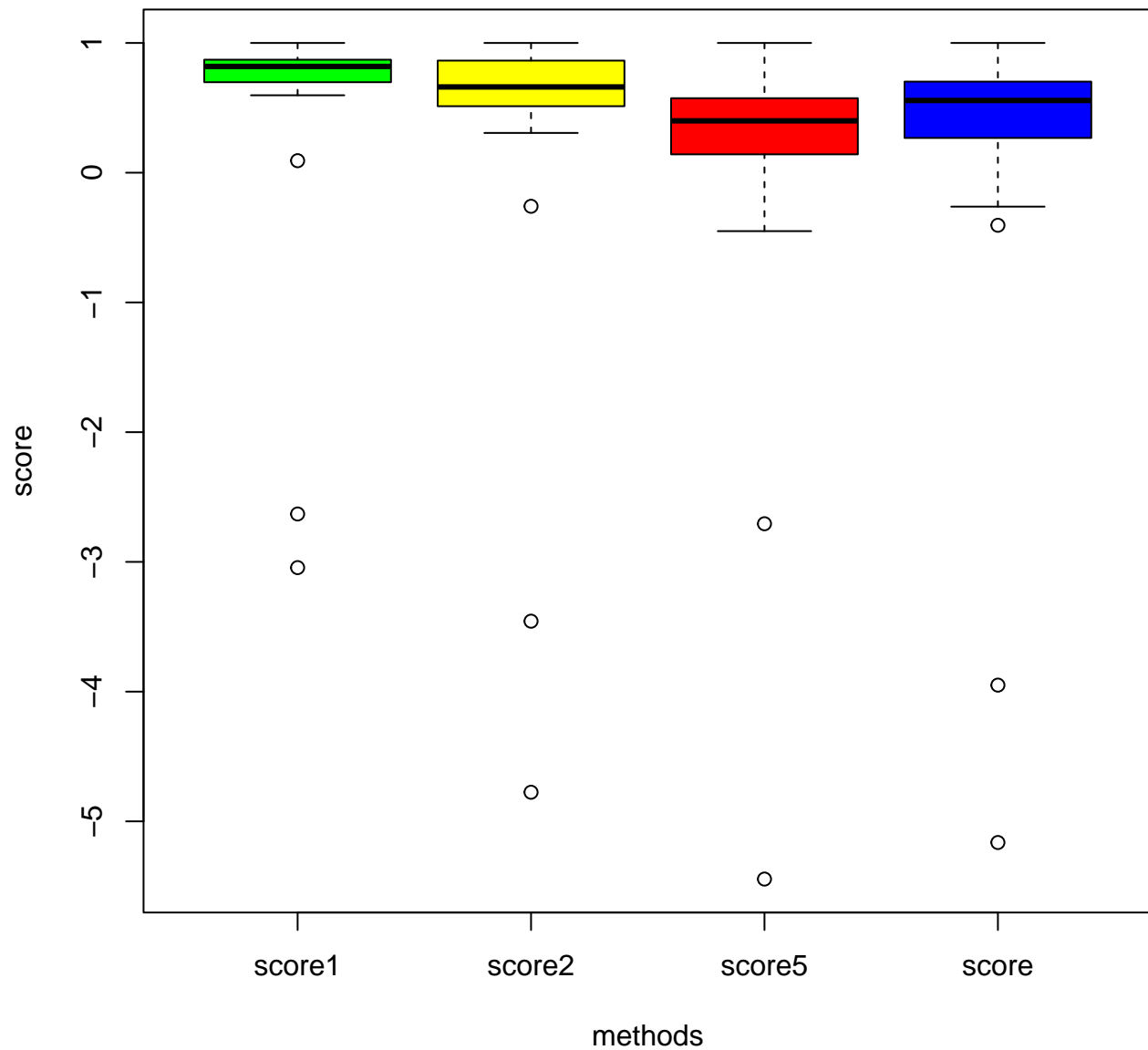

Supplement: Supplementary file 1 [file ijms-25-05267-s001.zip › File S4/2_stability_scores_boxplot/malignant mesothelioma_StabBoxplot.pdf]

# myasthenia gravis\_stability analysis

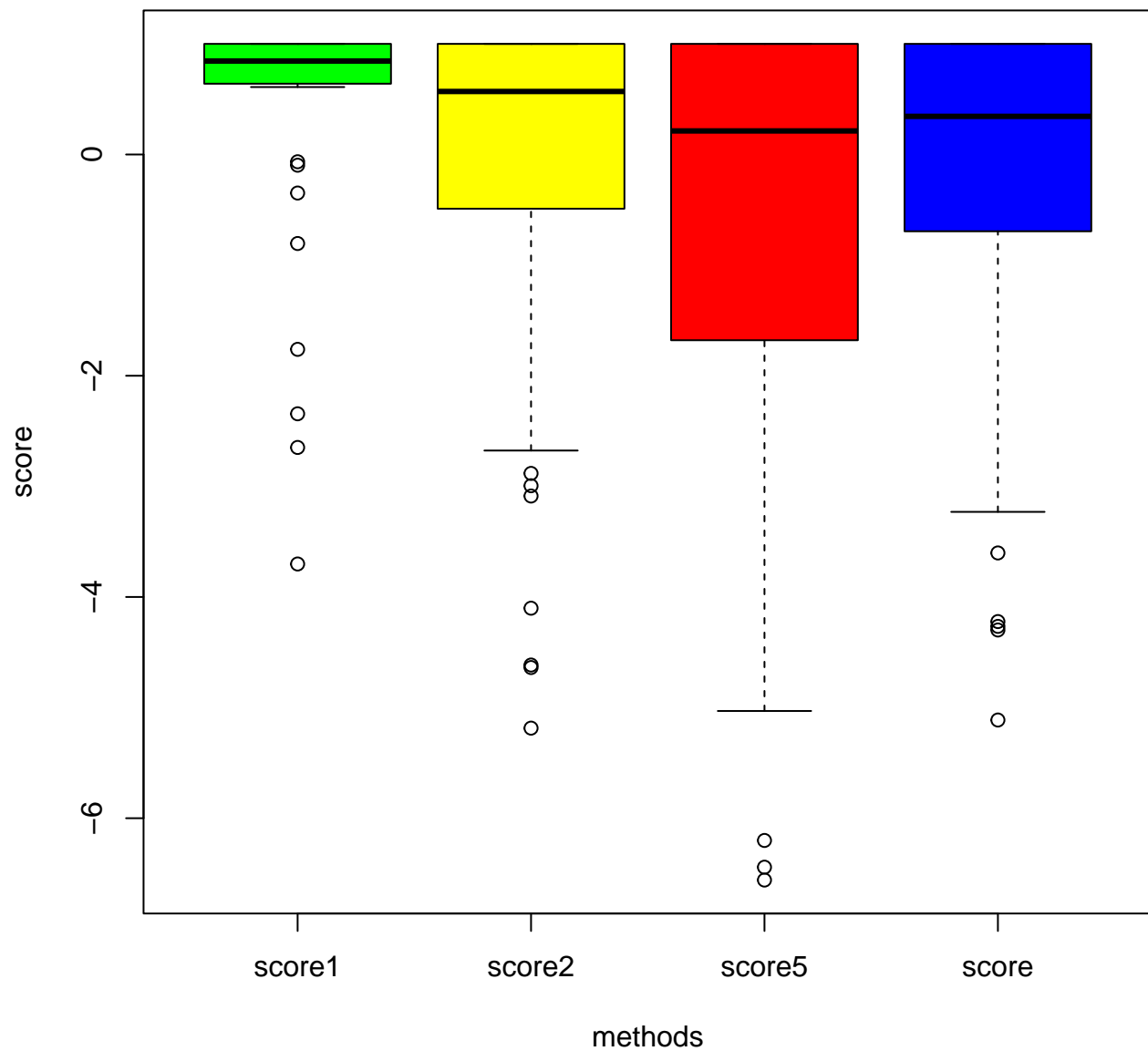

Supplement: Supplementary file 1 [file ijms-25-05267-s001.zip › File S4/2_stability_scores_boxplot/myasthenia gravis_StabBoxplot.pdf]

# nasopharynx carcinoma\_stability analysis

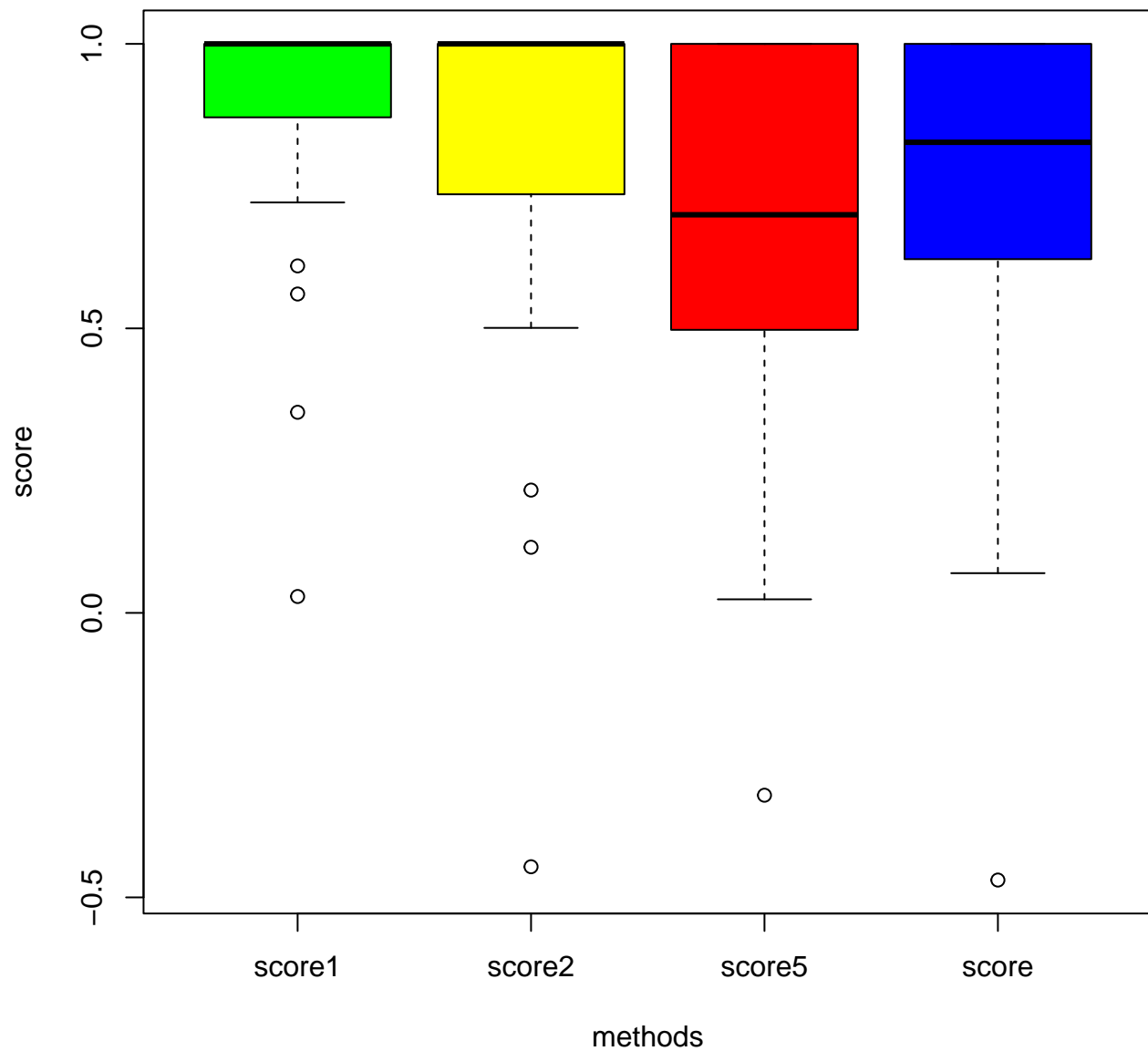

Supplement: Supplementary file 1 [file ijms-25-05267-s001.zip › File S4/2_stability_scores_boxplot/nasopharynx carcinoma_StabBoxplot.pdf]

# nephroblastoma\_stability analysis

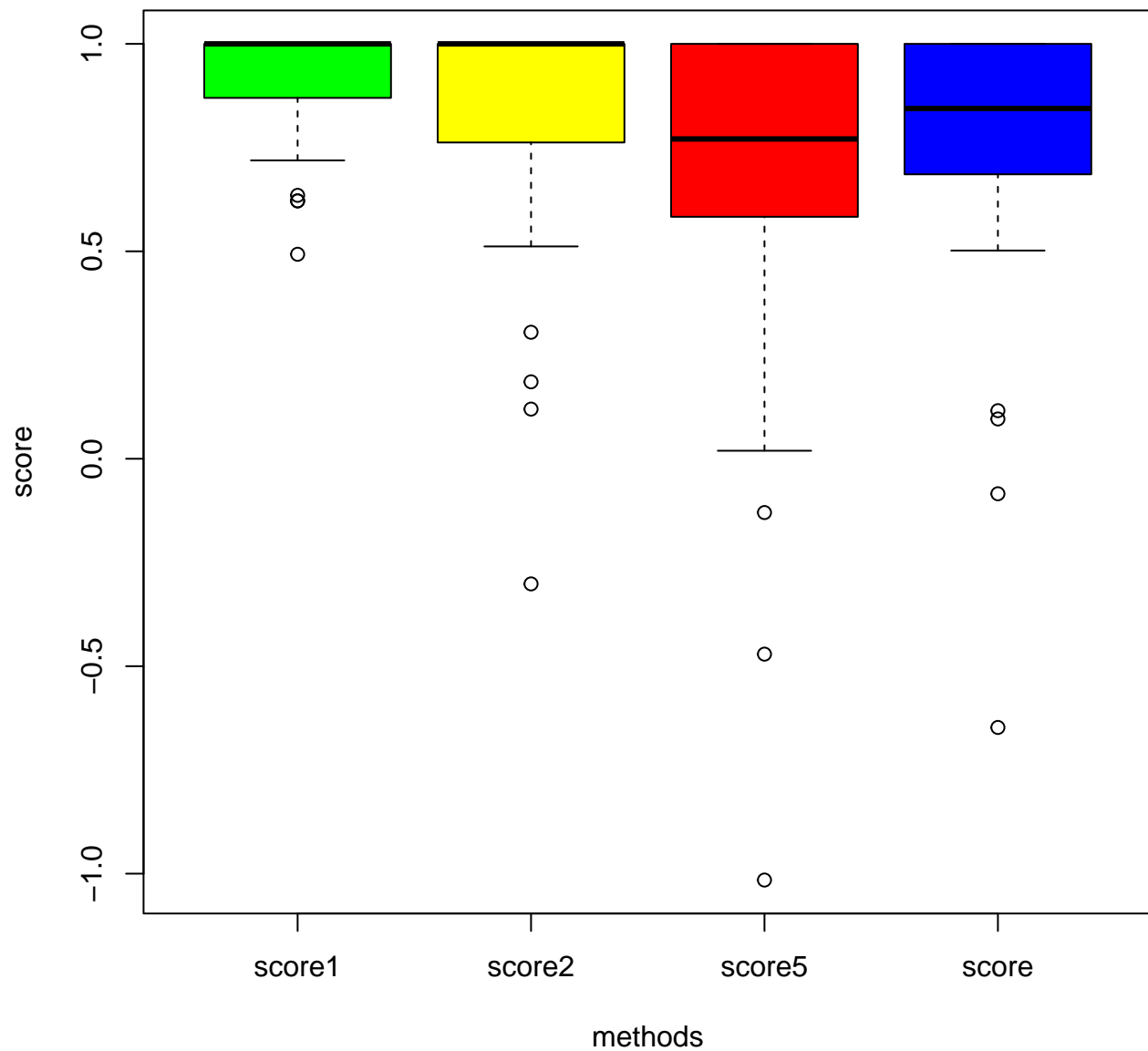

Supplement: Supplementary file 1 [file ijms-25-05267-s001.zip › File S4/2_stability_scores_boxplot/nephroblastoma_StabBoxplot.pdf]

# non-small cell lung carcinoma\_stability analysis

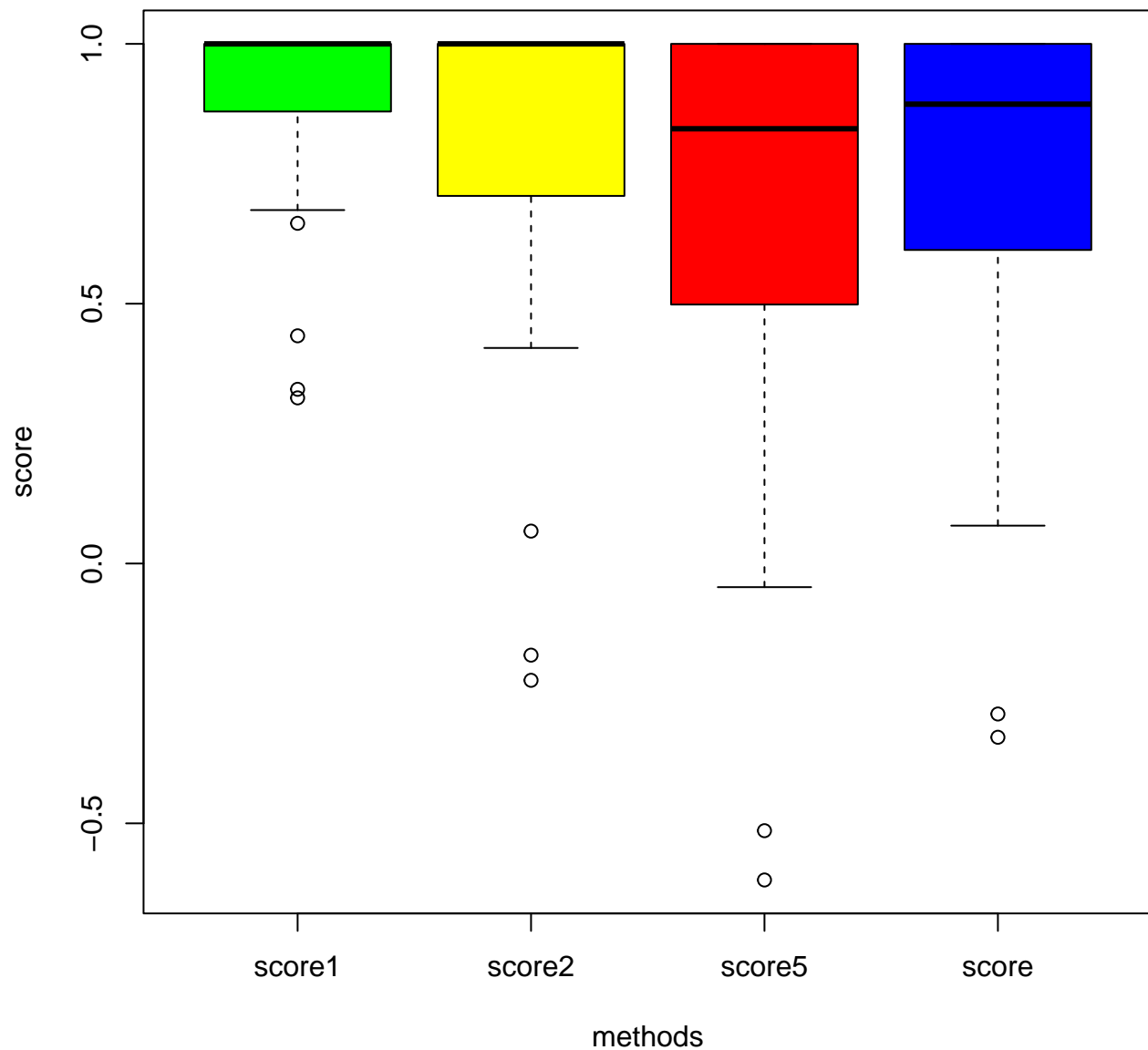

Supplement: Supplementary file 1 [file ijms-25-05267-s001.zip › File S4/2_stability_scores_boxplot/non-small cell lung carcinoma_StabBoxplot.pdf]

# ovarian cancer\_stability analysis

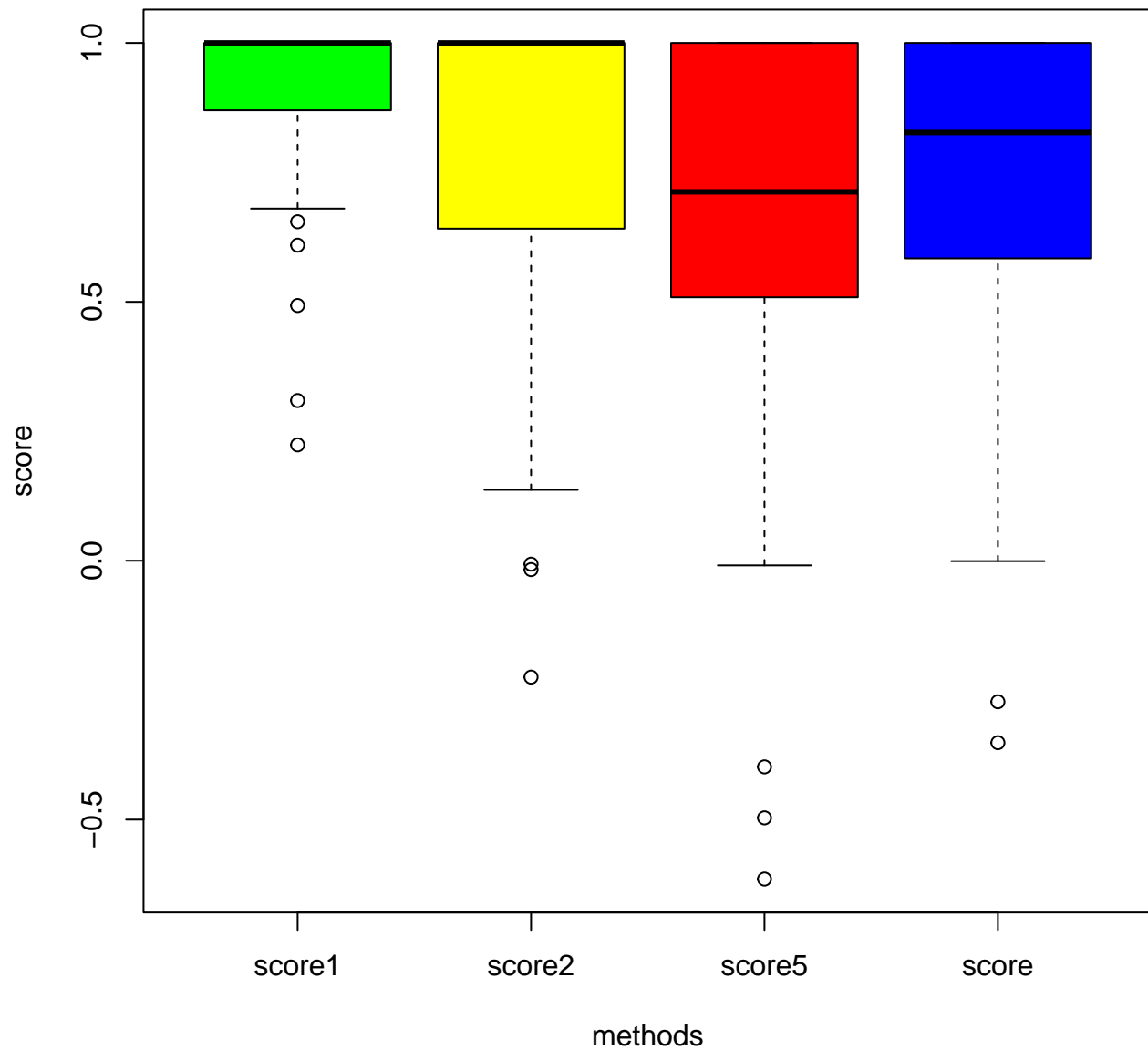

Supplement: Supplementary file 1 [file ijms-25-05267-s001.zip › File S4/2_stability_scores_boxplot/ovarian cancer_StabBoxplot.pdf]

# pancreatic cancer\_stability analysis

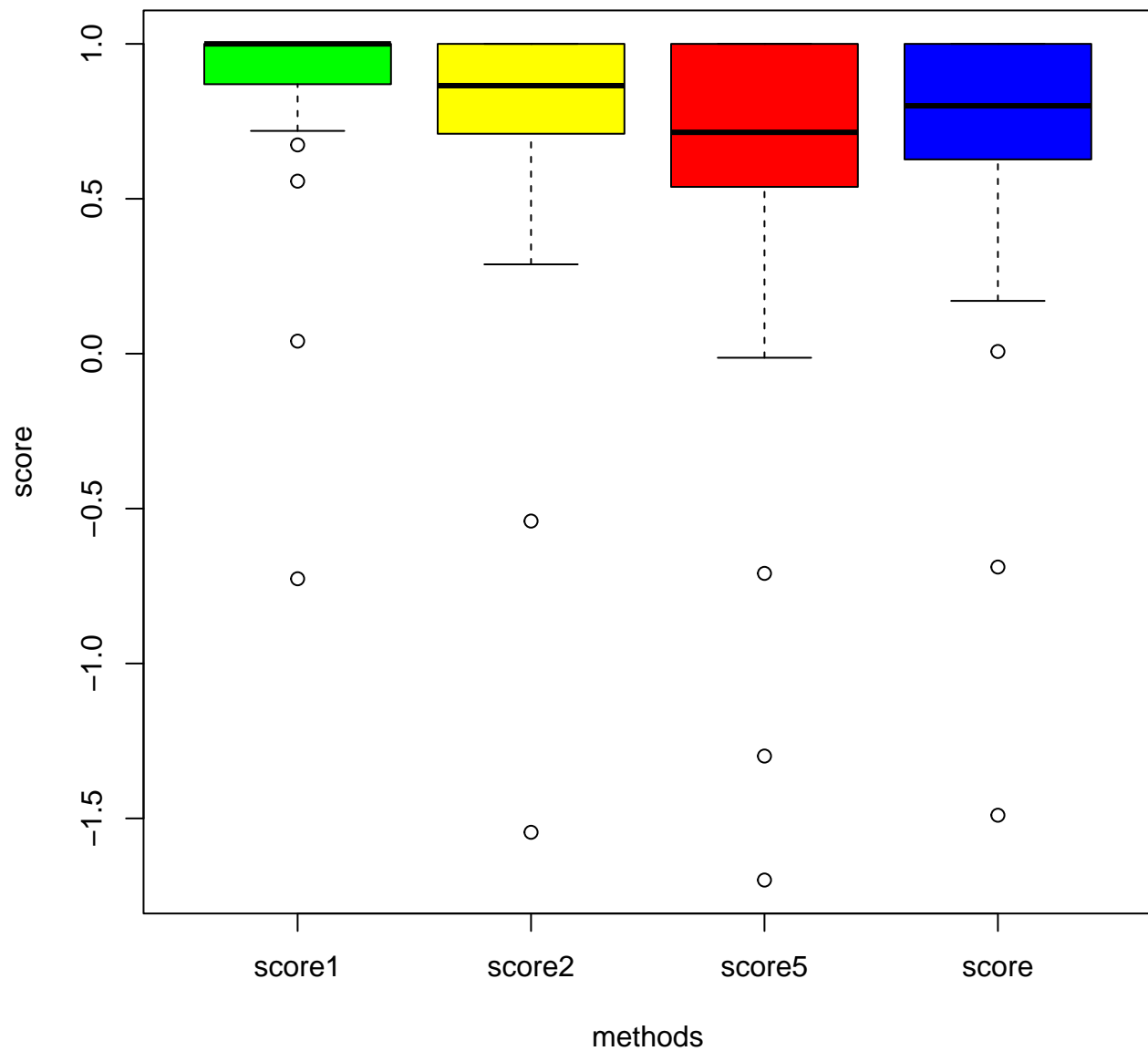

Supplement: Supplementary file 1 [file ijms-25-05267-s001.zip › File S4/2_stability_scores_boxplot/pancreatic cancer_StabBoxplot.pdf]

# pancreatic carcinoma\_stability analysis

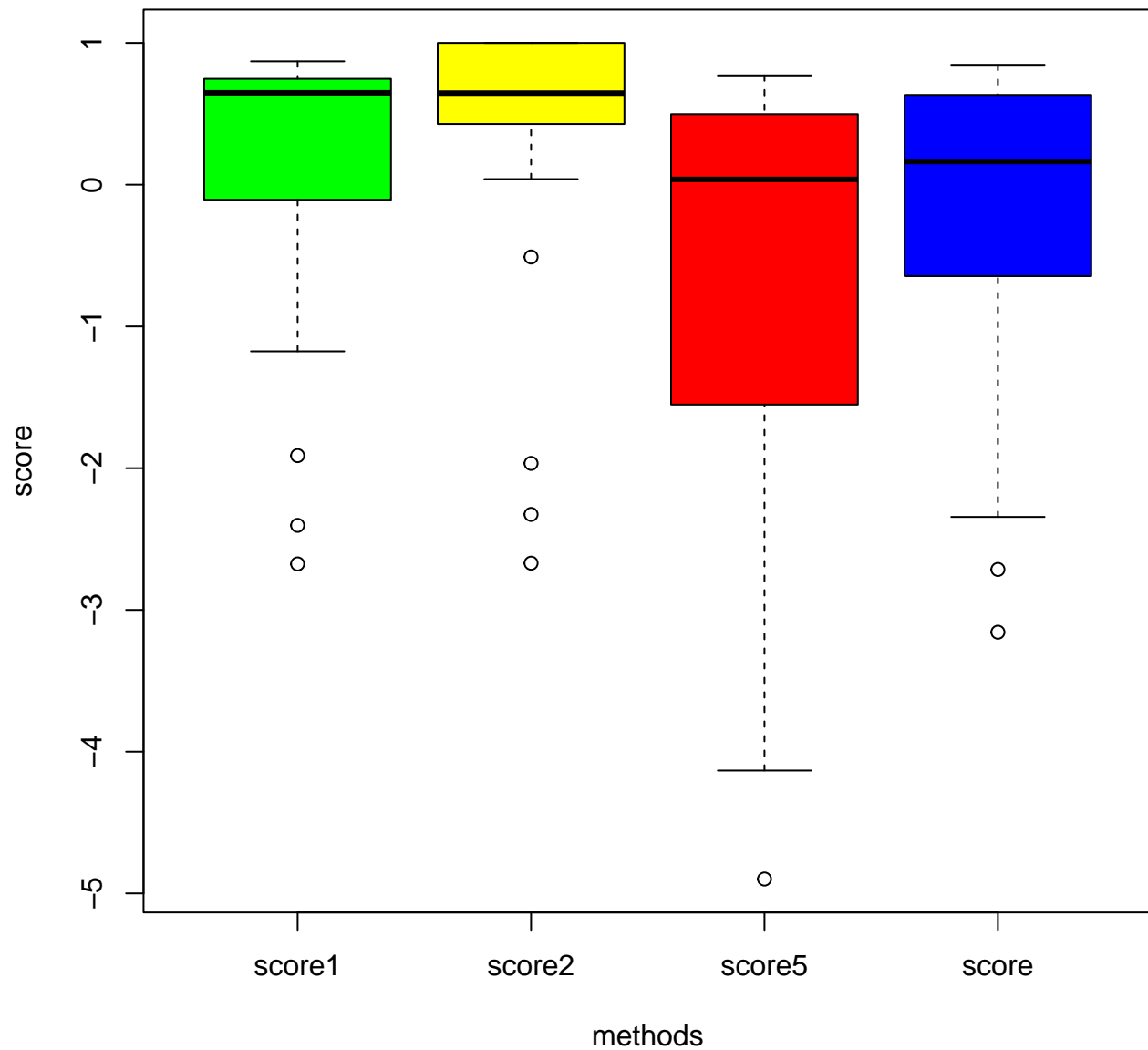

Supplement: Supplementary file 1 [file ijms-25-05267-s001.zip › File S4/2_stability_scores_boxplot/pancreatic carcinoma_StabBoxplot.pdf]

# pancreatic ductal carcinoma\_stability analysis

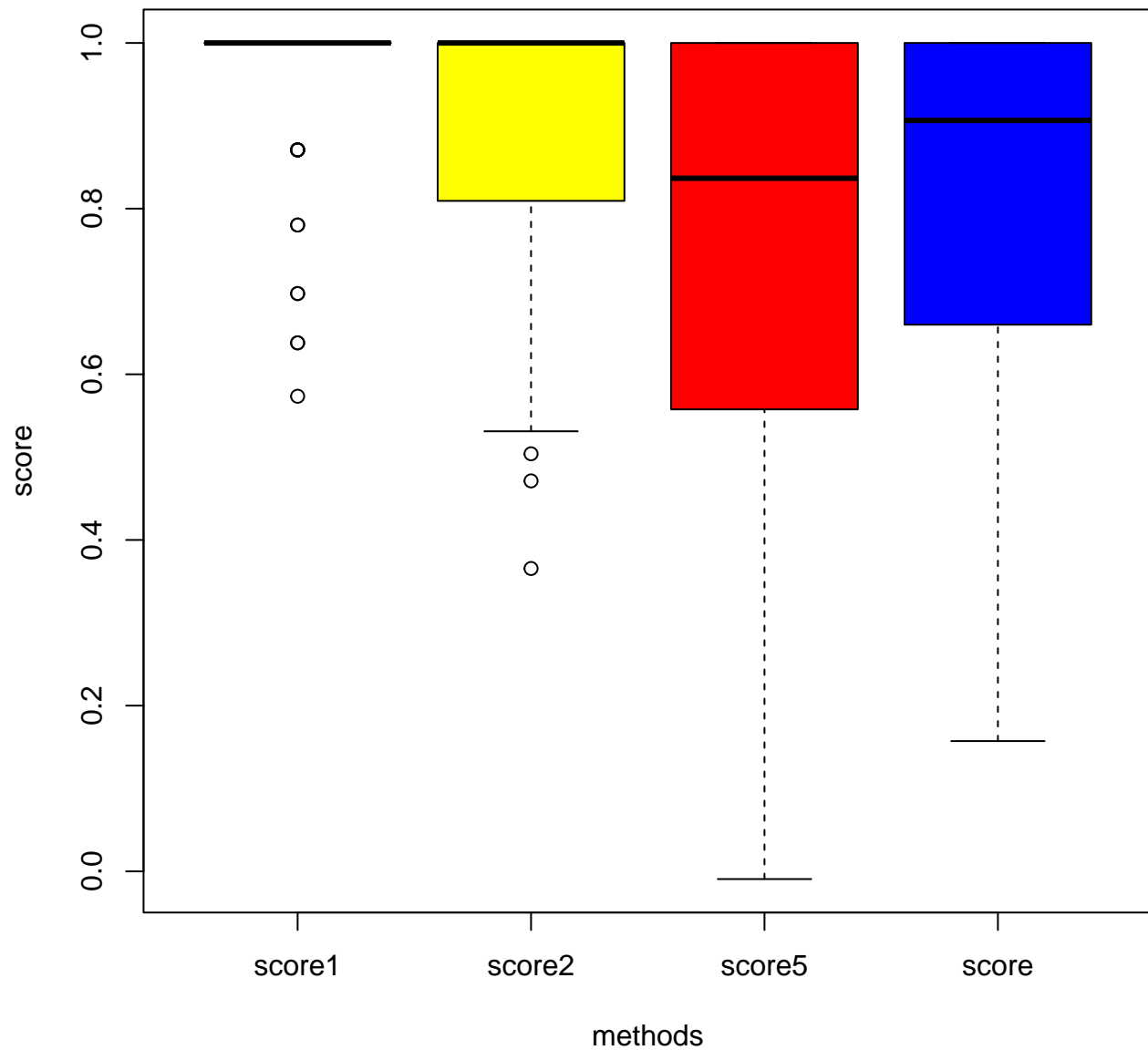

Supplement: Supplementary file 1 [file ijms-25-05267-s001.zip › File S4/2_stability_scores_boxplot/pancreatic ductal carcinoma_StabBoxplot.pdf]

# parathyroid carcinoma\_stability analysis

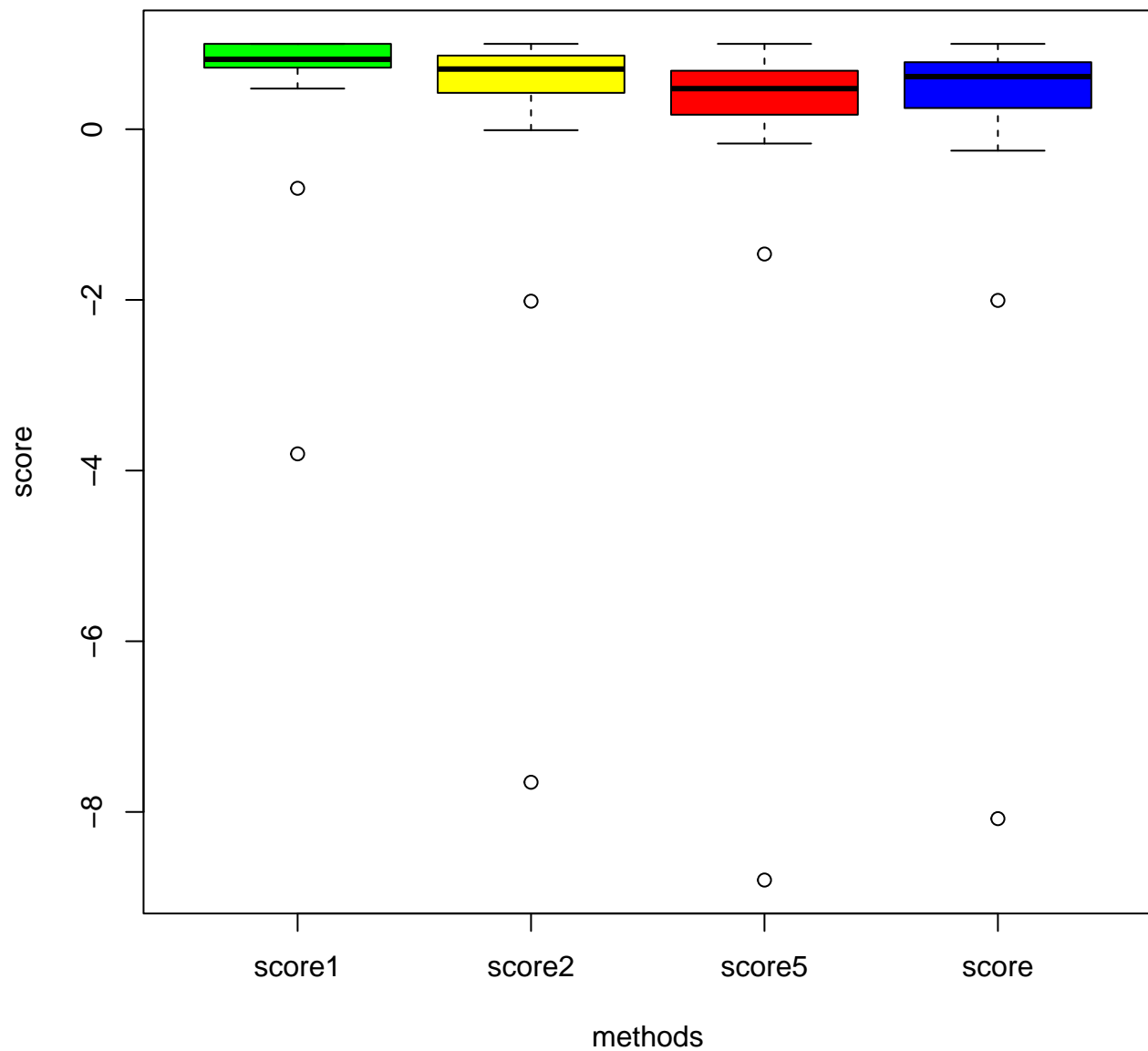

Supplement: Supplementary file 1 [file ijms-25-05267-s001.zip › File S4/2_stability_scores_boxplot/parathyroid carcinoma_StabBoxplot.pdf]

# penile cancer\_stability analysis

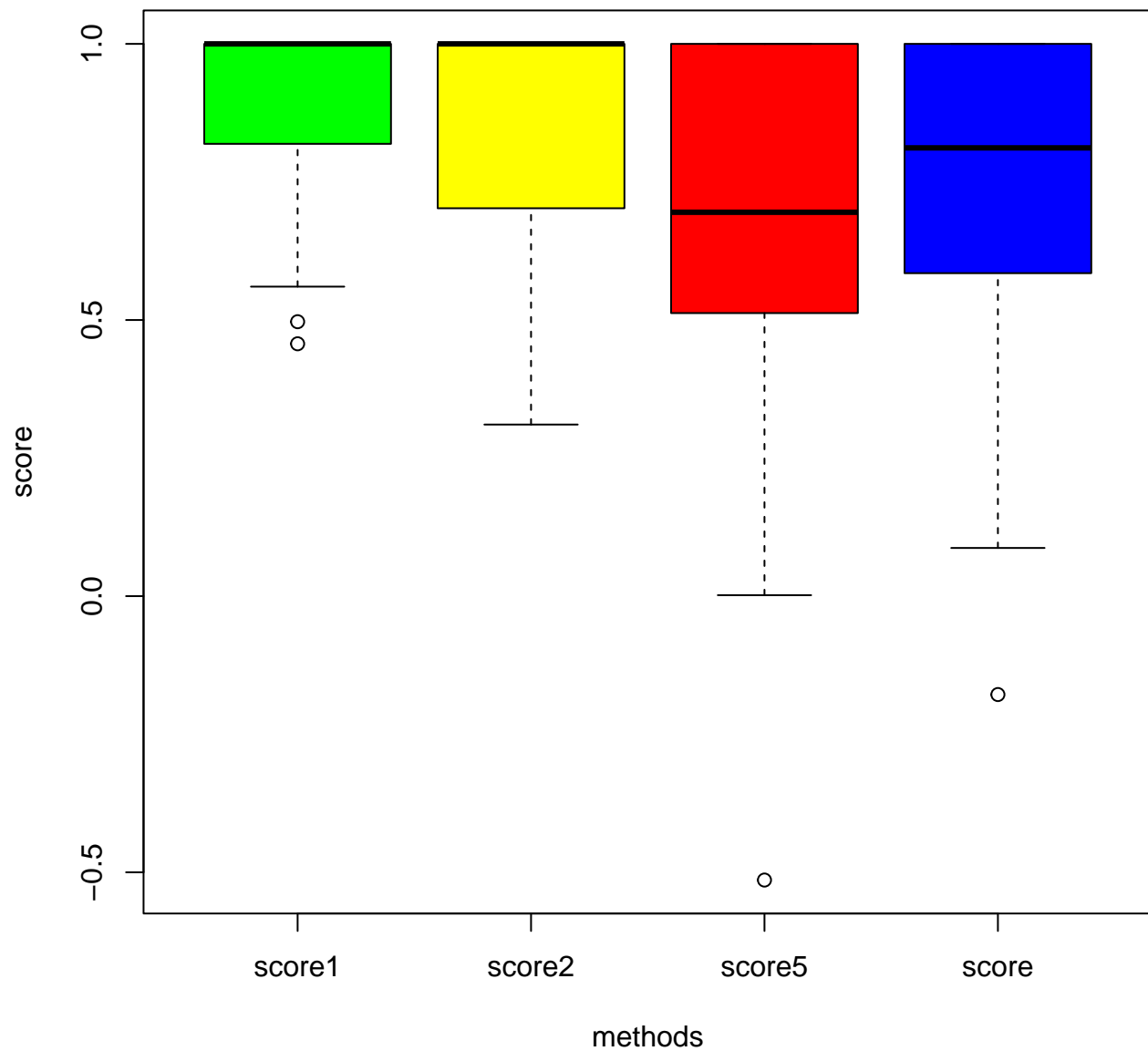

Supplement: Supplementary file 1 [file ijms-25-05267-s001.zip › File S4/2_stability_scores_boxplot/penile cancer_StabBoxplot.pdf]

# pituitary cancer\_stability analysis

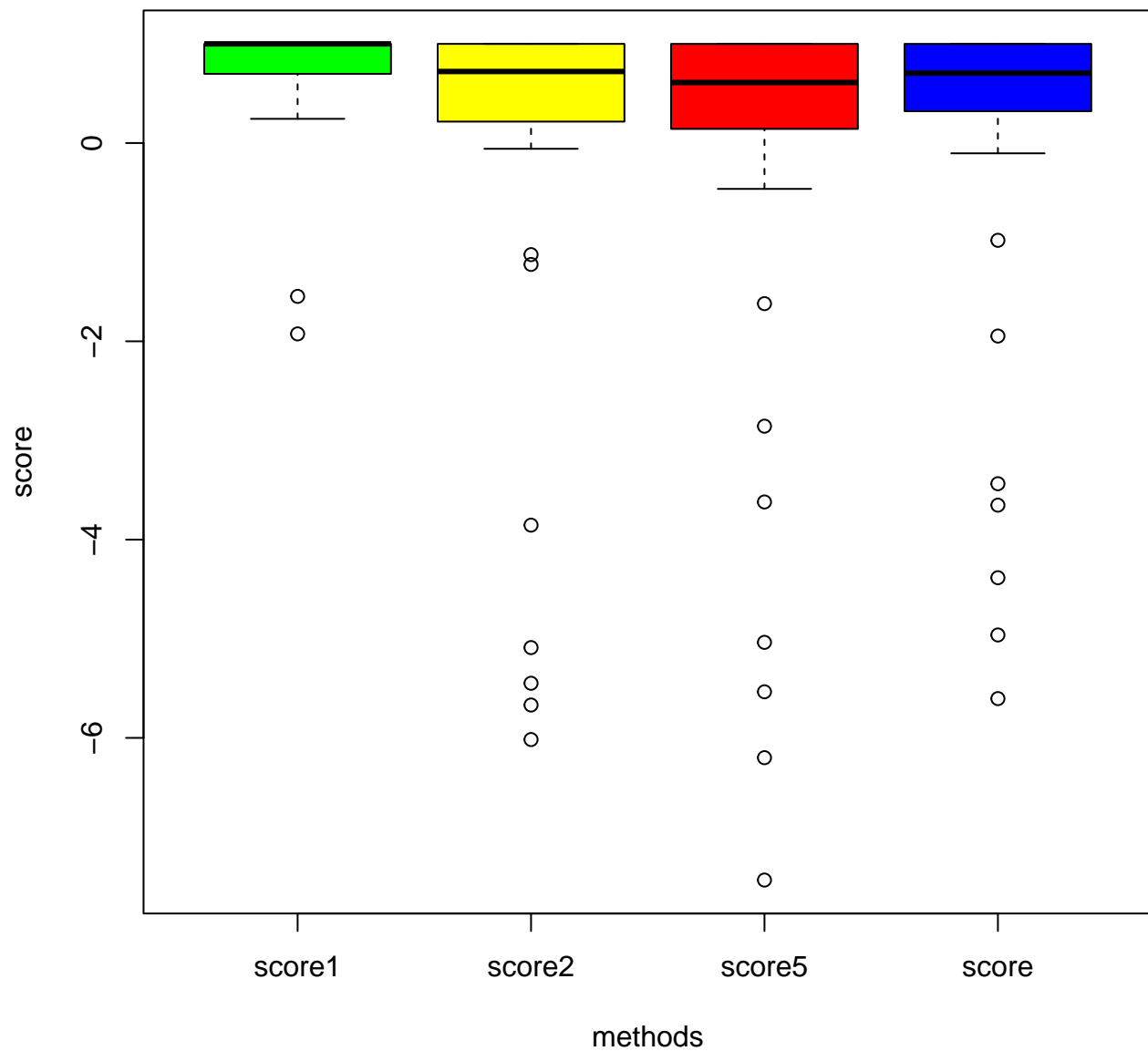

Supplement: Supplementary file 1 [file ijms-25-05267-s001.zip › File S4/2_stability_scores_boxplot/pituitary cancer_StabBoxplot.pdf]

# pleural cancer\_stability analysis

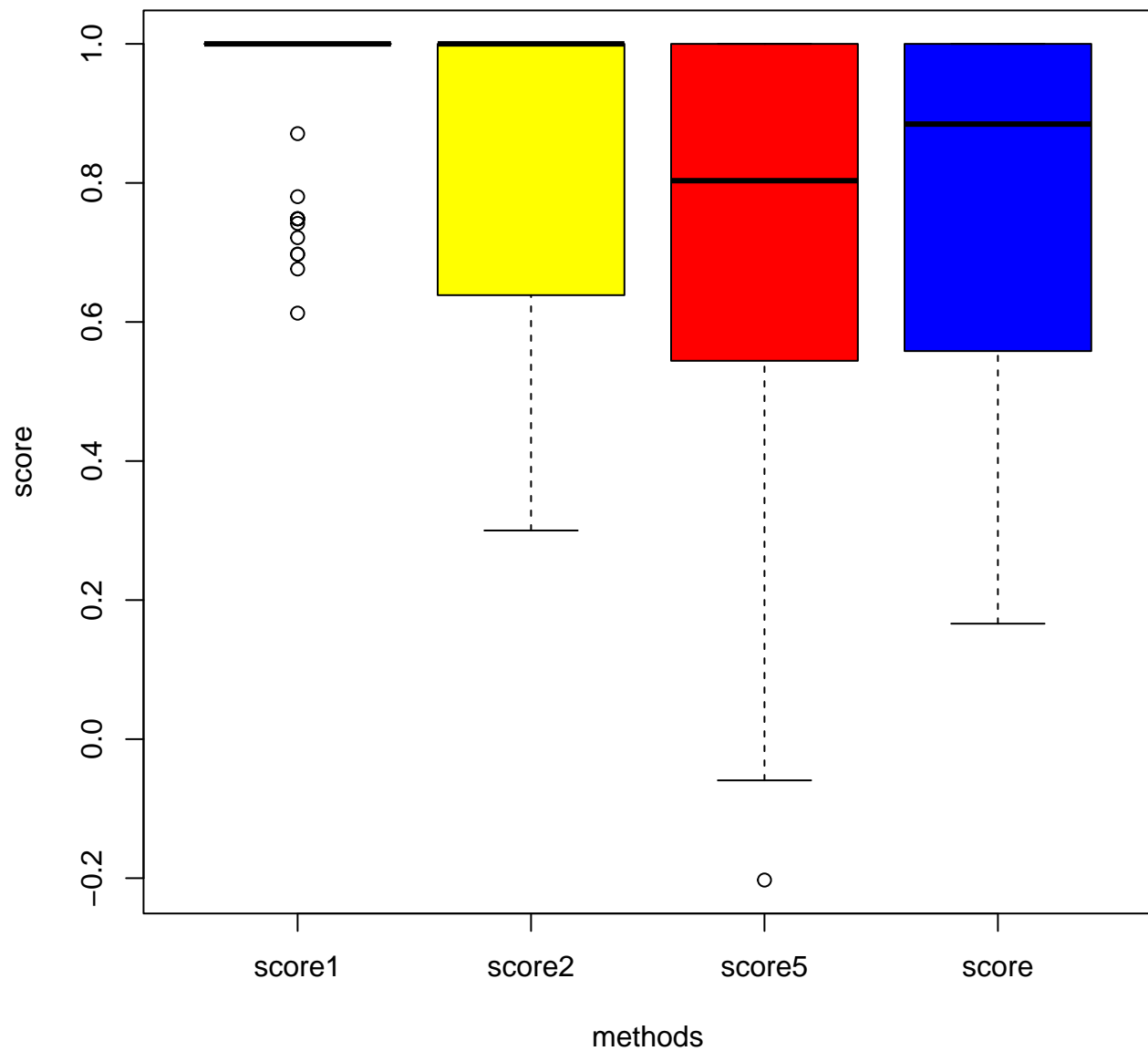

Supplement: Supplementary file 1 [file ijms-25-05267-s001.zip › File S4/2_stability_scores_boxplot/pleural cancer_StabBoxplot.pdf]

# prolactinoma\_stability analysis

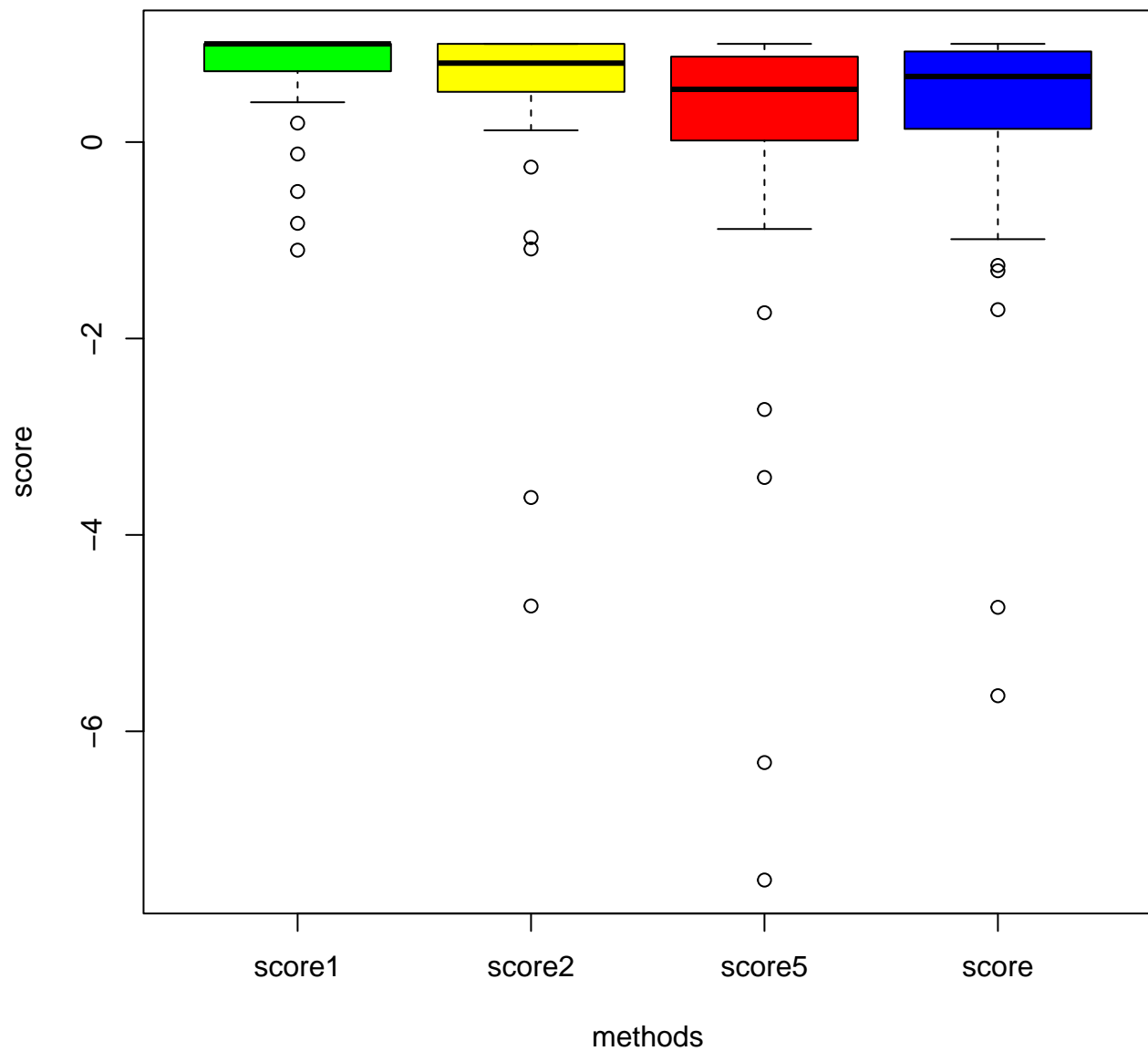

Supplement: Supplementary file 1 [file ijms-25-05267-s001.zip › File S4/2_stability_scores_boxplot/prolactinoma_StabBoxplot.pdf]

# rectal neoplasm\_stability analysis

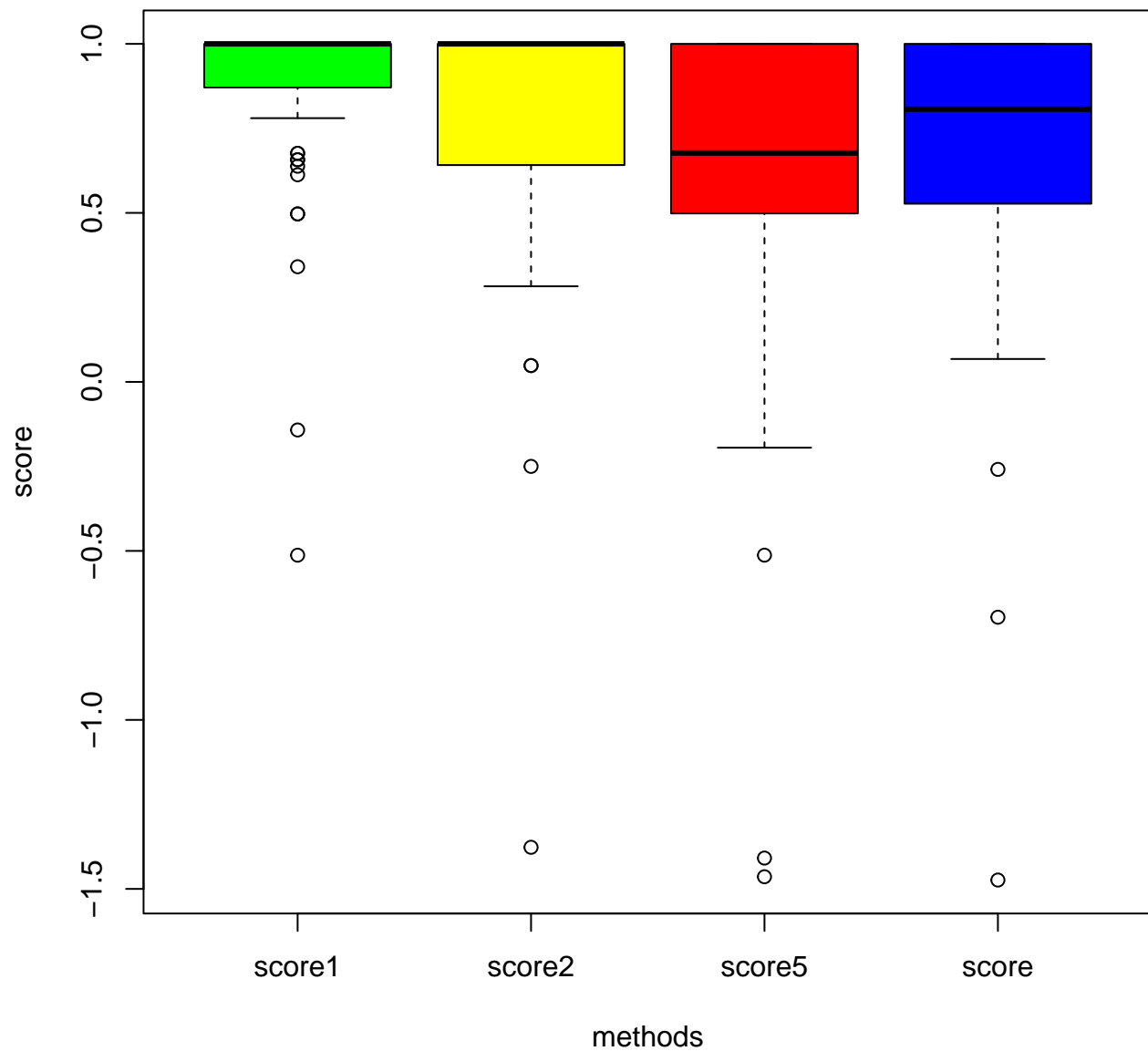

Supplement: Supplementary file 1 [file ijms-25-05267-s001.zip › File S4/2_stability_scores_boxplot/rectal neoplasm_StabBoxplot.pdf]

# renal cell carcinoma\_stability analysis

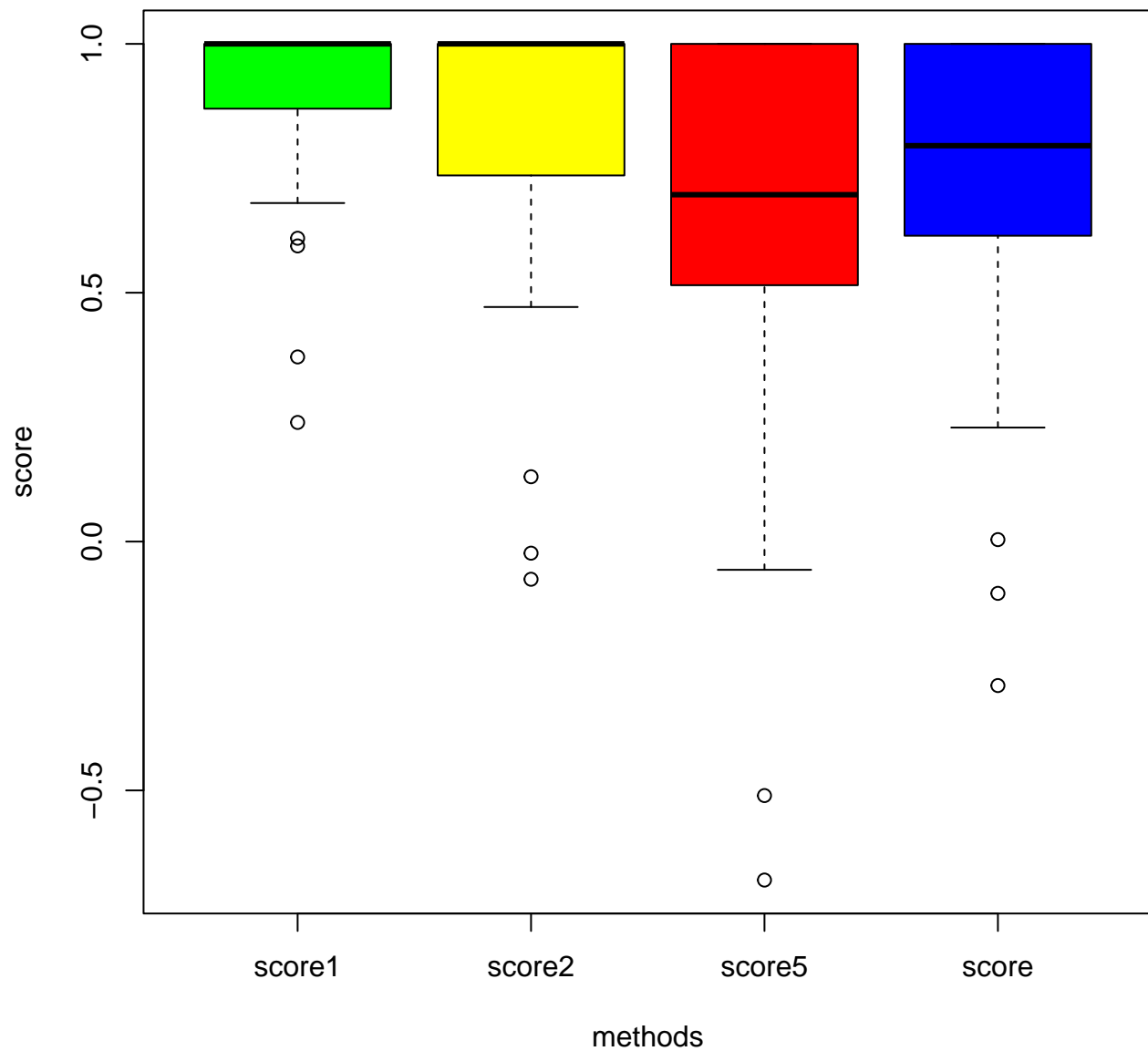

Supplement: Supplementary file 1 [file ijms-25-05267-s001.zip › File S4/2_stability_scores_boxplot/renal cell carcinoma_StabBoxplot.pdf]

# skin benign neoplasm\_stability analysis

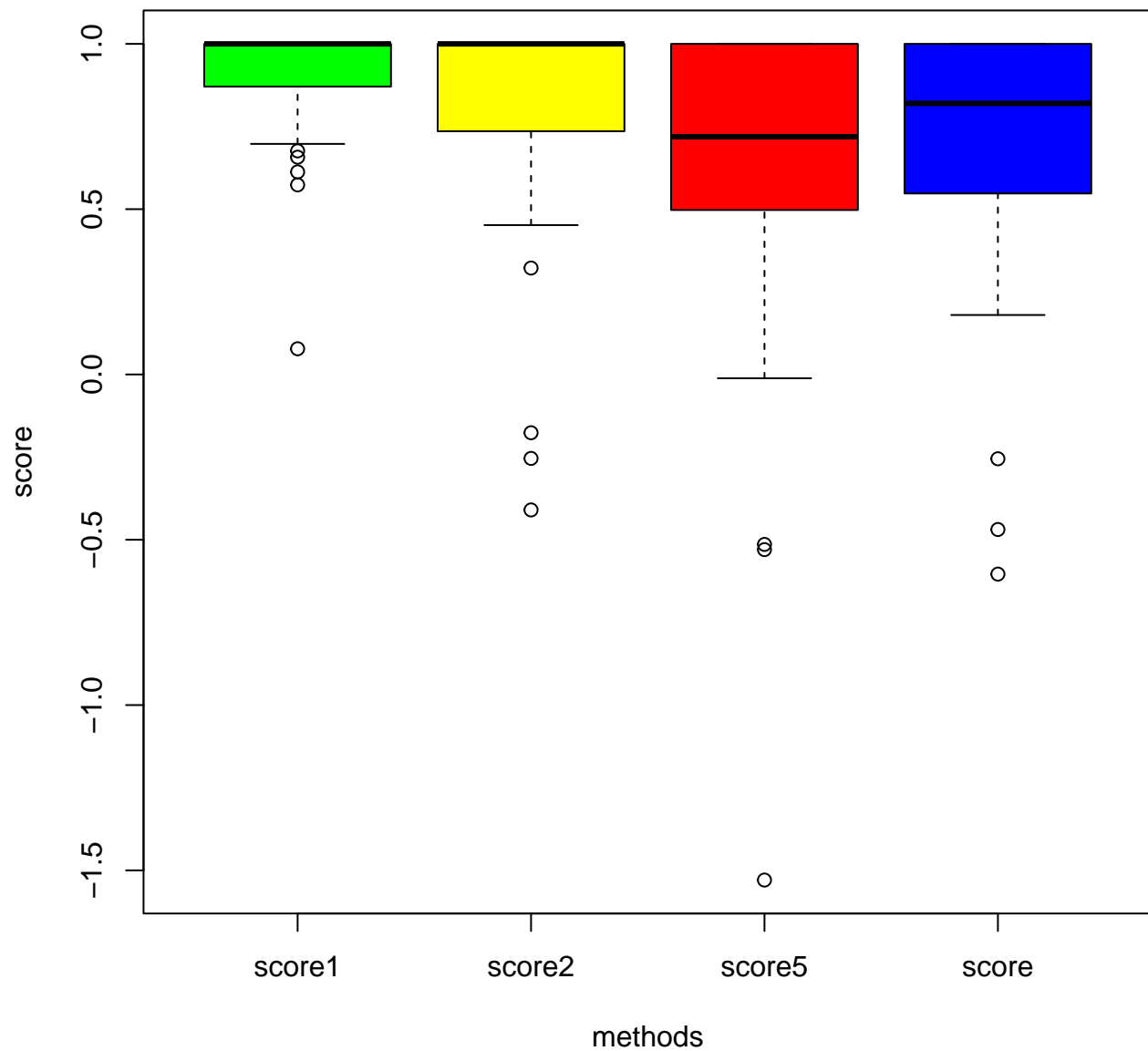

Supplement: Supplementary file 1 [file ijms-25-05267-s001.zip › File S4/2_stability_scores_boxplot/skin benign neoplasm_StabBoxplot.pdf]

# skin melanoma\_stability analysis

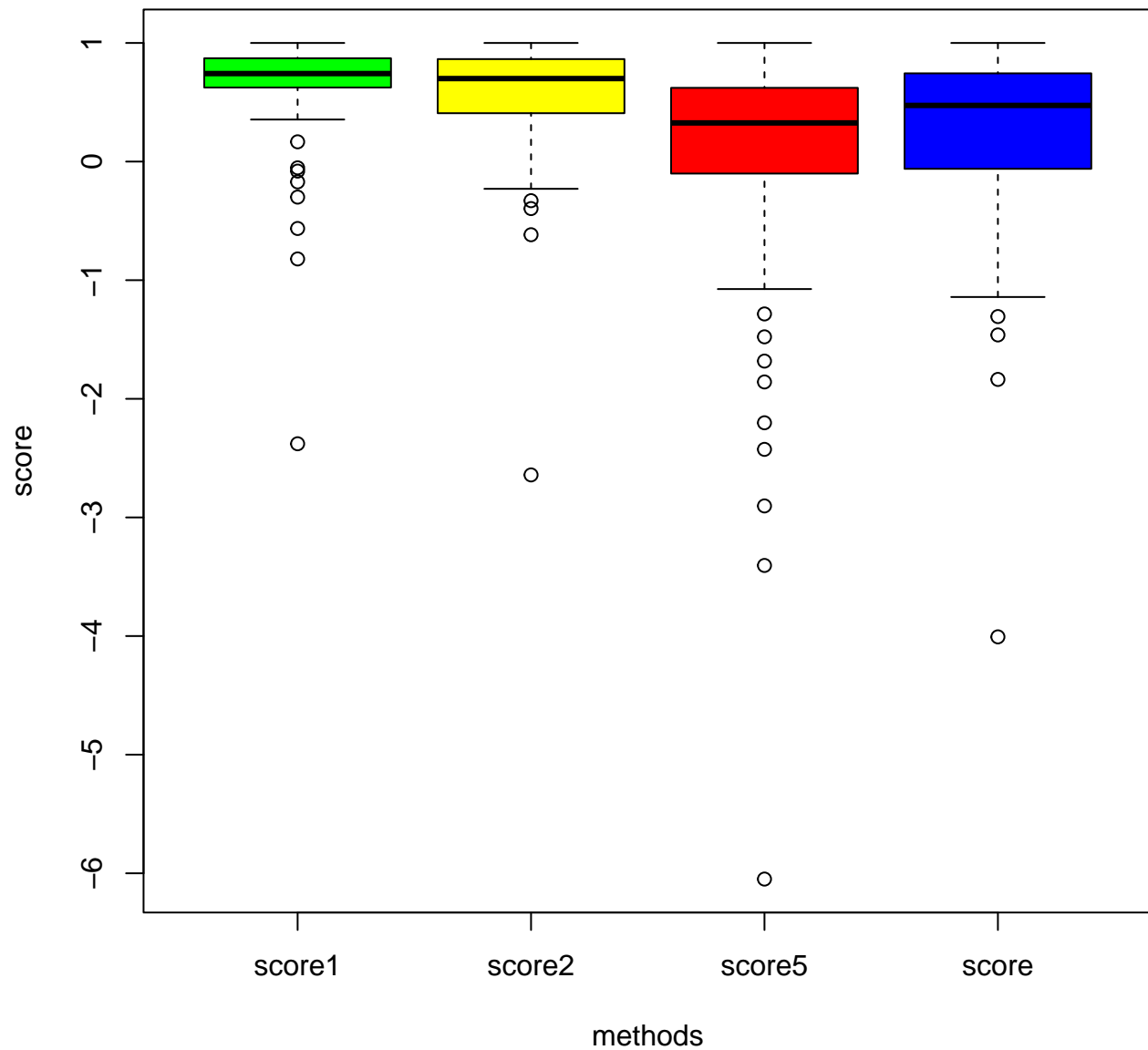

Supplement: Supplementary file 1 [file ijms-25-05267-s001.zip › File S4/2_stability_scores_boxplot/skin melanoma_StabBoxplot.pdf]

# stomach cancer\_stability analysis

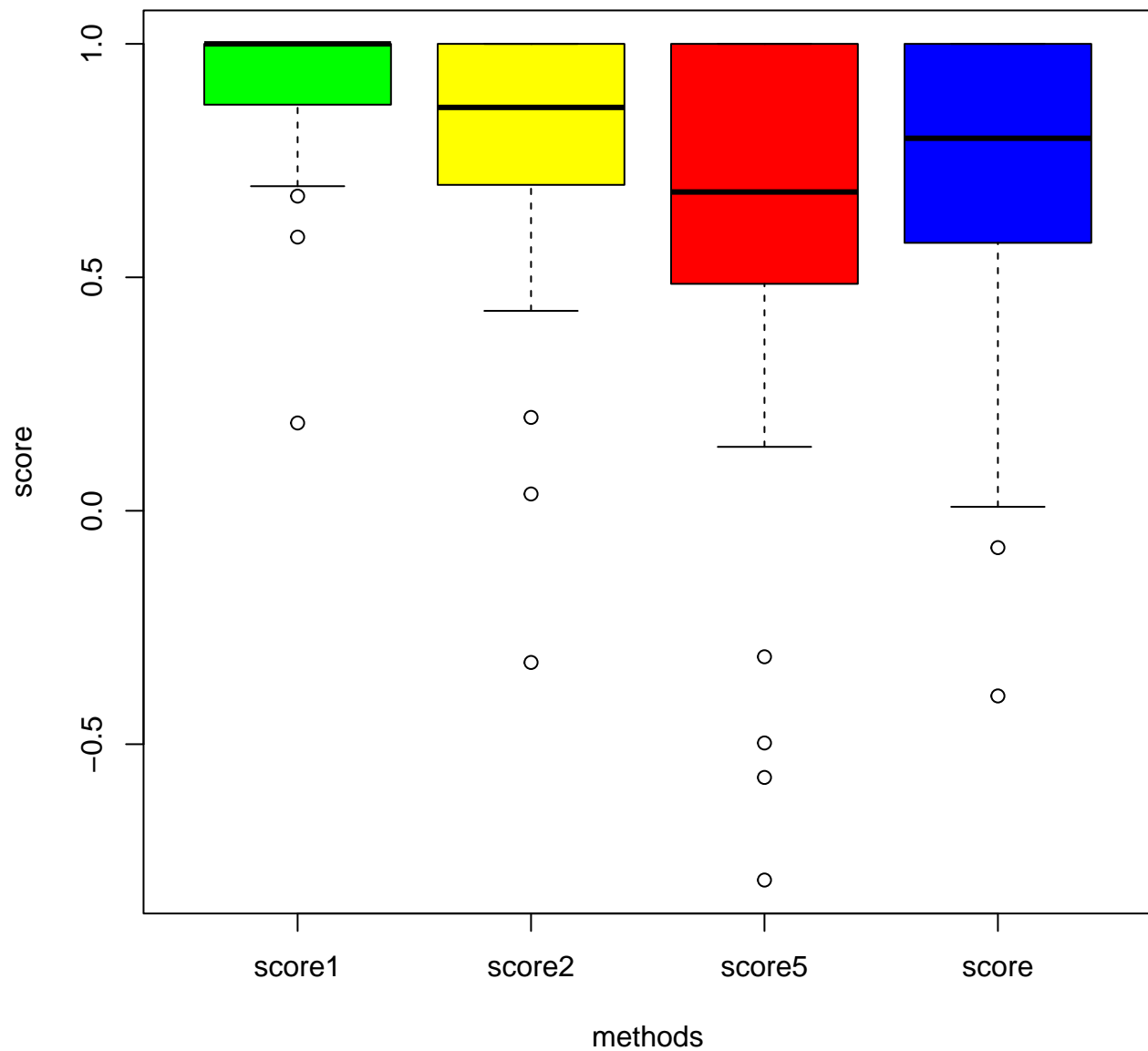

Supplement: Supplementary file 1 [file ijms-25-05267-s001.zip › File S4/2_stability_scores_boxplot/stomach cancer_StabBoxplot.pdf]

# supratentorial cancer\_stability analysis

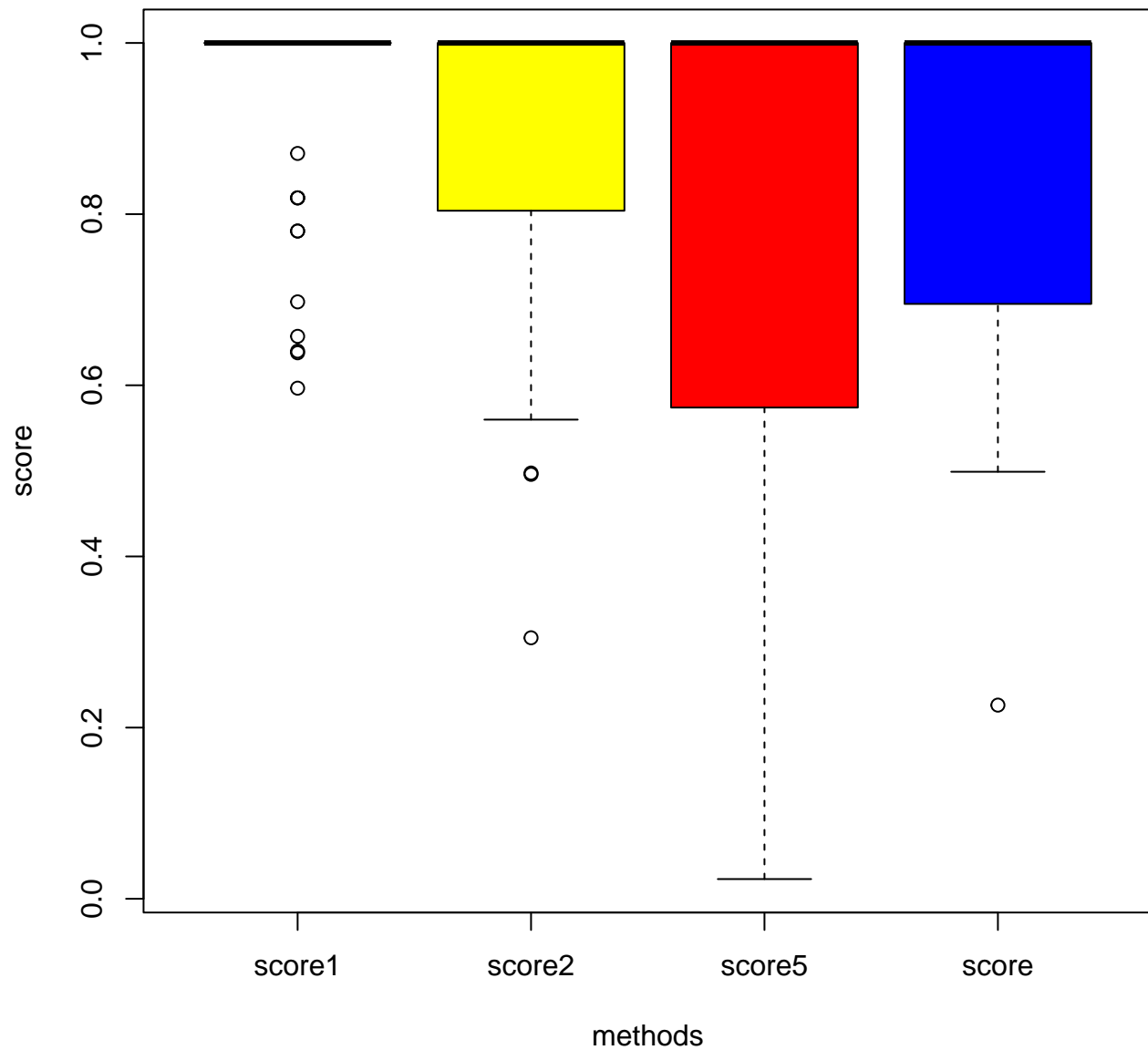

Supplement: Supplementary file 1 [file ijms-25-05267-s001.zip › File S4/2_stability_scores_boxplot/supratentorial cancer_StabBoxplot.pdf]

# testicular cancer\_stability analysis

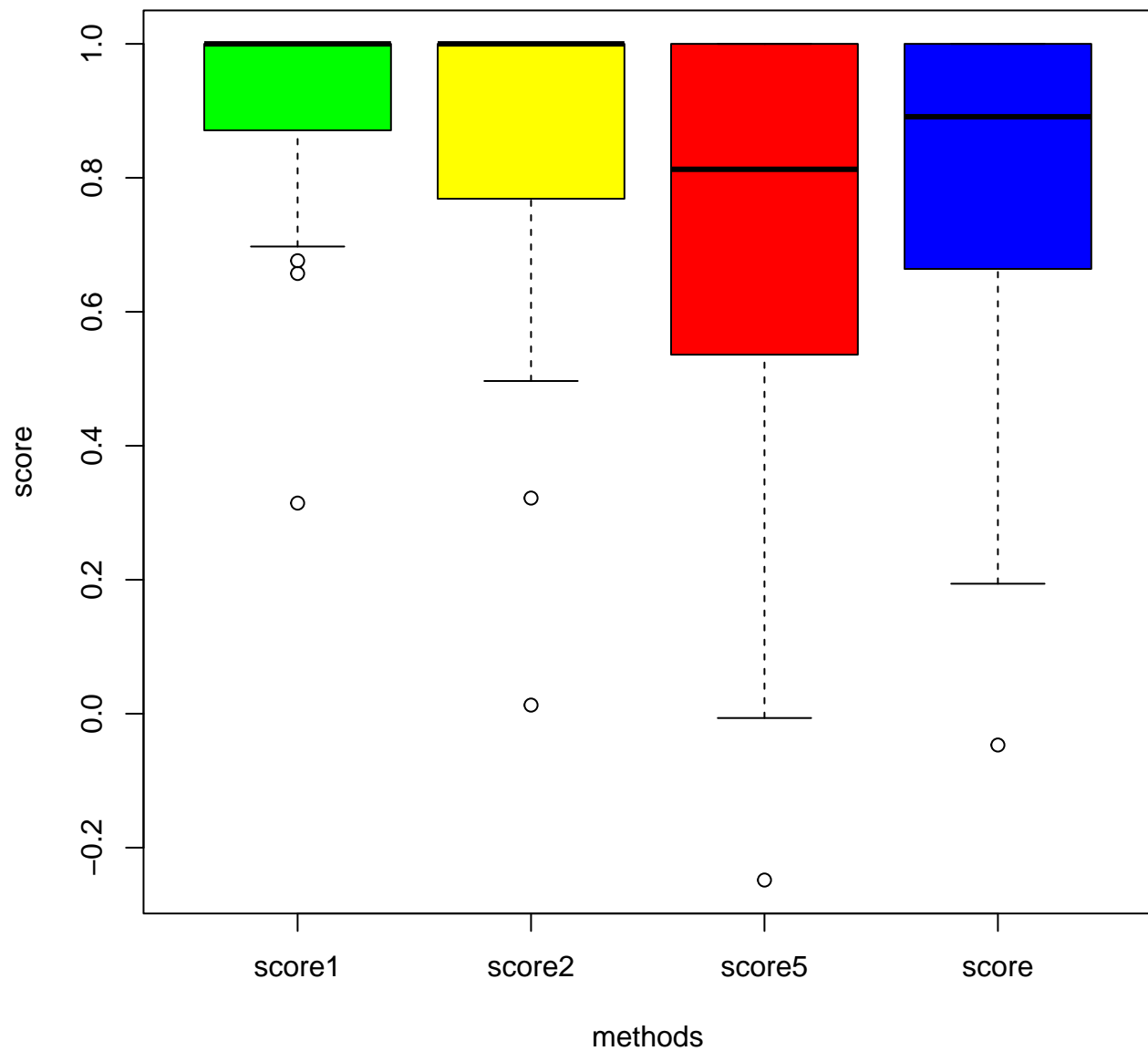

Supplement: Supplementary file 1 [file ijms-25-05267-s001.zip › File S4/2_stability_scores_boxplot/testicular cancer_StabBoxplot.pdf]

# thymoma\_stability analysis

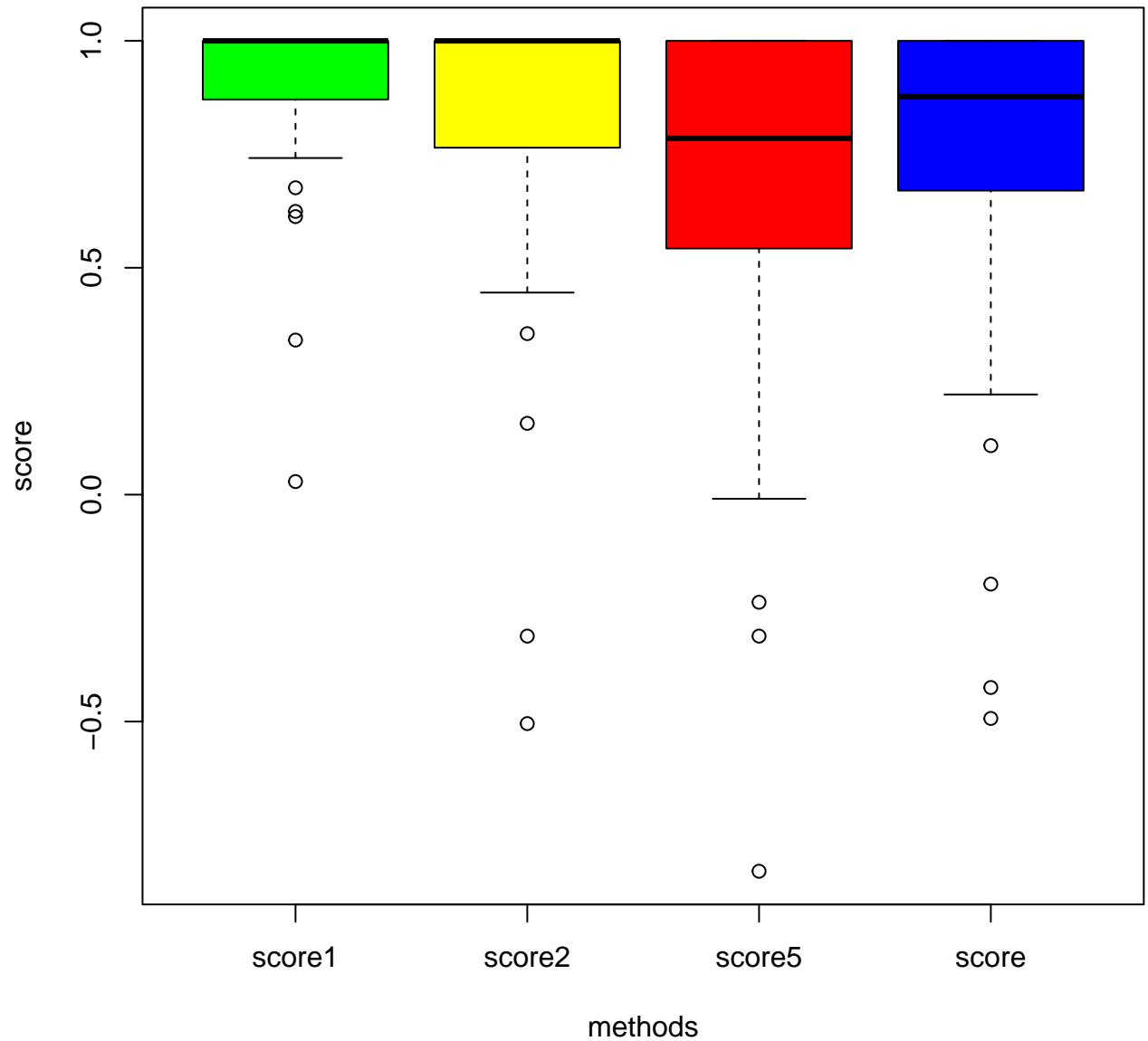

Supplement: Supplementary file 1 [file ijms-25-05267-s001.zip › File S4/2_stability_scores_boxplot/thymoma_StabBoxplot.pdf]

# thymus cancer\_stability analysis

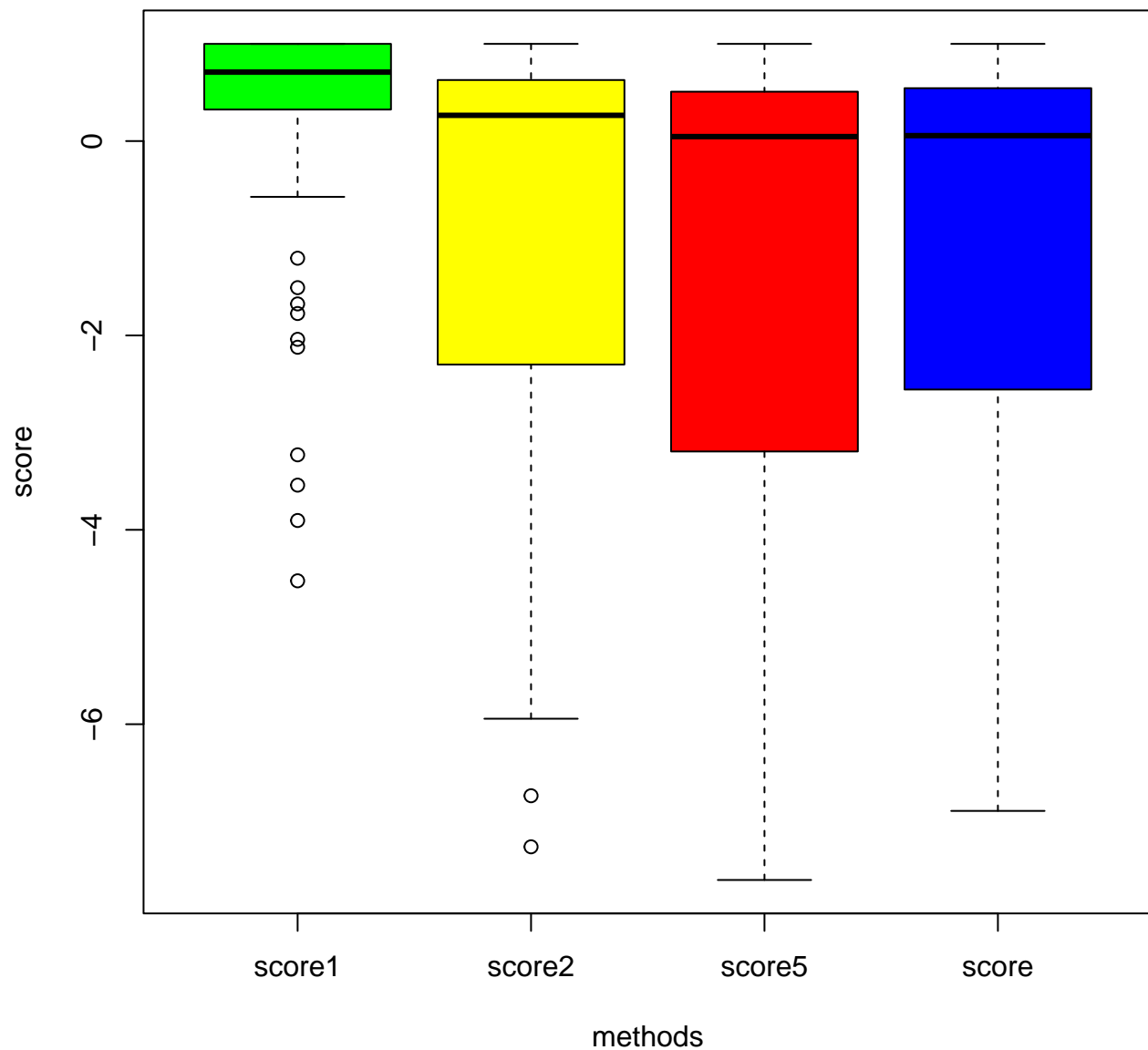

Supplement: Supplementary file 1 [file ijms-25-05267-s001.zip › File S4/2_stability_scores_boxplot/thymus cancer_StabBoxplot.pdf]

# thyroid cancer\_stability analysis

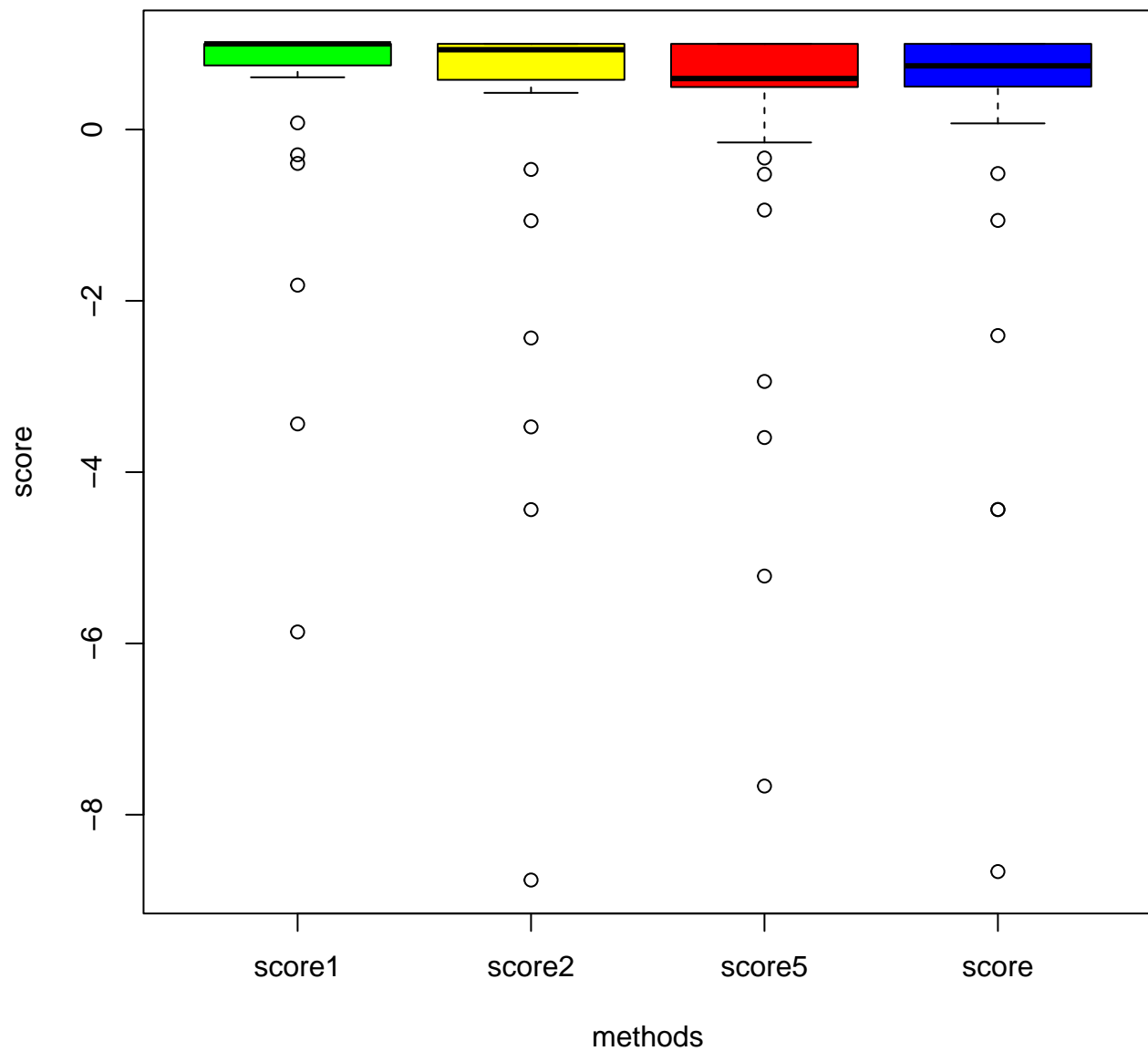

Supplement: Supplementary file 1 [file ijms-25-05267-s001.zip › File S4/2_stability_scores_boxplot/thyroid cancer_StabBoxplot.pdf]

# thyroid medullary carcinoma\_stability analysis

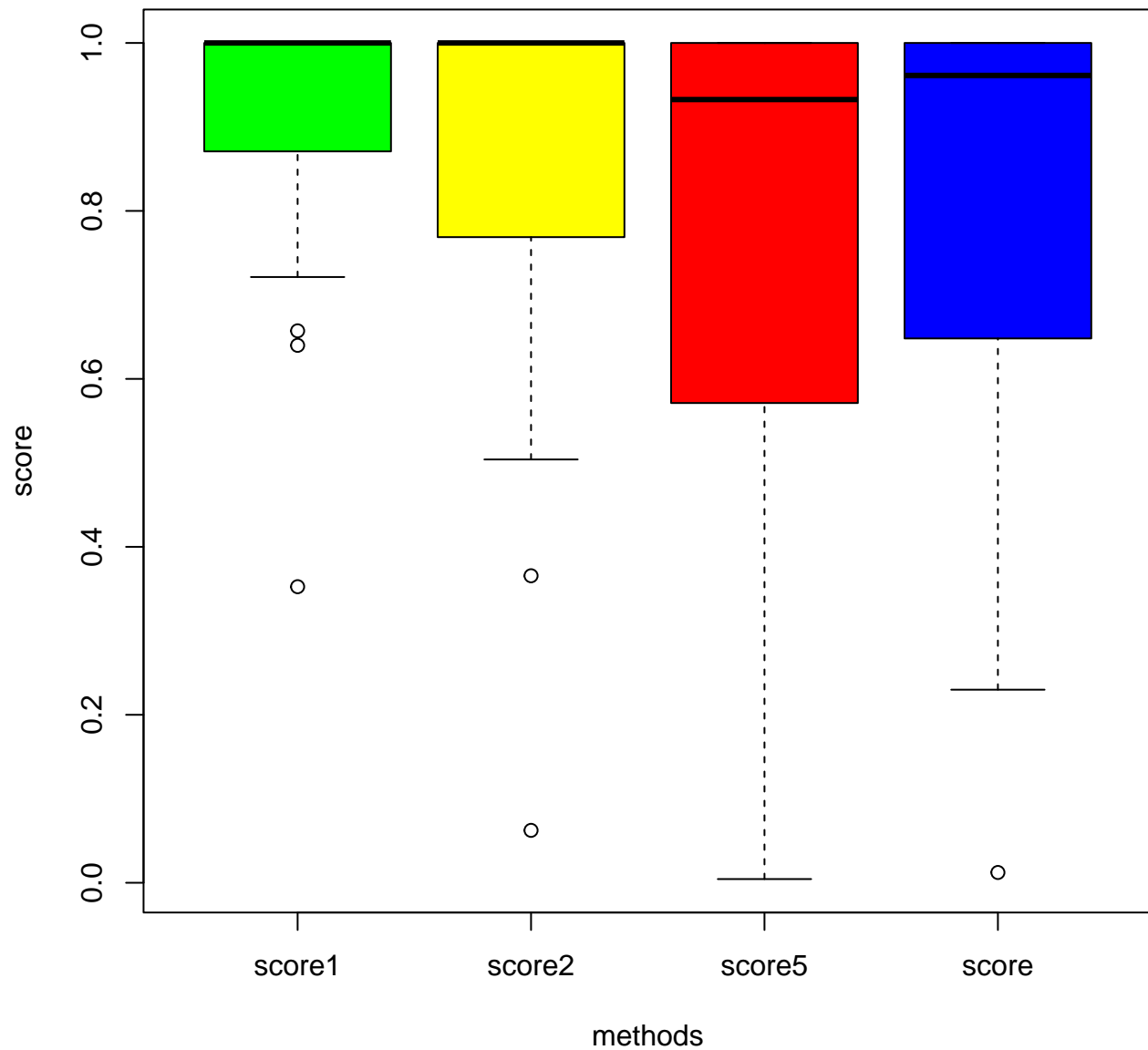

Supplement: Supplementary file 1 [file ijms-25-05267-s001.zip › File S4/2_stability_scores_boxplot/thyroid medullary carcinoma_StabBoxplot.pdf]

# tongue cancer\_stability analysis

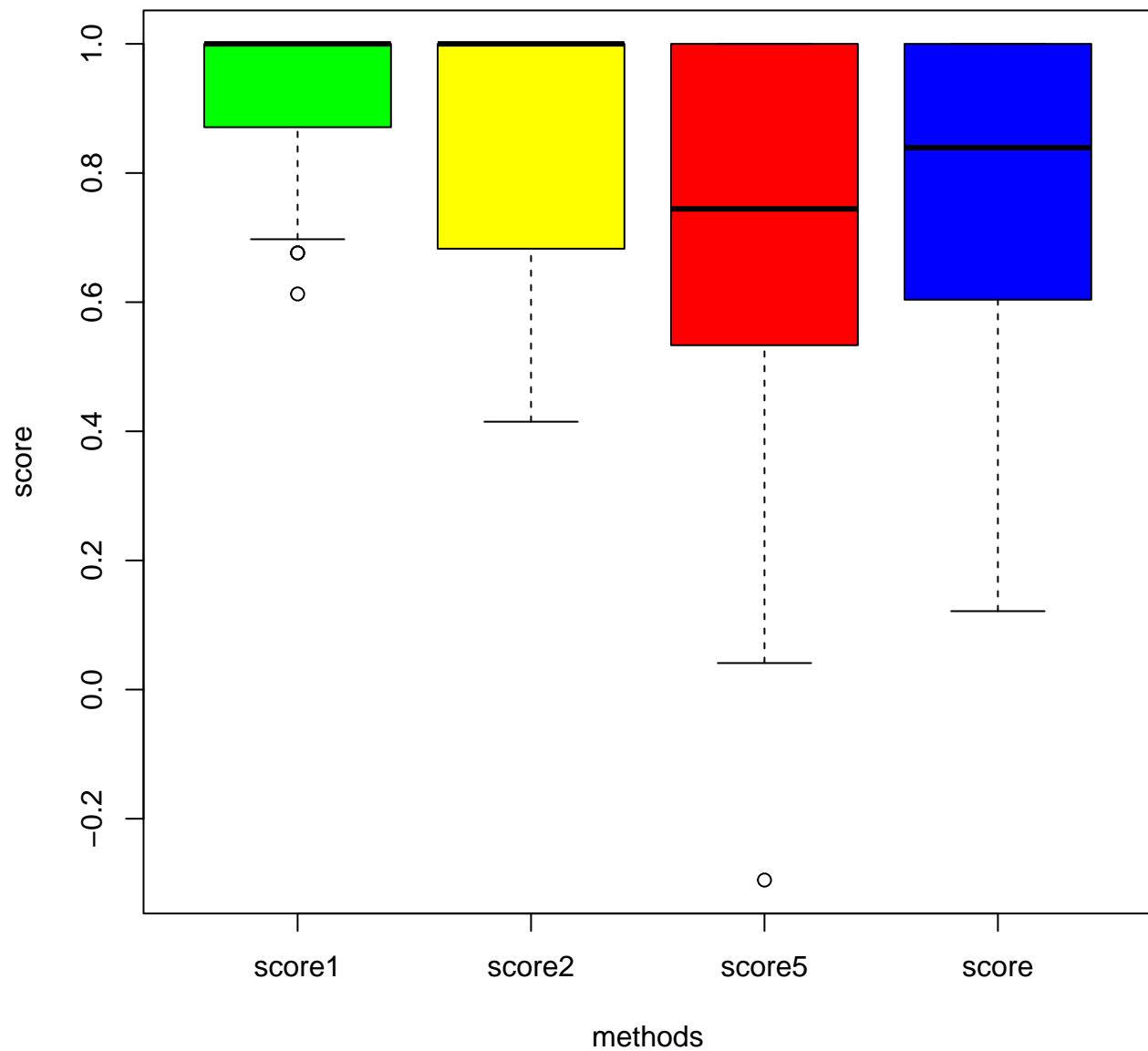

Supplement: Supplementary file 1 [file ijms-25-05267-s001.zip › File S4/2_stability_scores_boxplot/tongue cancer_StabBoxplot.pdf]

# urinary bladder cancer\_stability analysis

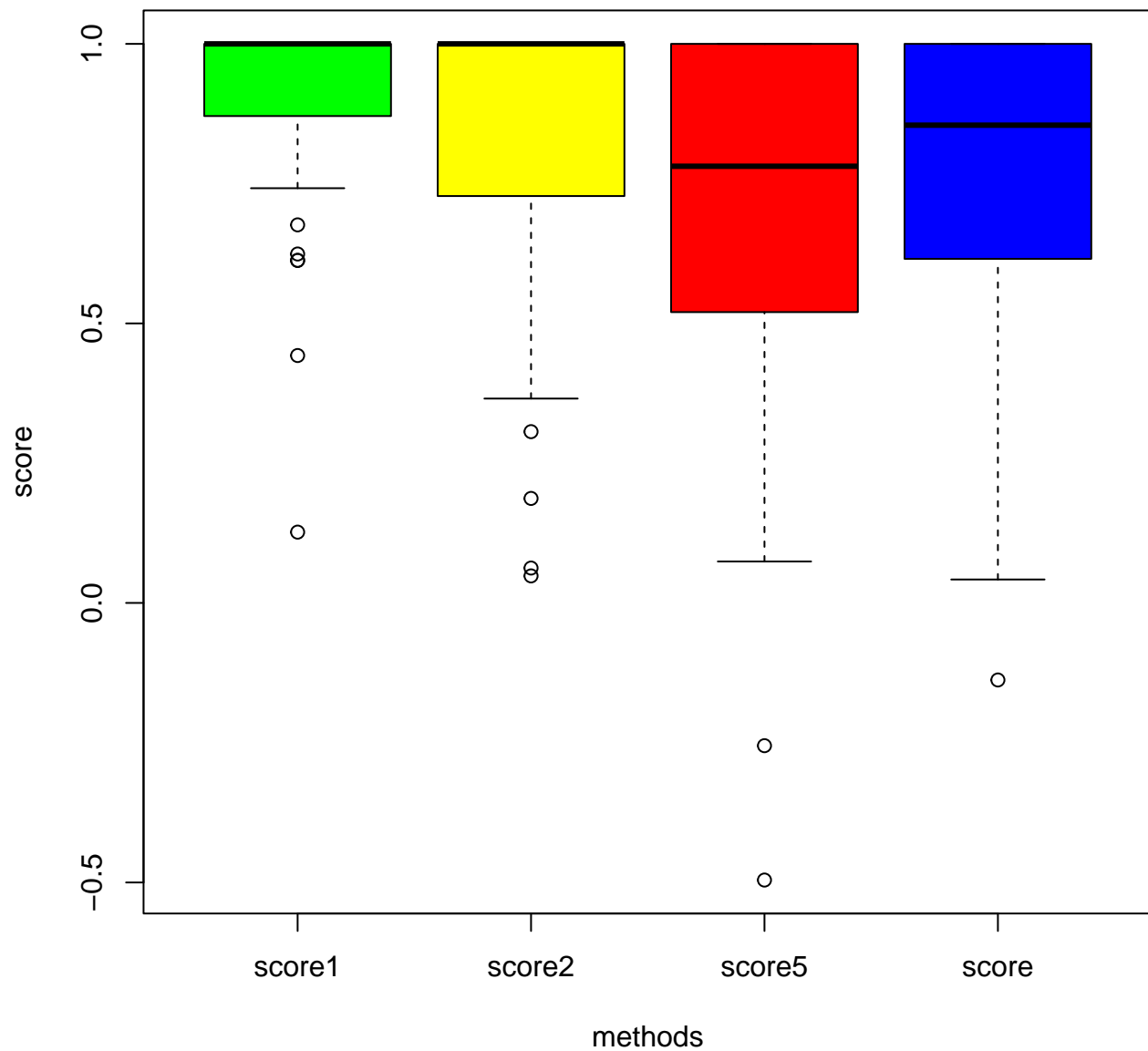

Supplement: Supplementary file 1 [file ijms-25-05267-s001.zip › File S4/2_stability_scores_boxplot/urinary bladder cancer_StabBoxplot.pdf]

# urinary system benign neoplasm\_stability analysis

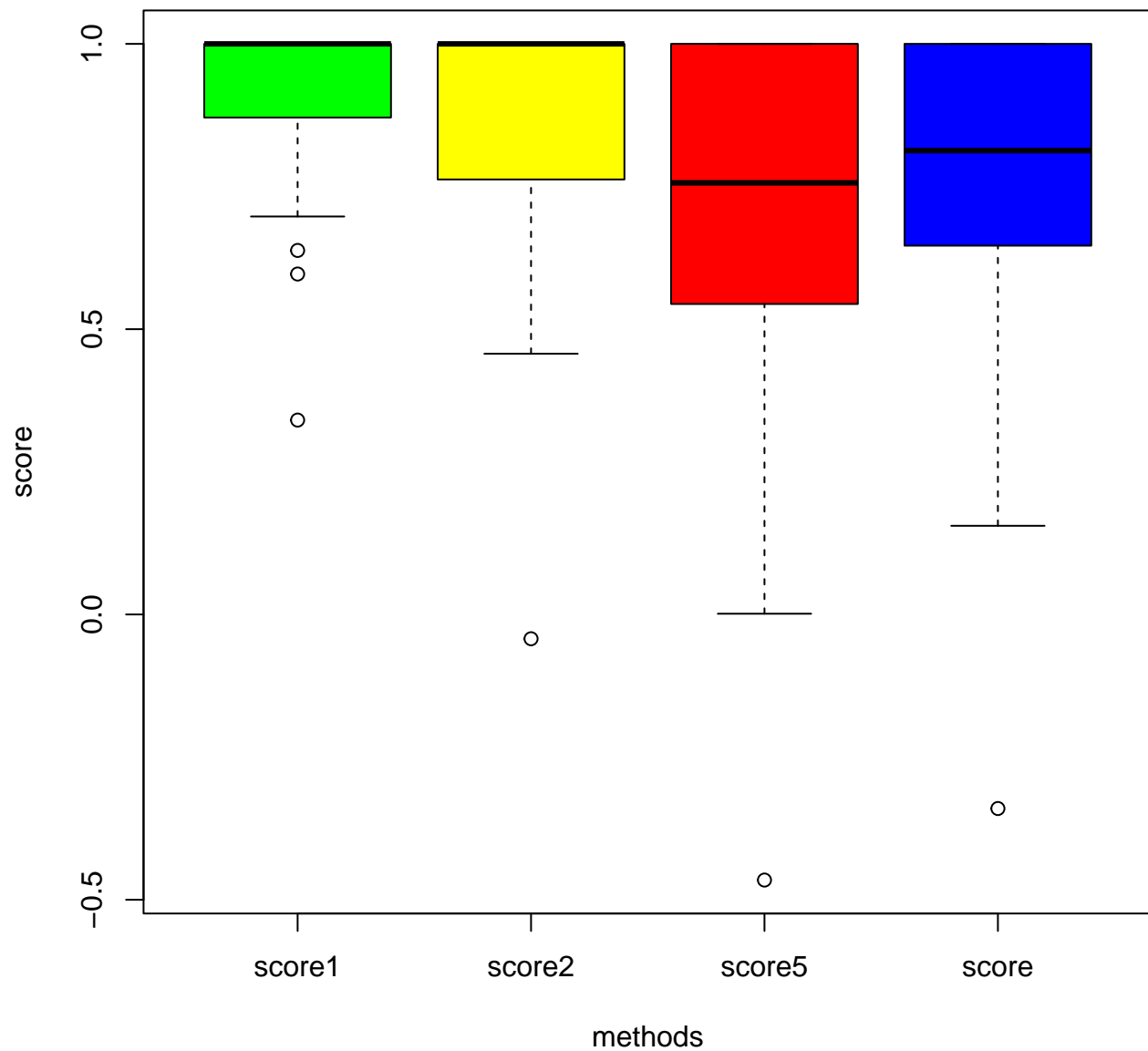

Supplement: Supplementary file 1 [file ijms-25-05267-s001.zip › File S4/2_stability_scores_boxplot/urinary system benign neoplasm_StabBoxplot.pdf]

# uterine cancer\_stability analysis

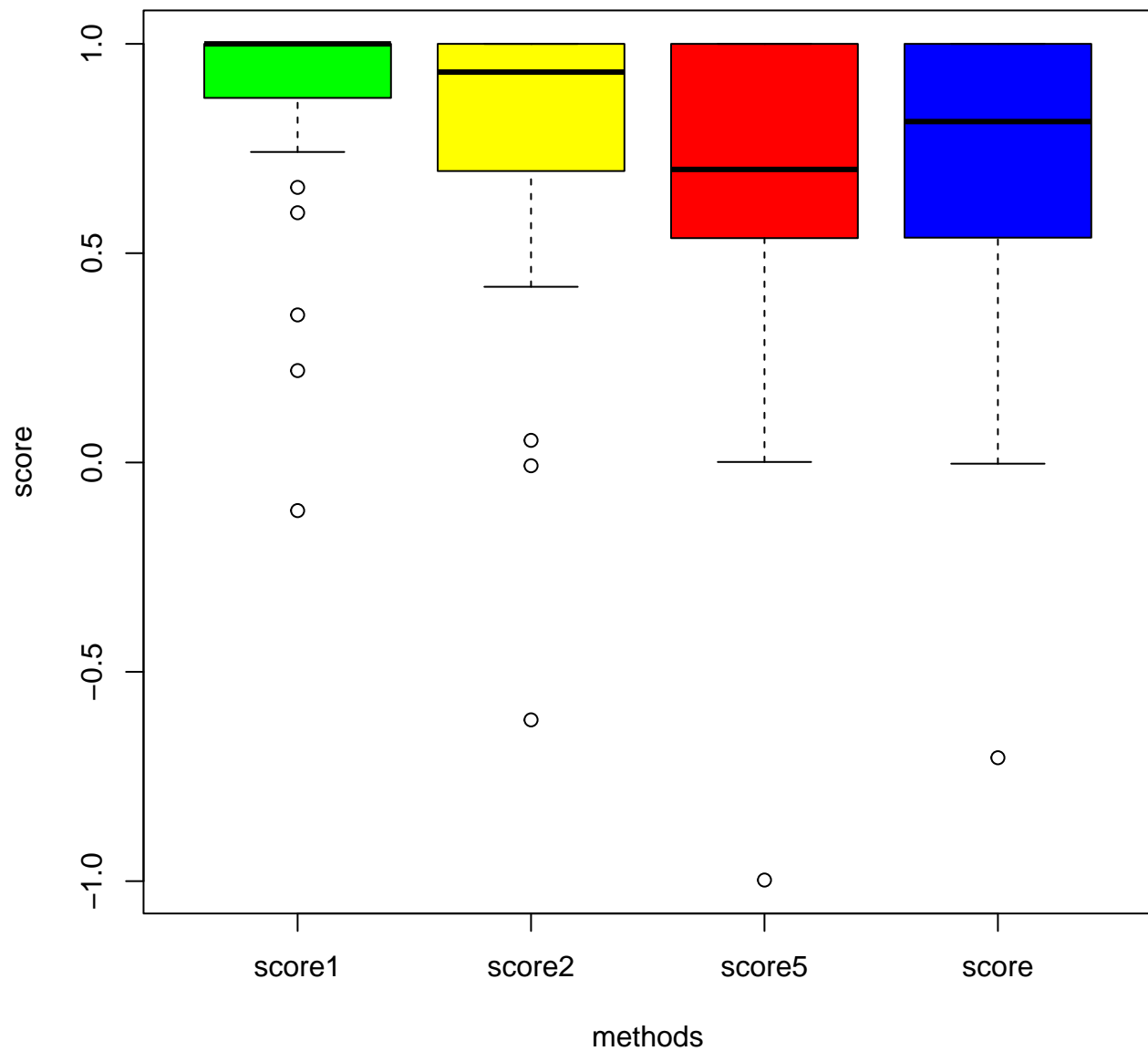

Supplement: Supplementary file 1 [file ijms-25-05267-s001.zip › File S4/2_stability_scores_boxplot/uterine cancer_StabBoxplot.pdf]

# uveal melanoma\_stability analysis

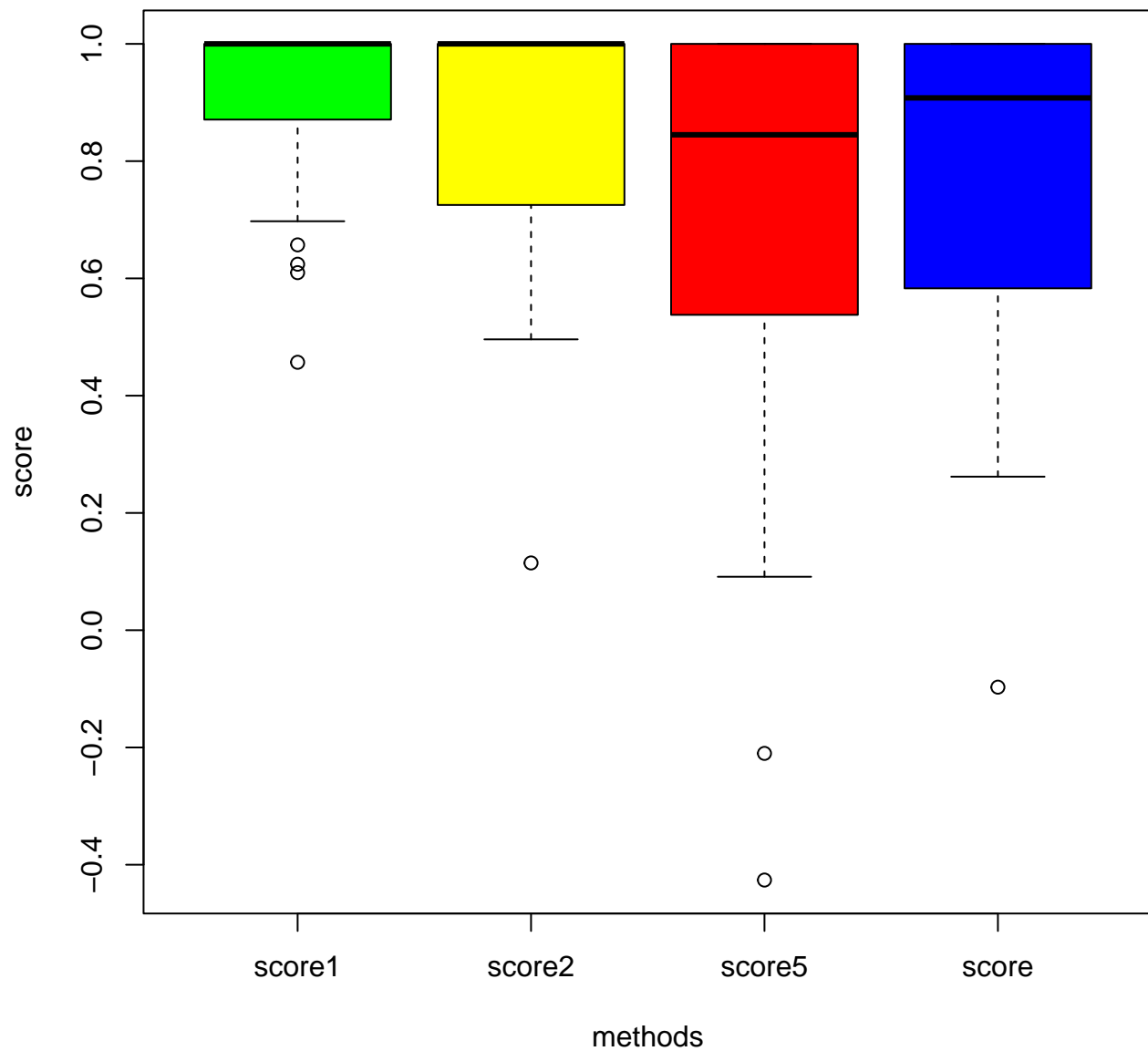

Supplement: Supplementary file 1 [file ijms-25-05267-s001.zip › File S4/2_stability_scores_boxplot/uveal melanoma_StabBoxplot.pdf]
