# Supplementary material for: DDCM: A Computational Strategy for Drug Repositioning Based on Support-Vector Regression Algorithm
Source: Int J Mol Sci. 2024 May 12;25(10):5267. doi: 10.3390/ijms25105267 (PMC11121335; doi:10.3390/ijms25105267)
Supplement: Supplementary file 1 [file ijms-25-05267-s001.zip › File S5/2_stability_scores_boxplot/hepatic vein thrombosis_StabBoxplot.pdf]

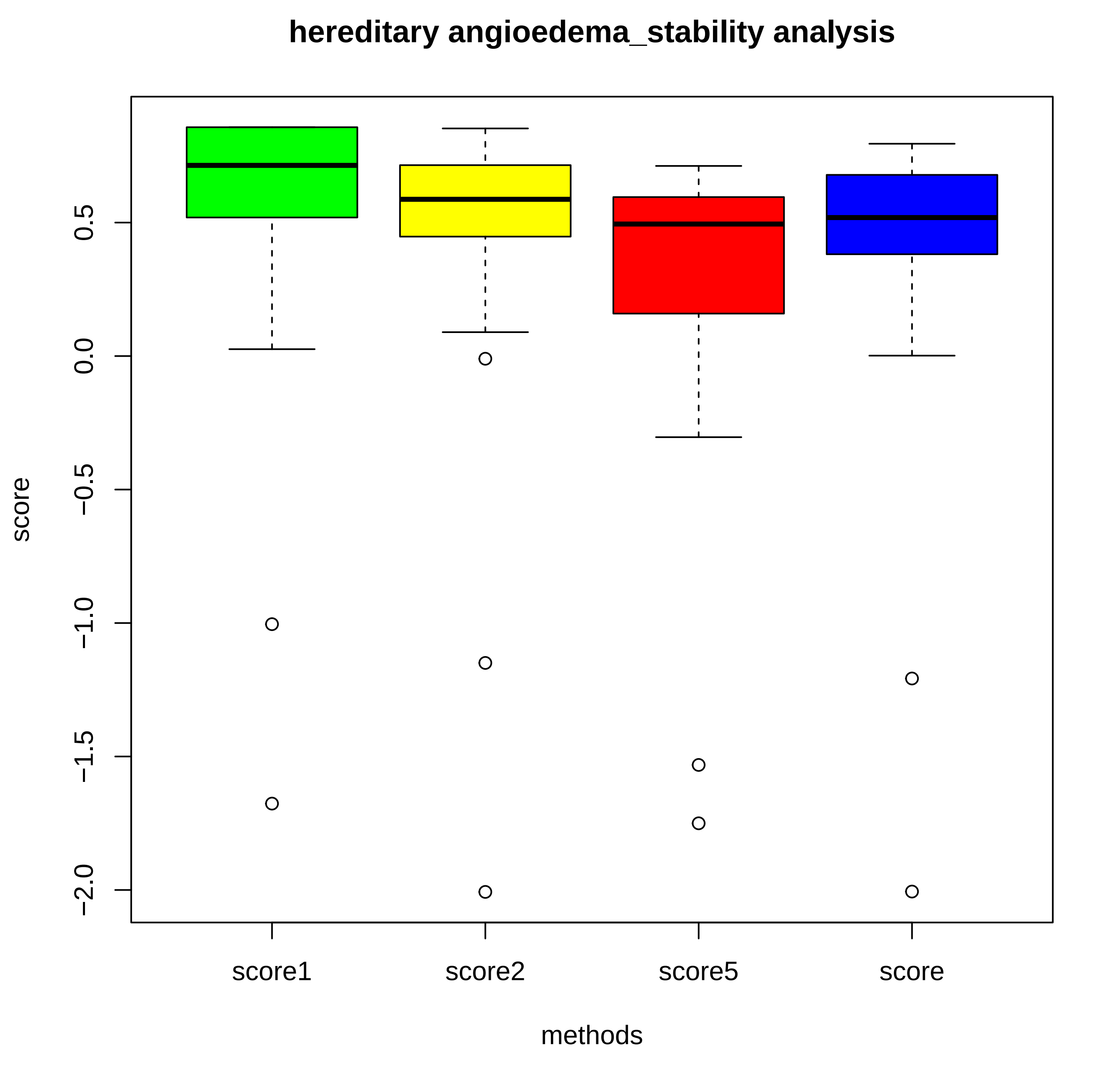
hereditary angioedema_stability analysis
score
methods
score1
score2
score5
score
0.5
0.0
−0.5
−1.0
−1.5
−2.0
